# Integrating Biosynthetic, Genomic and Ecological Open Data for Medicinal Plant Research: A Leakage-Aware Evidence-Prioritization Framework

**DOI:** 10.3390/ijms27156801

**Published:** 2026-07-29

**Authors:** Lidiia S. Samarina, Nina V. Terletskaya, Yury L. Orlov

**Affiliations:** 1 Institute of Genetics and Physiology, Almaty 050060, Kazakhstan; teni02@mail.ru; 2 People’s Friendship University of Russia, Moscow 117198, Russia; 3 Sechenov First Moscow State Medical University, Moscow 119991, Russia

**Keywords:** medicinal plants, specialized metabolism, plant open data, data leakage, taxon–compound associations, evidence prioritization, grouped validation, ecological niche characterization, experimental prioritization

## Abstract

Medicinal plant research increasingly combines heterogeneous public data, but data leakage and unsupported biological inference remain major risks. We developed a leakage-aware framework separating taxon–compound evidence ranking from environmental niche characterization. Six taxa and ten molecules or broad classes formed 60 taxon–compound pairs (27 supported and 33 below-threshold background); primary modeling used 36 specific-molecule pairs (8 supported and 28 unlabeled background) and five compound-matched pathway/chemical predictors. Under leave-one-taxon-out validation, the prespecified balanced random forest achieved a balanced accuracy of 0.621 (95% fold interval: 0.500–0.800; accuracy: 0.694; precision: 0.250; recall: 0.500; permutation: *p* = 0.154). Matched-pathway-only and molecular-weight-only benchmarks achieved 0.662 and 0.358, respectively, and a post hoc logistic comparator achieved 0.646. The cross-molecule balanced accuracy was 0.746 (fold interval: 0.516–0.975). Evidence scores correlated moderately with out-of-fold probabilities (Spearman: ρ = 0.39, *p* = 0.017). Environmental analyses used 248 SoilGrids and 324 NASA POWER taxon × exact-cell rows. The spatially restricted PERMANOVA was non-significant for soil (R^2^ = 0.257, *p* = 0.067) and climate (R^2^ = 0.233, *p* = 0.075), whereas grouped taxon classifiers achieved balanced accuracies of 0.479 and 0.547 (permutation: *p* = 0.005 for both). Environmental-only compound controls were non-significant. The outputs provide an auditable exploratory ranking workflow, but predictive validity for taxon–compound prioritization was not established.

## 1. Introduction

Medicinal plants remain a major source of structurally diverse and pharmacologically relevant natural products. A substantial proportion of approved therapeutic agents are natural products, their derivatives, or synthetic compounds inspired by natural-product scaffolds, demonstrating the continuing importance of biological chemodiversity in drug discovery [1]. Plant specialized metabolites, also referred to as secondary metabolites or phytochemicals, are not merely passive chemical end-products: they contribute to defense, stress adaptation, signaling, development, and interactions with herbivores, microorganisms, and neighboring plants [2]. Their exceptional structural diversity has been associated with enzyme-family expansion, multifunctionality, catalytic promiscuity, pathway diversification, and ecological selection [3,4].

Advances in genomics, transcriptomics, and metabolomics have substantially improved the investigation of medicinal-plant biosynthetic pathways, including the identification of candidate genes, regulatory networks, and biosynthetic gene clusters [5]. Nevertheless, medicinal-plant genomic resources remain highly uneven in their taxonomic coverage, assembly completeness, annotation quality, and representation of known biosynthetic genes [6]. Gene-centered machine learning methods address part of this problem by using genomic, transcriptomic, and other molecular features to distinguish genes associated with specialized metabolism [7,8,9].

Compound-centered computational approaches operate on a different analytical level. Deep transfer learning models can classify molecular structures into plant secondary-metabolic pathway categories using molecular graphs and simplified molecular-input line-entry system representations [10]. Bio-retrosynthetic systems such as BioNavi-NP instead use reaction data, transformer architectures, and multistep search algorithms to reconstruct plausible biosynthetic routes from target natural-product structures to candidate precursors [11]. Natural-product knowledge resources provide another type of output: the LOTUS initiative harmonizes and disseminates referenced structure–organism associations collected from heterogeneous literature and database sources [12]. These methods support molecular classification, pathway reconstruction, and chemical–taxonomic knowledge retrieval, but they do not directly determine whether a particular medicinal taxon combines compound-specific pathway support, adequate genetic-resource availability, and an ecological context suitable for subsequent experimental sampling.

Open biodiversity and environmental resources provide complementary information for medicinal-plant research. Global Biodiversity Information Facility (GBIF) occurrence records can support broad-scale characterization of realized taxon distributions [13], while SoilGrids and NASA POWER provide globally standardized soil, meteorological, and solar-radiation variables for locations lacking field measurements [14,15]. Species-distribution and geographic-information-system models use such data to estimate habitat or cultivation suitability; for example, machine learning approaches have been used to identify soil conditions and geographic areas potentially suitable for medicinal-plant cultivation [16]. Ethnobotanical indices address another prioritization dimension by summarizing use reports, cultural importance, or informant consensus, although these indices do not independently establish pharmacological activity, chemical composition, or biosynthetic capacity [17]. When harmonized phytochemical measurements are available, environmental variables can be linked more directly to medicinal quality [18,19].

Despite these advances, a specific methodological gap remains at the pre-experimental stage of medicinal-plant research. Investigators may need to compare multiple taxon–compound hypotheses when genomic and transcriptomic coverage is uneven, pathway annotations differ in specificity, and harmonized metabolomic measurements are unavailable. In these circumstances, the absence of a reported taxon–compound association cannot be treated as confirmed biochemical absence, and high database coverage may reflect research intensity rather than biosynthetic potential [6]. Occurrence and environmental datasets additionally contain spatial, taxonomic, and hierarchical dependencies that can produce overoptimistic estimates when random row-level validation is used [20,21]. Target-related literature fields, taxon identifiers, compound names, or variables derived from curation decisions can also introduce data leakage and allow a model to reproduce dataset construction rather than learn transferable biological relationships [22]. Consequently, undocumented taxon–compound combinations are more appropriately regarded as unlabeled background observations within a positive–unlabeled learning problem than as experimentally verified negative examples [23].

The methodological contribution of the present study is, consequently, not a new algorithm for identifying metabolites or predicting their concentrations. We introduce a task-decomposed, leakage-aware framework for pre-experimental prioritization of medicinal-plant taxon–compound hypotheses. This study applies this architecture to medicinally relevant taxa representing contrasting genomic, biosynthetic, and ecological contexts, including *Artemisia annua*, *Glycyrrhiza glabra*, *Hypericum*, *Rhodiola*, *Sedum*, and *Taxus*. Its objectives were the following: first, to construct reproducible genetic, pathway, occurrence, soil, and climate evidence profiles; second, to determine whether taxon–compound evidence remains transferable under leakage-screened grouped validation; and, third, to establish which ecological and biochemical interpretations are and are not supported without measured phytochemical outcomes. We hypothesized that biosynthetic-pathway support, genetic-resource availability, and environmental niche structure would provide complementary but analytically non-equivalent information. Specifically, we expected soil and climate variables to differentiate taxon niches but not independently support compound-level inference, consistent with the distinction between broad environmental context and experimentally measured metabolite accumulation.

## 2. Results

### 2.1. Dataset Assembly and Separation of Analytical Units

**Figure 1:**
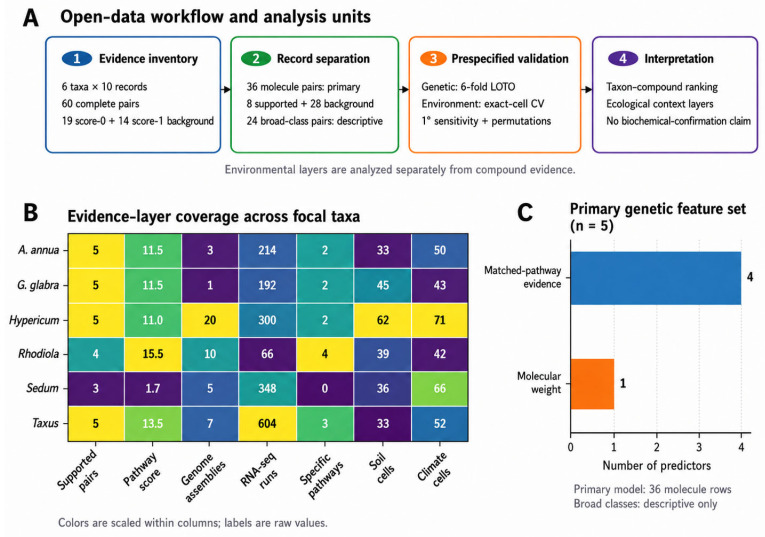
Open-data workflow, record separation and evidence-layer structure. (**A**) The complete evidence inventory contained 60 taxon–compound pairs (6 taxa × 10 records), including 19 score-0 and 14 score-1 below-threshold background rows. The primary genetic analysis was restricted to 36 specific-molecule pairs, whereas 24 broad-class pairs were descriptive only. Genetic evaluation used six-fold leave-one-taxon-out (LOTO) validation; environmental evaluation used exact-cell grouped cross-validation with 1° spatial sensitivity analyses and label permutations. Environmental layers were analyzed separately from compound evidence. (**B**) Taxon-level coverage across seven evidence layers. Cell labels show raw counts or scores, while colors are scaled independently within columns and, therefore, should not be compared across variables measured on different scales. (**C**) The prespecified primary genetic feature set contained four compound-matched pathway variables and molecular weight (*n* = 5). Broad compound classes were excluded from primary modeling because molecular-weight missingness coincided exactly with record type.

The distinct analytical datasets for taxon–compound evidence ranking and environmental niche characterization were generated (Figure 1A; Table 1). The complete genetic/pathway inventory comprised 60 unique taxon–compound pairs formed by six focal taxa and ten molecules or broad compound classes. Twenty-seven pairs met the open-data support criterion. The remaining 33 below-threshold pairs, comprising 19 score-0 and 14 score-1 rows, were treated as unlabeled background rather than as confirmed biochemical absences. Record-type separation then defined 36 specific-molecule pairs (8 supported and 28 background) for primary modeling and retained 24 broad-class pairs (19 supported and 5 background) for descriptive analysis only. The occurrence workflow retrieved 6000 GBIF records, of which 5853 passed the liberal filtering stage and 2281 occupied spatial blocks. For the environmental branches, 1563 SoilGrids-linked occurrences were aggregated to 248 complete taxon × exact-cell rows, and 2274 NASA POWER-linked occurrences were aggregated to 324 complete taxon × exact-cell rows. This separation prevented occurrence-level replication from being conflated with taxon–compound evidence and restricted the environmental analyses to niche characterization.

Evidence coverage differed substantially among taxa (Figure 1B). *Artemisia annua*, *Glycyrrhiza glabra*, *Hypericum* and *Taxus* each had five supported taxon–compound pairs, compared with four for *Rhodiola* and three for *Sedum*. Pathway-support scores were highest for *Rhodiola* (15.5), followed by *Taxus* (13.5), *Artemisia annua* and *Glycyrrhiza glabra* (11.5 each), *Hypericum* (11.0), and *Sedum* (1.7). The evidence layers were not interchangeable: *Taxus* had the largest RNA-seq collection (604 runs), *Hypericum* had the greatest genome-assembly coverage (20 assemblies), and *Rhodiola* had specific evidence in the largest number of pathway groups (four), whereas *Sedum* had no pathway group with specific support. Environmental coverage ranged from 33 to 62 SoilGrids-linked blocks and from 42 to 71 NASA POWER-linked blocks among taxa.

The record-type audit identified an important missingness mechanism. Molecular weight was available for all 36 specific-molecule rows but absent for all 24 broad-class rows; support prevalence also differed sharply between broad classes (19/24, 79.2%) and specific molecules (8/36, 22.2%). Median imputation in a mixed-record model would, therefore, allow molecular weight to act largely as a proxy for record type. To avoid this artefact, the prespecified primary model used only the 36 molecule rows and five variables: four compound-matched pathway features plus molecular weight (Figure 1C). Broad compound classes and the wider set of retained numeric variables were analyzed only descriptively or as secondary benchmarks.

### 2.2. Leakage Audit and Grouped Validation

**Figure 2:**
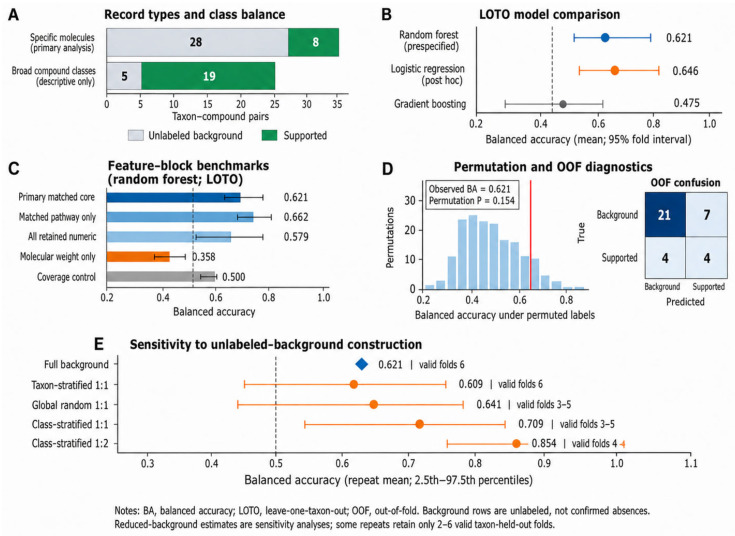
Record-type separation, grouped validation and sensitivity diagnostics for the specific-molecule model. (**A**) The primary dataset comprised 36 specific-molecule pairs (8 supported and 28 below-threshold background), whereas 24 broad-class pairs (19 supported and 5 background) were descriptive only. (**B**) LOTO balanced accuracy for models using the same five-variable primary feature set. Points are the means across six taxon-held-out folds; error bars are empirical 95% fold intervals. The gray dashed line indicates the mean balanced accuracy obtained from the label-permutation null distribution for the prespecified random forest. The diamond identifies the prespecified balanced random forest; logistic regression and gradient boosting are comparators. (**C**) Random forest feature-block benchmarks under LOTO validation. The primary matched core contained four compound-matched pathway variables plus molecular weight. These are task-matched benchmarks, not leave-one-block-out ablations. The gray dashed lines indicate chance-level balanced accuracy (0.500). (**D**) Label-permutation null distribution and pooled out-of-fold confusion matrix for the prespecified primary model. The red line in panel D indicates the observed balanced accuracy (0.621). The observed balanced accuracy was 0.621, and the empirical permutation *p*-value was 0.154 based on 200 permutations. Background rows are unlabeled rather than confirmed biochemical absences. (**E**) Positive–unlabeled sensitivity to alternative background-sampling strategies. Points show the repeat means, and error bars show 2.5th–97.5th percentiles. The gray dashed lines indicate chance-level balanced accuracy (0.500). Reduced-background designs retained only two to six valid LOTO folds and are sensitivity analyses rather than confirmatory estimates.

The modeling audit first separated specific molecules from broad compound classes (Figure 2A). In the complete 60-row inventory, scores of 2–3 defined 27 supported pairs and scores of 0–1 defined 33 below-threshold background pairs. The primary specific-molecule task comprised 36 rows, including 8 supported and 28 background pairs; the 24 broad-class rows were retained only for descriptive summaries. The binary target represents support under the current open-data criteria, not verified presence or absence of a compound. Target-construction variables, identifiers, generic broad enzyme-family counts and record-type indicators were excluded from the prespecified five-variable primary feature set. All preprocessing and model fitting were performed within the training folds of the relevant grouped-validation design.

Under six-fold leave-one-taxon-out (LOTO) validation, the prespecified balanced random forest model achieved a mean balanced accuracy of 0.621 (95% fold interval: 0.500–0.800), accuracy of 0.694, precision of 0.250, recall of 0.500, F1-score of 0.317, Matthews correlation coefficient of 0.191, ROC-AUC of 0.425 and average precision of 0.361 (Figure 2B,D; Table 2). The pooled out-of-fold confusion matrix contained 21 true-background, 7 false-positive, 4 false-negative and 4 true-supported predictions. The observed balanced accuracy was not distinguishable from the label-permutation null distribution (empirical *p* = 0.154; 200 permutations). Using the same five variables and LOTO folds, the post hoc balanced-logistic comparator achieved a balanced accuracy of 0.646 (95% fold interval: 0.500–0.794), accuracy of 0.667, precision of 0.247, recall of 0.667 and F1-score of 0.356, while gradient boosting achieved a balanced accuracy of 0.475.

Feature-block benchmarks did not support the earlier interpretation that molecular weight dominated performance in the molecule-only primary task (Figure 2C; Table 2). The four matched-pathway variables alone achieved a mean LOTO balanced accuracy of 0.662 (95% fold interval: 0.500–0.800), compared with 0.621 for the prespecified five-variable matched core, 0.579 for all retained numeric variables, 0.358 for molecular weight alone and 0.500 for the coverage-control block. Thus, the molecular-weight artefact arose principally when broad classes and specific molecules were mixed; within the molecule-only task, the matched-pathway variables provided the stronger benchmark signal.

When each specific molecule was held out in turn, random forest balanced accuracy was 0.746 (95% fold interval: 0.516–0.975; six valid folds) (Figure 2; Table 2). In this molecule-only set, leave-one-compound-out and leave-one-compound-class-out validation are equivalent because every molecule has a unique class. The wide interval indicates substantial between-molecule uncertainty. Positive–unlabeled sensitivity analysis further qualified the primary estimate (Figure 2E). With all 28 molecule-level background rows, the balanced accuracy was 0.621 across six valid LOTO folds. The mean values were 0.609 for taxon-stratified 1:1 sampling, 0.641 for global-random 1:1 sampling, 0.709 for compound-class-stratified 1:1 sampling and 0.854 for compound-class-stratified 1:2 sampling. However, reduced-background designs retained only two to six valid taxon-held-out folds, and their percentile ranges were wide.

### 2.3. Evidence Profiles and Prioritization Diagnostics

**Figure 3:**
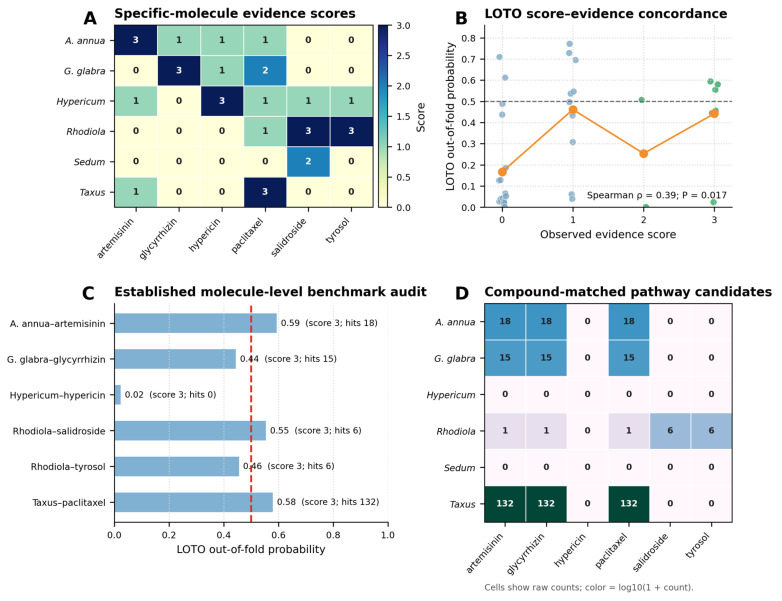
Specific-molecule evidence structure and experimental-prioritization audit. The gray dashed lines indicate chance-level balanced accuracy (0.500). (**A**) Open-data evidence scores for 36 specific-molecule taxon–compound pairs. Scores of 0–1 denote below-threshold background and scores of 2–3 denote supported evidence; background is not confirmed biochemical absence. (**B**) Concordance between evidence score and prespecified random forest LOTO out-of-fold probability. Small points show individual pairs; orange points and lines show the mean probability at each evidence-score level. Spearman: ρ = 0.39 (*p* = 0.017); the dashed line marks the 0.5 model threshold. (**C**) Audit of six established molecule-level associations. Bars show LOTO out-of-fold probabilities; labels indicate the evidence score and compound-matched pathway-candidate count. (**D**) Compound-matched pathway-candidate counts for each taxon and specific molecule. Cell labels are raw counts, and color represents log10(1 + count). Shared pathway mappings can yield repeated taxon-level counts across molecules; the matrix represents public pathway evidence, not biochemical confirmation.

Observed evidence scores for the 36 specific-molecule pairs ranged from zero to three (Figure 3A). Within this molecule-only set, the association between observed evidence score and the prespecified random forest LOTO out-of-fold probability was positive but moderate (Spearman: ρ = 0.39, *p* = 0.017; Figure 3B). The mean probabilities were lowest at score 0, higher at score 1, lower again for the small score-2 group and higher at score 3, showing substantial within-score dispersion.

The established molecule-level benchmark audit showed heterogeneous taxon-held-out ranking (Figure 3C). *Artemisia annua*–artemisinin, *Glycyrrhiza glabra*–glycyrrhizin, *Hypericum*–hypericin, *Rhodiola*–salidroside, *Rhodiola*–tyrosol and *Taxus*–paclitaxel each had the maximum observed evidence score of three, but their LOTO out-of-fold probabilities were 0.594, 0.444, 0.023, 0.554, 0.456 and 0.580, respectively. *Artemisia annua*–artemisinin, *Rhodiola*–salidroside and *Taxus*–paclitaxel exceeded the 0.5 threshold, whereas the other three did not. Compound-matched pathway-candidate counts ranged from 0 for *Hypericum*–hypericin to 132 for *Taxus*–paclitaxel.

The compound-matched pathway matrix made the taxon–compound interaction explicit (Figure 3D). Candidate counts were highest for *Taxus*–paclitaxel (132), followed by *Artemisia annua*–artemisinin (18), *Glycyrrhiza glabra*–glycyrrhizin (15) and *Rhodiola*–salidroside or –tyrosol (6 each), whereas no matched candidates were retrieved for *Hypericum*–hypericin. Compounds mapped to the same pathway group can share a taxon-level candidate count, so these values indicate available pathway-specific public evidence rather than proof that a candidate enzyme produces the named molecule.

### 2.4. Soil and Climate-Associated Environmental Profiles

**Figure 4:**
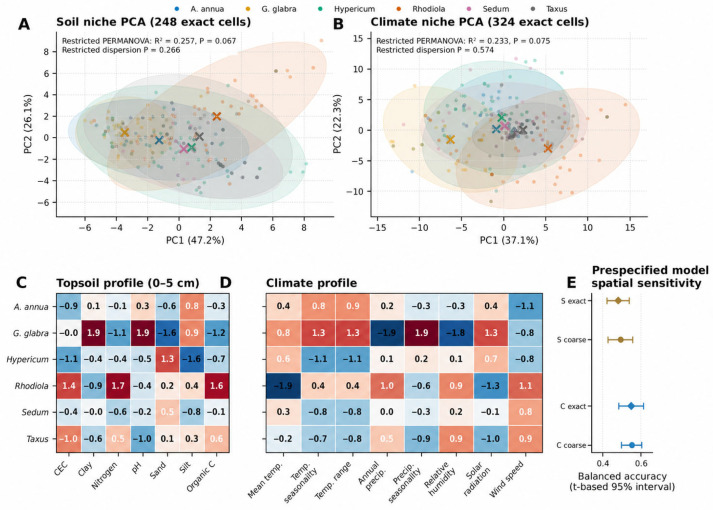
Soil and climate niche structure and spatial-validation sensitivity. (**A**) PCA of 248 taxon × exact-cell SoilGrids rows. Restricted PERMANOVA resulted in an R^2^ = 0.257 and *p* = 0.067; restricted dispersion, *p* = 0.266. (**B**) PCA of 324 taxon × exact-cell NASA POWER rows. Restricted PERMANOVA resulted in an R^2^ = 0.233 and *p* = 0.075; restricted dispersion, *p* = 0.574. Labels were permuted within 1° spatial clusters for both restricted tests. Points are exact cells, colors denote taxa, crosses denote taxon centroids and shaded ellipses summarize within-taxon dispersion. (**C**) Taxon-level topsoil profiles for seven SoilGrids variables at a 0–5 cm depth. (**D**) Taxon-level climate profiles for eight NASA POWER summaries. Heatmap values are feature-wise z-scores across taxa. (**E**) Prespecified-model sensitivity to spatial grouping. “S exact” and “C exact” denote GroupKFold validation by exact 0.25° cell for soil and climate; “coarse” denotes grouping by 1° spatial cluster. Points show the mean balanced accuracy, and error bars are t-based 95% intervals. Exact-cell grouping prevents cell overlap but does not guarantee geographic separation of adjacent cells.

At the taxon × exact-cell scale, the first two principal components explained 73.3% of the variation in the SoilGrids feature set (PC1, 47.2%; PC2, 26.1%) and 59.4% of variation in the NASA POWER feature set (PC1, 37.1%; PC2, 22.3%) (Figure 4A,B). With taxon-label permutations restricted within 1° spatial clusters, the global PERMANOVA did not reject the null for either layer (soil: R^2^ = 0.257, *p* = 0.067; climate: R^2^ = 0.233, *p* = 0.075). Restricted multivariate-dispersion tests were also non-significant (soil: *p* = 0.266; climate: *p* = 0.574). The ordinations, therefore, show descriptive taxon-associated environmental structure, while the restricted multivariate tests do not provide confirmatory evidence of global centroid separation.

The standardized profiles identified the environmental variables underlying these contrasts (Figure 4C,D). In the soil layer, *Glycyrrhiza glabra* had a comparatively high clay content and pH but low sand, nitrogen and soil organic carbon; *Rhodiola* had a high cation-exchange capacity and nitrogen and soil organic carbon contents; *Hypericum* had high sand and low silt contents; and *Taxus* had a comparatively high cation-exchange capacity and nitrogen and soil organic carbon contents together with low pH. *Artemisia annua* was distinguished by a relatively high silt content, whereas *Sedum* showed comparatively modest deviations across most topsoil variables. In the climate layer, *Glycyrrhiza glabra* had high temperature and precipitation seasonality, high solar radiation, low annual precipitation and low relative humidity. *Rhodiola* occupied the coldest, wetter, more humid and windier profile, whereas *Taxus* also showed relatively high precipitation and humidity with low solar radiation. *Artemisia annua* had relatively high temperature seasonality and range but low wind, while *Hypericum* and *Sedum* had lower temperature seasonality and range than several other taxa.

Exact-cell grouped classification detected taxon-associated niche signal above the six-class chance reference of 0.167 (Figure 4E; Table 2). The prespecified soil random forest achieved a mean balanced accuracy of 0.479 (t-based 95% interval: 0.422–0.537), accuracy of 0.512, macro precision of 0.443, macro recall of 0.479, macro F1 of 0.437, MCC of 0.417 and macro ROC-AUC of 0.812. The prespecified climate multinomial logistic model achieved a balanced accuracy of 0.547 (0.483–0.612), accuracy of 0.515, macro precision of 0.539, macro recall of 0.547, macro F1 of 0.510, MCC of 0.433 and macro ROC-AUC of 0.815. Both models exceeded their taxon-label permutation null distributions (empirical *p* = 0.005). Grouping at the coarser 1° scale produced similar balanced accuracies of 0.492 (0.427–0.557) for soil and 0.551 (0.498–0.604) for climate. Exact-cell grouping prevents the same 0.25° cell from appearing in training and test sets but can place adjacent cells in different folds; the 1° analysis, therefore, provides a more conservative spatial-sensitivity check.

Repeated equal-size block subsampling evaluated sensitivity to unequal taxon sample sizes (100 repetitions; 33 rows per taxon for soil and 42 for climate; Supplementary Table S6A). The median restricted PERMANOVA R^2^ was 0.261 (2.5th–97.5th percentiles 0.235–0.287) for soil and 0.267 (0.257–0.281) for climate; PERMANOVA *p* < 0.05 occurred in 8% and 6% of repetitions, while dispersion *p* < 0.05 occurred in 4% and 0%, respectively. A common auxiliary grouped multinomial logistic classifier, used for a model-family-consistent sensitivity diagnostic and distinct from the primary soil random forest, produced median balanced accuracies of 0.434 (0.379–0.487) for soil and 0.539 (0.497–0.576) for climate.

### 2.5. Environmental Negative Controls and Inference Boundary

**Figure 5:**
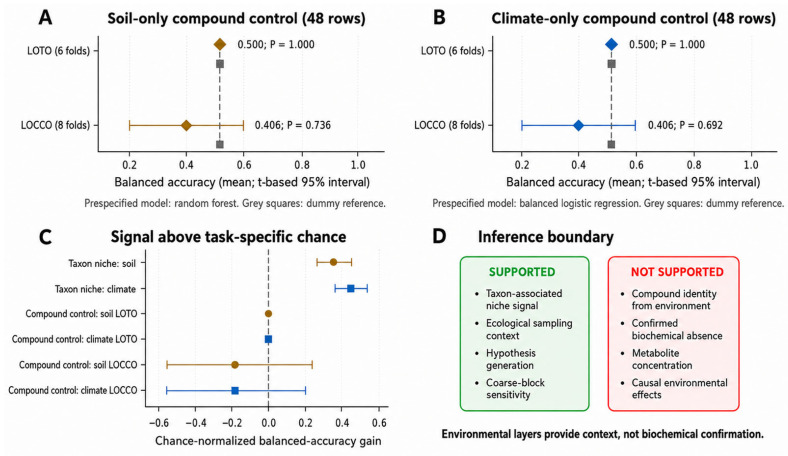
Environmental compound controls and the resulting inference boundary. (**A**) Soil-only compound-evidence control using the prespecified random forest. (**B**) Climate-only compound-evidence control using the prespecified balanced logistic regression. Each dataset contained 48 rows (6 taxa × 8 shared annotations; 10 supported and 38 background). Diamonds show balanced accuracy and t-based 95% intervals; grey squares show the most-frequent-class dummy reference. Prespecified models achieved 0.500 under six-fold LOTO (*p* = 1.000) and 0.406 under eight-fold LOCCO (soil *p* = 0.736; climate *p* = 0.692). (**C**) Chance-normalized balanced-accuracy gain, calculated as (balanced accuracy − chance)/(1 − chance), using 1/6 for six-class taxon-niche classification and 0.5 for binary controls. Positive taxon-niche gains contrast with zero LOTO and negative LOCCO control gains. (**D**) Supported inference boundary. Environmental layers provide taxon-associated niche context, sampling guidance, hypothesis generation and coarse-block sensitivity analyses; they do not establish compound identity, confirmed biochemical absence, metabolite concentration or causal environmental effects.

The compound-level environmental controls did not support direct inference from soil or climate to taxon–compound evidence (Figure 5A,B; Table 2). Each control contained 48 rows representing six taxa × eight shared compound annotations, including 10 supported and 38 background rows. Under six-fold LOTO validation, the prespecified soil random forest and climate balanced logistic regression each produced a balanced accuracy of 0.500 (95% interval: 0.500–0.500; empirical *p* = 1.000). Under eight-fold LOCCO validation, both produced a mean balanced accuracy of 0.406 (t-based 95% interval: 0.206–0.606); the permutation *p*-values were 0.736 for soil and 0.692 for climate. The dummy reference remained at 0.500.

After accounting for the different chance levels of the six-class niche and binary compound-control tasks, the chance-normalized gain was 0.375 for the soil taxon-niche model and 0.457 for the climate taxon-niche model, compared with 0 under LOTO and −0.188 under LOCCO for both environmental compound controls (Figure 5C). This contrast shows that the same environmental layers contained taxon-associated niche information but did not provide transferable compound-level evidence.

The supported inference boundary is, therefore, deliberately narrow (Figure 5D). SoilGrids and NASA POWER variables support taxon-level environmental characterization, taxon-associated classification signal, ecological sampling context and hypothesis generation. They do not support compound identity, confirmed biochemical absence, metabolite presence or concentration, causal soil or climate effects, or habitat suitability without a separate suitability analysis. The environmental layers are, consequently, interpreted as ecological context rather than direct predictors of plant chemistry.

## 3. Discussion

### 3.1. What the Leakage-Aware Model Learned—And Did Not Learn

Generally, the results indicate unstable discrimination in a small, hierarchically structured dataset rather than confirmed generalization. The out-of-fold probability should, therefore, be treated as an exploratory prioritization score, not as a prediction of metabolite presence or biosynthetic capacity [20,21,22,23]. Record-type separation changed the interpretation of molecular weight. After restricting the primary task to specific molecules, molecular weight alone performed poorly, whereas the four compound-matched pathway variables produced the stronger benchmark. Within the molecule-only dataset, pathway-group-matched variables contributed more strongly than molecular weight; however, shared pathway mappings limited discrimination among molecules belonging to the same broad biosynthetic group. Gene- and pathway-centered methods operate at finer biological units and remain necessary for mechanistic inference [3,4,7,8,9,10].

Within the specific-molecule set, the positive association between curated evidence score and LOTO probability (ρ = 0.39, *p* = 0.017) indicates partial concordance, but the wide dispersion within score levels limits its practical precision. The six established benchmark associations illustrate this directly: their probabilities ranged from 0.023 to 0.594, and only three exceeded 0.5 despite every pair having an evidence score of 3. Named-molecule biosynthesis depends on specific enzyme sequences, regulation, tissue context and developmental state, so pathway-candidate counts alone cannot establish metabolite production. Conversely, a low score or probability may reflect missing public data rather than absent biosynthetic potential. The framework, therefore, supports traceable, pathway-oriented hypothesis generation while requiring direct chemical and molecular validation [3,4,7,8,9,10].

The variable benchmark rankings should not be confused with uncertainty about the underlying biology. Experimental studies have established key enzymes and genetic determinants of artemisinin biosynthesis in *Artemisia annua* and have demonstrated heterologous production of artemisinin precursors and artemisinin [24,25,26,27]. Taxol biosynthesis and taxadiene synthase have, likewise, been characterized in *Taxus* [28,29]; cytochrome P450 steps in glycyrrhizin biosynthesis have been resolved in licorice [30,31]; the salidroside pathway has been elucidated and reconstituted for *Rhodiola* [32]; and hypericin is a documented constituent of *Hypericum perforatum* extracts [33]. These studies supply external knowledge for defining the benchmark associations, but the present model recovered them unevenly under taxon-held-out validation.

### 3.2. Environmental Niche Signal and Its Inference Boundary

The environmental analyses answered a different question from the taxon–compound model. The PCA showed descriptive taxon-associated environmental structure, and the exact-cell grouped classifiers detected taxon-associated classification signals above their permutation nulls. However, the spatially restricted PERMANOVA tests did not reject the null for either soil or climate and, therefore, did not provide confirmatory evidence of global multivariate centroid separation.

The environmental negative controls define the strongest inferential boundary in the study. Soil- and climate-only models were at chance under LOTO validation and below chance on average under LOCCO validation, and none exceeded the corresponding permutation null. Thus, the environmental layers provide no evidence for compound identity, metabolite presence or concentration, biochemical absence, or a causal effect of soil or climate on biosynthesis. This differs from habitat-suitability studies, which define an explicit geographic suitability outcome, and from medicinal-plant quality studies that combine environmental predictors with measured chemical constituents [16,18]. Environmental regulation of specialized metabolism is biologically plausible and well documented in principle [2,19], but it was not tested directly here because the occurrence records were not linked to metabolomic measurements. Accordingly, the niche models should be used to design geographically or environmentally contrasted sampling, not to identify high-yield habitats or infer chemical quality.

Several sources of ecological uncertainty remain. Public occurrence records are affected by sampling effort, accessibility, taxonomic identification and spatial clustering; soil and climate values inherit the resolution and uncertainty of the underlying gridded products; and geographic differences among taxa may be confounded with unmeasured history, dispersal and biotic interactions. Grouping by exact 0.25° cell prevents the same cell from appearing in both the training and test data but can still split adjacent cells; the 1° grouping and within-cluster permutation analyses provide sensitivity checks rather than complete control of spatial autocorrelation. These procedures reduce optimistic estimates but do not remove bias or establish causation [14,15,20,21]. Independent occurrence data and, for chemical questions, field or common-garden sampling linked to measured metabolites are required before extending the environmental results beyond niche context.

### 3.3. Relationship to Existing Approaches and Practical Experimental Use

Existing computational approaches operate at different analytical levels. Cross-species and multi-omics methods predict candidate specialized-metabolism genes [7,8], plantiSMASH detects biosynthetic gene clusters [9], molecular and pathway-oriented methods classify transformations or reconstruct potential biosynthetic routes [10,11], and LOTUS organizes previously reported taxon–compound associations [12]. By contrast, the present framework does not predict a biosynthetic gene, reconstruct a pathway or establish metabolite presence. Its contribution is the leakage-aware integration and auditing of heterogeneous evidence for experimental prioritization. Therefore, a direct comparison of accuracy values would be invalid because the methods have different outcomes, labels and validation units.

For hypothesis-driven natural-product research, candidate prioritization should begin with the experimental question rather than with the highest composite score. A practical workflow is as follows: (i) define a named molecule or pathway and the intended assay; (ii) inspect each evidence layer separately, including specific pathway candidates, sequencing-resource coverage, reported chemistry and environmental coverage; (iii) flag whether low evidence reflects a biological contradiction or simply missing public data; and (iv) select a small, contrastive set for independent testing. A taxon with literature-supported chemistry and specific pathway candidates is suitable for targeted gene-expression or enzyme assays. A taxon with reported medicinal use or chemistry but poor genomic and pathway coverage is a rational priority for sequencing and metabolomic resource generation. A taxon spanning distinct environmental blocks is suitable for multi-site chemical sampling, provided that tissue, phenology and analytical method are standardized. The evidence table and provenance should remain available with the shortlist so that each decision is traceable and reusable [5,6,12,13,17,34].

This workflow also changes how understudied taxa should be interpreted. A low public-evidence score—such as the sparse pathway profile observed for *Sedum*—does not establish low biosynthetic potential and should not automatically lower biological priority. It may, instead, identify an information gap. For resource management or sequencing prioritization, the objective can, therefore, be reversed: select taxa expected to yield the largest information gain, while using a data-rich benchmark taxon as a positive process control. For experimental discovery, an understudied candidate should be supported by an independent rationale, such as ethnobotanical evidence, preliminary chromatography, phylogenetic proximity to a producing lineage, or a specific biosynthetic-gene candidate. A concrete *Sedum* case study would, therefore, prespecify one target supported by independent literature or pilot chromatography, measure it across replicated tissues or populations, and generate matched RNA-seq only after compound-positive and compound-negative samples have been identified. Pairing such a data-poor taxon with a data-rich control under the same protocol would help distinguish biological differences from database-coverage effects and would make the framework more useful for the hypothesis-driven workflow emphasized by the reviewers.

At the individual taxon–compound level, an association should be advanced for experimental validation only when it is connected to a clearly defined biological or chemical hypothesis. At least one compound-specific source of prior evidence—such as reported chemical detection, a pathway-specific gene candidate, preliminary chromatography or a relevant ethnobotanical observation—should be present. Molecular weight, generic enzyme-family counts, environmental similarity or overall database coverage should not be sufficient on their own. Among associations meeting this minimum requirement, priority should be given to candidates that can be evaluated with available tissues and authentic standards, include an appropriate positive control, and are expected to resolve a clearly identified evidence gap. The validation method should match that gap: targeted LC–MS or HPLC for uncertain chemistry, transcriptomic or expression analysis for uncertain regulation, enzyme assays for uncertain biochemical function, and replicated multi-site sampling only when an environmental hypothesis is being tested [5,6,12,13,17,35].

### 3.4. Limitations and Next Steps

The principal limitation is that response labels summarize curated public evidence rather than newly measured chemistry. Below-threshold rows are not confirmed biochemical absences, and both positive labels and feature coverage are influenced by research attention. The complete inventory is small (six taxa and ten records), while the primary predictive task contains only 36 molecule rows and eight supported pairs. Separating broad classes removed the molecular-weight missingness proxy, and compound-matched pathway variables introduced an explicit taxon–compound interaction; nevertheless, many resource-coverage variables remain taxon-level quantities repeated across molecules. Positive–unlabeled sensitivity estimates varied with background construction and sometimes retained only two or three valid folds. Exact-cell environmental validation can also place adjacent cells in different folds, and restricted multivariate tests did not reject their null hypotheses. Pathway annotations remain incomplete, and the model does not represent phylogenetic dependence, tissue specificity, developmental stage or gene expression. Most importantly, no metabolomic, transcriptomic or targeted biochemical experiment was conducted. The framework generates auditable priorities; it does not experimentally validate them [13,23].

The focal inputs included both species- and genus-level taxa. Genus-level records aggregate environmentally and genetically heterogeneous species, and their coverage metrics and realized environmental profiles are, therefore, not strictly comparable with species-level records. The present analysis should be interpreted as an input-taxon proof of concept rather than a rank-standardized comparative niche study.

A decisive next step is prospective validation on observations that are independent of the data used for label construction and feature extraction. Candidate taxa should be sampled across replicated populations and, where environmental hypotheses are tested, across deliberately contrasted sites or controlled conditions. The same organs, developmental stages, seasons and post-harvest procedures should be used, followed by targeted LC–MS or HPLC with authentic standards and appropriately reported quality control. Matched RNA sequencing, quantitative expression assays and targeted characterization of specific candidate enzymes would then test whether pathway evidence predicts the measured phenotype. Evaluation should retain the molecular-weight comparator, stratify specific molecules from broad classes, and use external taxon-, phylogeny- or pathway-blocked test sets. Metabolomics reporting and metabolite-identification confidence should follow established minimum standards [35]. Such a design would provide the independent validation requested by the reviewers and would determine whether biological evidence adds predictive value beyond generic chemical structure.

## 4. Materials and Methods

### 4.1. Study Design and Analytical Objective

This study developed a reproducible open-data framework for integrating genetic-resource availability, biosynthetic-pathway evidence, occurrence-derived geographic information, soil properties and climate variables for medicinal-plant research. The framework was designed for pre-experimental settings in which harmonized metabolomic measurements or newly generated genomic data are not available. Its purpose was to rank the strength of publicly available evidence for taxon–compound associations and to characterize the environmental niches of the selected taxa. It was not designed to confirm metabolite presence or absence, predict metabolite concentration, or infer causal environmental effects.

The principal biological units were six medicinally relevant genus- or species-level taxa representing contrasting biosynthetic and ecological contexts, as follows: *Artemisia annua*, *Glycyrrhiza glabra*, *Hypericum*, *Rhodiola*, *Sedum* and *Taxus*. Five additional family-level records (Asteraceae, Crassulaceae, Nitrariaceae, Orobanchaceae and Polygonaceae) were retained only as hierarchical context in the genetic-data branch and excluded from the predictive modeling. The genetic/pathway analysis considered the following ten molecules or broad compound classes: salidroside, tyrosol, hypericin, artemisinin, glycyrrhizin, paclitaxel, flavonoids, phenolic compounds, alkaloids and terpenoids. The environmental negative-control tables contained the following eight annotations shared by the soil and climate branches: salidroside, tyrosol, hypericin, artemisinin, glycyrrhizin, paclitaxel, flavonoids and phenolic compounds. Three analytical units were kept separate throughout the workflow. The genetic/pathway evidence model used one row per taxon–compound pair. The primary soil- and climate-niche models used one row per taxon × spatial block. Compound-expanded environmental tables were used only as negative controls. This separation prevented the replication of identical environmental measurements across compounds from being interpreted as independent compound-level evidence. Table 3 compares the present framework with representative computational approaches used in plant specialized-metabolism and medicinal-plant research. Because these approaches address different analytical units and outcomes, their reported accuracy values were not compared directly with the performance of the present taxon–compound evidence model.

### 4.2. Software Environment and Reproducibility

The analysis was implemented as three linked notebook branches, as follows: (i) genetic-resource and biosynthetic-pathway evidence construction, (ii) SoilGrids soil-niche construction and (iii) NASA POWER climate-niche construction. Each branch comprised a data-acquisition notebook, a feature-construction and quality-control notebook and a modeling notebook. The modeling notebooks used frozen input tables rather than requiring live API access. API responses were cached as JSON files, while tabular intermediates and final analytical tables were exported in TSV and Parquet formats.

All stochastic procedures used a fixed seed of 42. The final genetic and soil modeling runs exported JSON configuration files; climate settings are recorded in the executed notebook and accompanying manifests. Across branches, the preserved artifacts include run identifiers or timestamps, source and handoff manifests, quality-control outputs, feature matrices, validation-group tables, model summaries, figure source tables, file manifests and software-environment records, where generated. Together with the frozen inputs, these files permit reconstruction of the reported analyses without repeating live API queries.

### 4.3. Taxon and Compound Dictionaries

A manually curated taxon dictionary was used to harmonize taxonomic identifiers, accepted scientific names, taxonomic rank, family and modeling role. Genus- and species-level records were designated as primary candidate taxa, whereas family-level records were used only as hierarchical context. Taxon names were resolved against NCBI Taxonomy, and NCBI Entrez E-utilities were used to search and retrieve taxonomic, nucleotide, assembly, protein and literature metadata [36].

A compound dictionary was constructed for the target medicinal molecules and broad compound classes. Individual molecules were queried through the PubChem PUG–REST interface to retrieve standardized compound identifiers, molecular formulae and molecular weights [37]. Flavonoids, phenolic compounds, alkaloids and terpenoids were retained as broad annotation classes rather than treated as single chemical entities. PubChem compound identifiers were used for data retrieval and provenance only and were excluded from predictive modeling.

### 4.4. Open-Source Genetic and Biosynthetic-Pathway Data Collection

The genetic branch quantified the availability and specificity of public genetic evidence for each taxon and pathway group. Retrieval was metadata-first: bulk genome packages and large sequence downloads were disabled by default, while searchable manifests were generated for nucleotide records, assemblies, sequencing runs, candidate proteins, literature records and pathway-gene evidence.

Public sequence and genome evidence were collected from NCBI Nucleotide, NCBI Assembly, NCBI Protein and PubMed through Entrez search and retrieval operations [36]. ENA was queried for run-level RNA-sequencing, whole-genome sequencing and other sequencing metadata [38]. UniProtKB was queried for protein sequences, functional annotations, taxonomic information and cross-references [39]. KEGG was used to obtain manually curated biochemical-pathway context and pathway identifiers [40]. PubChem supplied the chemical metadata described above [37].

Public marker-sequence availability was summarized using chloroplast or plastid, ITS and barcode-like nucleotide records. Marker evidence was kept separate from genome-assembly and transcriptome availability. Genome-assembly metadata were retrieved from NCBI Assembly, and RNA-sequencing and whole-genome sequencing availability was summarized from ENA run metadata. Candidate biosynthetic proteins were retrieved from UniProtKB and NCBI Protein using pathway-specific gene-family queries.

Five pathway groups were prespecified. The salidroside/tyrosol group included 4-hydroxyphenylacetaldehyde synthase, 4-hydroxyphenylacetaldehyde reductase, tyrosol glucosyltransferase and UDP-glycosyltransferase terms. The phenylpropanoid/flavonoid group included PAL, C4H, 4CL, CHS, CHI, F3H, FLS, DFR, ANS, UGT and OMT. The terpenoid/triterpenoid group included DXS, DXR, HMGR, TPS, CYP450, DBAT, BAPT, UGT and OSC. The alkaloid group included NCS, STR, BBE, OMT, CYP450, AAT and TDC. The hypericin-related group included PKS, Hyp-1, CYP450, peroxidase and UGT terms.

Candidate records were assigned evidence tiers according to taxonomic and functional specificity. Taxon-specific records matching a pathway-specific query were treated as stronger evidence than family-level records or matches to generic enzyme families. Counts based on generic terms, such as UGT, CYP450 or TPS, were retained for descriptive summaries but were excluded from the primary classifier because these broad families are not sufficiently specific to establish support for a particular medicinal pathway.

### 4.5. Literature-Derived Target Evidence

The initial taxon dictionary contained 11 taxonomic entries and the compound dictionary contained ten molecules or broad compound classes, generating 110 candidate combinations. The 50 combinations involving five family-level context records were excluded from modeling. The complete focal inventory, therefore, contained 60 rows representing six taxa × ten records. A prespecified record-type audit then separated 36 specific-molecule pairs for primary predictive analysis from 24 broad-class pairs retained for descriptive analysis only.

Evidence was encoded on a four-level ordinal scale. Curated benchmark associations were assigned a score of 3. Associations classified during the literature curation as directly supported or quantitatively curatable were assigned a score of 2. Weak, indirect or contextual evidence was assigned a score of 1, and combinations without usable support under the prespecified open-data criteria were assigned a score of 0. Scores of 2–3 formed the supported class, and scores of 0–1 formed the below-threshold unlabeled background. The complete inventory contained 27 supported and 33 background rows (19 score-0 and 14 score-1). Within the primary molecule-only table, 8 rows were supported and 28 were background; the broad-class descriptive table contained 19 supported and 5 background rows. Background status indicated that the evidence threshold was not met and was not interpreted as experimental evidence of biochemical absence.

Six established molecule-level associations were prespecified as a biological recovery audit, as follows: *A. annua*–artemisinin, *G. glabra*–glycyrrhizin, *Hypericum*–hypericin, *Rhodiola*–salidroside, *Rhodiola*–tyrosol and *Taxus*–paclitaxel. These associations were used to examine whether out-of-fold model scores recovered well-established biological examples; they were not used to select predictors or tune hyperparameters and were not treated as independent experimental validation.

The soil and climate branches used a separate 48-row annotation table comprising six taxa × eight shared compound or compound-class annotations. This table was used only for environmental negative-control analyses and was not used as the response in the primary taxon-niche models.

### 4.6. GBIF Occurrence Retrieval and Geospatial Quality Control

GBIF occurrence records were retrieved separately for the six primary taxa using accepted GBIF taxon keys and hasCoordinate = true [13]. Retrieval was capped at 1000 records per taxon. Pagination used requests of at most 300 records, with the final request reduced to the number required to reach but not exceed the cap. A total of 6000 records were retrieved. Records were deduplicated using the unique GBIF occurrence key, and the number of records before and after deduplication was logged for every taxon.

Latitude and longitude were converted to numeric values. Records outside −90 to 90° latitude or −180 to 180° longitude, records with zero coordinates and records carrying prespecified serious geospatial issue codes were excluded. The excluded issue codes were ZERO_COORDINATE, COORDINATE_OUT_OF_RANGE, COORDINATE_INVALID, COUNTRY_COORDINATE_MISMATCH, PRESUMED_NEGATED_LATITUDE, PRESUMED_NEGATED_LONGITUDE and PRESUMED_SWAPPED_COORDINATE.

The primary liberal dataset retained records with a coordinate uncertainty ≤10,000 m or missing coordinate uncertainty. Missing uncertainty was retained as an explicit indicator where relevant. The liberal filter retained 5853 unique occurrence records. A strict sensitivity dataset applied a threshold of ≤1000 m. A specimen-focused sensitivity subset additionally retained preserved specimens, material samples and living specimens. Because this specimen-focused subset did not adequately represent all six taxa, it was not used as the primary environmental dataset.

Liberal-filtered occurrences were assigned to 0.25° grid cells by applying the floor function to latitude and longitude divided by 0.25. The resulting 5853 occurrences represented 2281 unique spatial blocks. Taxon-specific attrition, final occurrence counts and environmental-model coverage are reported in Supplementary Table S1.

### 4.7. SoilGrids Data Collection and Soil-Niche Feature Engineering

Candidate taxon × block rows were stratified using eight latitudinal bands (−90 to −60°, −60 to −35°, −35 to −23.5°, −23.5 to 0°, and 0 to 23.5°, 23.5 to 35°, 35 to 60°, and 60 to 90°) crossed with 30° longitude sectors. For each taxon, the highest-priority block within each occupied geographic stratum was selected first; remaining blocks were then ranked by cached-data availability and the number of occurrences represented, with a seeded random value used only to break ties. The target was 60 initially selected soil blocks per taxon.

An actual occurrence coordinate was used as the environmental query point. Within each spatial block, records were ordered by coordinate uncertainty, distance to the block centroid and GBIF key, and the highest-ranked occurrence was selected. This avoided querying a geometric cell centroid that could fall in water or outside mapped soil coverage. A spatial block selected for one taxon was queried only once, and the retrieved values were joined back to all taxa represented in that block. Consequently, the number of complete blocks available to an individual taxon after joining could differ from the initial selection target.

Soil predictions were retrieved from SoilGrids 2.0 [14] for the following seven properties: pH in water (phh2o), soil organic carbon (soc), clay, sand, silt, cation-exchange capacity (cec) and total nitrogen. Mean predictions were retrieved for 0–5, 5–15 and 15–30 cm depths and divided by the property-specific SoilGrids scale factors, producing 21 soil features. Failed requests and responses without usable values were logged. Only rows with complete 21-feature vectors were used in the primary analysis.

Environmental values were first joined to the occurrence records by spatial-block identifier. Records were then aggregated to one row per taxon × spatial block; soil values were averaged, although occurrences in the same query block shared the same extracted vector. The final soil data comprised 1563 SoilGrids-linked occurrences aggregated to 248 complete taxon × spatial-block rows. Taxon-specific coverage ranged from 33 to 62 complete soil blocks. Occurrence-level rows were retained only for sensitivity analyses.

### 4.8. NASA POWER Climate Data Collection and Climate-Niche Feature Engineering

Climate variables were obtained from the NASA POWER climatology point API [15]. The same 0.25° geographic stratification and representative-occurrence procedure used for SoilGrids was applied, but the initial climate selection target was 40 blocks per taxon. Previously cached JSON responses were reused, shared blocks were queried only once and climate values were joined back to all represented taxa.

Seven monthly climatology parameters were retrieved: mean 2 m air temperature (T2M), maximum 2 m air temperature (T2M_MAX), minimum 2 m air temperature (T2M_MIN), corrected precipitation (PRECTOTCORR), 2 m relative humidity (RH2M), all-sky surface shortwave radiation (ALLSKY_SFC_SW_DWN) and 2 m wind speed (WS2M). Monthly values were transformed into 62 climate-niche features. These included annual temperature summaries, temperature seasonality and range, warmest- and coldest-month extremes, annual and quarterly precipitation summaries, precipitation seasonality, warmest- and coldest-quarter temperatures, annual and seasonal humidity, radiation and wind summaries, and monthly temperature, precipitation-rate and relative-humidity profiles.

NASA POWER climatological PRECTOTCORR values were treated as monthly mean daily rates in mm per day. Monthly precipitation-rate values were retained for the monthly profile variables. For approximate monthly totals, the rate was multiplied by the number of days in the corresponding month, using 28.25 days for February; annual and quarterly precipitation totals were derived from these approximate monthly totals.

Climate features were joined to occurrences by a spatial-block identifier and aggregated to one taxon × block row. The final climate data comprised 2274 climate-linked occurrences aggregated to 324 complete taxon × spatial-block rows. The taxon-specific coverage ranged from 42 to 71 complete climate blocks. No missing climate-feature values remained in the primary block table.

### 4.9. Construction and Preprocessing of Analytical Feature Tables

Taxon-level genetic-resource and pathway tables were joined by taxon_id. The complete evidence inventory was constructed as one row per taxon_id × compound_id. Record type was then assigned from the compound dictionary, yielding the 36-row specific-molecule primary table and the 24-row broad-class descriptive table. Occurrence information was summarized by unique GBIF records and countries and by latitude, longitude and coordinate-uncertainty statistics. The fixed input-taxonomy label, rather than occurrence-level scientific-name strings, was used for aggregation to prevent genus-level taxa from being fragmented into multiple species-level rows.

The environmental occurrence tables were not crossed with compound annotations for the primary niche analyses. Compound expansion was performed only to construct the prespecified soil-only and climate-only negative controls. The analytical unit of the primary environmental models remained taxon × spatial block.

Predictor selection was not based on univariate association with the response. Predictive preprocessing was performed independently within every training fold. Missing numeric values were imputed using the median estimated from the training fold. Logistic and ridge models were additionally standardized using training-fold means and standard deviations. Tree-based models used median imputation but no scaling. Descriptive PCA used full-matrix median imputation followed by standardization to the zero mean and unit variance; the PCA transformation was not used for predictive evaluation. Taxon-level profile heatmaps were standardized across taxa only for visualization.

### 4.10. Descriptive and Multivariate Statistical Analyses

Coverage heatmaps summarized public-data availability by taxon, including genetic records, pathway evidence, soil-linked cells, climate-linked cells and compound-evidence annotations. Spearman rank correlation was used to examine the association between the ordinal evidence score and LOTO out-of-fold probabilities within the 36 specific-molecule rows. The complete ten-record inventory was additionally summarized as six individual molecules and four broad compound classes. These comparisons were interpreted as audits of model behavior rather than independent biological validation.

For the PCA, numeric variables were median-imputed and standardized before decomposition. The soil and climate PCAs used taxon × spatial-block rows. For each taxon with sufficient observations, a 95% covariance ellipse was calculated in the PC1–PC2 plane from the taxon-specific covariance matrix and the 0.95 quantile of the chi-squared distribution with two degrees of freedom. These ellipses summarize within-taxon dispersion and are not confidence regions for the taxon centroids.

Differences among taxa in the complete standardized soil and climate feature spaces were evaluated using Euclidean-distance PERMANOVA with 999 taxon-label permutations restricted within 1° spatial clusters [41]. Multivariate dispersion was evaluated separately from distances to taxon centroids, also using 999 permutations restricted within the same 1° clusters [42]. These restrictions preserved coarse geographic exchangeability more appropriately than unrestricted permutations. Pairwise PERMANOVA summaries, when reported, used the same restriction and Benjamini–Hochberg adjustment [43]. PERMANOVA results were interpreted together with the corresponding dispersion test and the grouped-classification sensitivity analyses.

Sensitivity to unequal taxon sample sizes was evaluated by repeated balanced block subsampling. In each of 100 repetitions, the same number of taxon × cell rows was sampled without replacement from every taxon, as follows: 33 per taxon for soil and 42 per taxon for climate. Each balanced dataset was re-analyzed with 99-permutation PERMANOVA and dispersion tests using the same 1° exchangeability restriction. A common auxiliary multinomial logistic classifier was evaluated under exact-cell grouped validation in both branches to provide a model-family-consistent diagnostic; it was distinct from the prespecified primary soil random forest. Distributions of PERMANOVA R^2^, fractions of significant multivariate tests and auxiliary grouped-classification balanced accuracy were retained as sensitivity summaries (Supplementary Table S6A).

### 4.11. Machine Learning Models, Leakage Audit and Validation Design

Machine learning analyses were implemented in scikit-learn [44]. Logistic regression, random forest and gradient boosting were evaluated in all three branches; histogram gradient boosting was additionally evaluated in the genetic/pathway branch. Dummy most-frequent classifiers were used as chance baselines. All preprocessing and model fitting occurred inside the corresponding cross-validation fold.

#### 4.11.1. Genetic/Pathway Predictor Audit and Primary Model

Numeric variables were eligible for the genetic/pathway audit when they contained at least three numeric non-missing observations and more than one unique value. Variables were then assigned to one of five mutually exclusive analytical categories, as follows: retained predictor, target-construction leakage, identifier or non-predictive metadata, zero-variance variable, or low-specificity descriptive evidence.

The binary target and ordinal evidence score were excluded by definition. Variables used directly or indirectly to construct the response—including PubMed record counts, literature-support indicators, exact compound-title matches and curated-positive indicators—were excluded as target-construction leakage. Taxon, compound, row and grouping identifiers, names, file paths and accession metadata were excluded as non-predictive metadata. The numeric PubChem identifier pubchem_cid was explicitly treated as an identifier and excluded. Constant variables were removed without reference to the response. Five generic broad enzyme-family hit variables were retained for descriptive summaries but excluded from the classifier because they were insufficiently pathway-specific.

Different validation schemes were used because they address different generalization questions. Conventional stratified random-row cross-validation estimates interpolation among observations sampled from the same dataset, but it can be optimistic when multiple rows share taxon-level predictors, pathway annotations or other hierarchical dependencies. In the present taxon–compound table, six molecule rows belong to each taxon and several genetic-resource variables are repeated across those rows. Random row splitting could, therefore, place observations carrying the same taxon-level information in the both training and test sets. Cross-validation strategies that respect spatial, hierarchical or phylogenetic structure are recommended when such dependencies are present [20], and data leakage has been shown to compromise ecological machine learning assessments when related observations are divided between training and testing [22]. The complete 60-row inventory was audited for target-construction leakage, identifiers, low-specificity broad-family variables and record-type artefacts. Because training-fold median imputation would allow molecular weight to encode record type, broad classes were excluded from primary predictive modeling. The four pathway variables were constructed through a prespecified compound-to-pathway mapping and, thus, represented explicit taxon–compound interactions. Generic broad-family counts, target-construction variables, identifiers and record-type indicators were excluded.

LOTO was consequently selected as the primary design because it tests whether the complete evidence profile learned from five taxa transfers to a sixth, previously unseen taxon. This is conceptually related to cross-species transfer evaluation in specialized-metabolism gene prediction [7,8], although the analytical unit and outcome are different in the present study. LOCO was retained as a secondary diagnostic because it tests transfer to a previously unseen molecule rather than to an unseen taxon. It was not treated as a substitute for LOTO because taxon and molecule holdouts evaluate different forms of extrapolation, and the molecule-only dataset contains only six compounds. For environmental analyses, GroupKFold by exact 0.25° cell prevented identical cells from occurring in both the training and test sets. Because neighboring cells may, nevertheless, remain spatially autocorrelated, validation was repeated with coarser 1° groups, consistent with recommendations for spatially structured ecological data and evidence that non-spatial validation can overestimate geographic transferability [20,21]. Random-row evaluation was retained only as a non-grouped diagnostic and was not used as evidence of external generalization.

Task-matched benchmarks used the same 36 molecule rows and LOTO folds. Random forest models were fitted to the four matched-pathway variables alone, molecular weight alone, a coverage-control block and the wider leakage-screened numeric set. Balanced logistic regression and gradient boosting using the five-variable core were model-family comparators; the logistic result was post hoc and did not replace the prespecified primary test. A separate fold-eligibility audit applied LOCCO to the complete 60-row mixed-record inventory. Of ten compound classes, only eight produced test folds containing both labels; flavonoid- and phenolic-compound folds were excluded from the balanced-accuracy calculation.

#### 4.11.2. Environmental Taxon-Niche Models

The response in the primary soil and climate models was a six-class taxon identity. The soil models used 21 SoilGrids features, and the climate models used 62 NASA POWER-derived features. Primary five-fold GroupKFold validation grouped the rows by the exact 0.25° spatial_block_id, so the same cell could not occur in both the training and test data [20,21,22]. The soil table contained 248 taxon-cell rows across 188 unique exact cells; the climate table contained 324 rows across 226 exact cells. Because adjacent cells could still be assigned to different folds, a prespecified sensitivity analysis repeated grouped validation using coarser 1° clusters (153 soil and 179 climate clusters). The primary design is, therefore, described as exact-cell grouped validation rather than geographically separated spatial cross-validation.

The soil random forest and climate class-weighted multinomial logistic regression models were prespecified as the primary taxon-niche models before evaluation. Logistic regression used training-fold median imputation, standardization, a maximum of 3000 iterations and balanced class weights. The random forest used training-fold median imputation, 500 trees, a minimum leaf size of two, balanced class weights and a seed of 42. Dummy, alternative logistic, random forest and gradient-boosting models were retained as secondary diagnostics; primary-model selection was not repeated after inspecting their cross-validation performance. Label-permutation tests and the 1° sensitivity analyses used the same prespecified model for each environmental branch.

#### 4.11.3. Performance Measures and Uncertainty Intervals

Balanced accuracy was the primary performance metric because the genetic binary target and the environmental taxon classes were not perfectly balanced. Accuracy, precision, recall, F1-score, Matthews correlation coefficient, ROC-AUC and confusion matrices were reported as complementary measures. The average precision was additionally reported for the binary genetic and negative-control tasks. Multiclass precision, recall and F1 values were macro-averaged; the multiclass ROC-AUC used a one-versus-rest macro average. Binary class assignments used the default probability threshold of 0.5. Performance metrics were calculated from out-of-fold predictions only.

For grouped genetic analyses, the reported 95% fold interval was the empirical 2.5th–97.5th percentile of the valid fold-level metric values and was not interpreted as a confidence interval for the population mean. For the five-fold environmental taxon classifiers and the environmental binary controls, 95% intervals around the mean used the Student t distribution across valid grouped folds. The interval definition and number of valid folds for every analysis were retained in the exported source tables.

#### 4.11.4. Label-Permutation and Positive–Unlabeled Sensitivity Analyses

For the primary genetic model, 200 global label permutations were generated while retaining the prespecified predictor matrix, model and LOTO grouping structure. The empirical one-sided *p*-value was calculated as (1 + number of permuted balanced accuracies greater than or equal to the observed value)/(1 + number of permutations).

Because scores of 0–1 represented unlabeled background rather than confirmed absence, robustness to background construction was evaluated as a positive–unlabeled sensitivity analysis [23]. All eight supported molecule rows were retained, and rows were sampled from the 28 molecule-level background pairs at ratios of 0.25, 0.50 and 1.00 relative to the number of supported rows. Four strategies were evaluated: global random, taxon-stratified, compound-class-stratified and simultaneous taxon/compound-class stratification. Each feasible ratio–strategy combination was repeated 30 times using stable original row order and the same unweighted mean of valid LOTO fold metrics as the primary analysis. Some reduced samples produced held-out taxa with a single label class, so only two to six folds were valid; the number of valid folds was reported for each design. The deterministic full-background analysis used all 28 background rows and all six LOTO folds.

For the soil and climate taxon-niche classifiers, 200 taxon-label permutations tested whether exact-cell grouped balanced accuracy exceeded the null. The exact grouping and the prespecified branch-specific model were fixed in every permutation, and empirical one-sided *p*-values used the plus-one correction. Separate PERMANOVA and dispersion analyses restricted label exchange within the 1° spatial clusters, as described in Section 4.10.

#### 4.11.5. Environmental Compound Negative Controls

Environmental compound models were used only as negative controls. Soil or climate features were used to classify the 48 taxon–compound annotation rows under LOTO and leave-one-compound-class-out validation. The evaluated models were a dummy most-frequent classifier, class-weighted L2 logistic regression and random forest. These analyses tested whether environmental features could spuriously appear to encode compound annotations; they were not treated as metabolite-prediction models.

Each non-dummy environmental negative-control analysis used 200 permutations. Labels were permuted separately within the validation-group structure so that the class composition, held-out groups and number of valid folds were preserved. The observed mean balanced accuracy was compared with the corresponding restricted-permutation null distribution. A non-significant result was interpreted as failure to support compound-level inference from environmental features, not as evidence that a compound was absent.

### 4.12. Model Interpretation and Prioritization Framework

For the environmental taxon models, feature importance was interpreted only as indicating which soil or climate variables contributed to differentiation among the six taxon-associated niches. Environmental importance was not converted into compound evidence and was not interpreted as a causal effect on metabolite accumulation.

Taxon–compound priorities were based on LOTO out-of-fold probabilities from the prespecified genetic model. The probabilities were interpreted as relative evidence-prioritization scores rather than probabilities of metabolite presence. Candidate selection for experimental follow-up considered the following four separate dimensions: (i) independently curated or literature-supported taxon–compound evidence; (ii) pathway-specific candidate-gene support; (iii) LOTO out-of-fold prioritization score; and (iv) practical feasibility indicated by available genomic, transcriptomic and occurrence resources. Environmental niche results were reported separately as context for designing representative sampling across sites and conditions. They did not increase or decrease the compound-evidence score.

No model-derived “benchmark”, “strong integrated” or “exploratory” taxon classes were assigned. The six prespecified benchmark associations served only as a biological recovery audit, whereas other taxon–compound rows were retained as ranked hypotheses with explicit uncertainty.

### 4.13. Limitations, Inference Boundary and Data Availability

GBIF occurrences are presence-only observations and may reflect collection intensity, geographic accessibility and taxonomic interest. SoilGrids and NASA POWER values are gridded model-derived estimates rather than field measurements of rhizosphere soil or plant-level microclimate. Public genetic and pathway records are also unevenly distributed among taxa. Consequently, data-rich taxa may receive stronger apparent support because they have been studied more extensively.

The 33 unlabeled taxon–compound rows were not confirmed biochemical absences. Likewise, the environmental analyses detected taxon-associated classification signals and descriptive environmental structures, but the spatially restricted multivariate tests did not establish significant global centroid separation. These analyses did not demonstrate compound identity, metabolite concentration, habitat suitability or causal soil and climate effects. Direct accumulation-level inference will require independent quantitative metabolomic or targeted phytochemical measurements collected from harmonized tissues, developmental stages, seasons and sites and reported with appropriate analytical metadata [35]. Transcriptomic, biochemical or molecular assays will additionally be needed to validate prioritized pathway candidates.

The Supplementary Data Archive contains the executed notebook set, frozen inputs, cached intermediates, validation-group tables, model summaries, figure-source tables and available configuration, manifest and software-environment records. Configuration files are present for the final genetic and soil modeling runs; the climate parameters are documented in the executed notebook and manifests. The frozen inputs permit the reported modeling analyses to be rerun without live API access. The archive should be deposited as a versioned, publicly accessible release together with the accepted manuscript.

## 5. Conclusions

This study presents a reproducible, leakage-aware workflow for auditing heterogeneous public evidence relevant to medicinal-plant taxon–compound hypotheses. Separation of specific-molecule and broad-class records removed a major molecular-weight missingness artefact and clarified the contribution of pathway-group-matched evidence. However, the prespecified molecule-level model did not outperform its permutation null, and its out-of-fold rankings recovered established associations inconsistently while assigning several of the highest scores to unlabeled-background pairs. The model should, therefore, not be used as a standalone predictor of metabolite presence or as a validated candidate-ranking system. Soil and climate models detected the taxon-associated classification signal under grouped validation, but spatially restricted multivariate tests were non-significant and did not establish ecological separation or environment–metabolite relationships. The principal contribution of the framework is thus methodological: it exposes leakage and coverage artefacts, separates non-equivalent analytical units, defines defensible inference boundaries and generates explicitly testable hypotheses for prospective metabolomic, transcriptomic and biochemical validation.

## Figures and Tables

**Table 1:** Core datasets, analytical units, outcomes and validation designs in the revised framework.

Analysis Branch	Primary Unit	Rows/Class Balance	Outcome	Predictors	Validation	Supported Role
Complete genetic/pathway inventory	Taxon–compound record	60 (6 taxa × 10 records); 27 supported; 33 below threshold (19 score-0, 14 score-1)	Binary support label plus 0–3 descriptive evidence score	Integrated open-data evidence inventory	Record-type and leakage audits before modeling	Defines the evidence universe; does not establish biochemical absence
Specific-molecule primary model	Unique taxon–molecule pair	36 (6 × 6); 8 supported, 28 background	Binary open-data support label	Five prespecified variables: four compound-matched pathway features plus molecular weight	Primary LOTO; secondary LOCO/LOCCO; 200 label permutations	Exploratory transfer-aware ranking for experimental prioritization
Broad-class descriptive analysis	Taxon–broad-class pair	24 (6 × 4); 19 supported, 5 background	Observed evidence score/support label	Descriptive evidence and pathway coverage	No primary predictive model	Separates broad annotations from specific-molecule inference
Soil-niche characterization	Taxon × exact 0.25° cell	248 taxon-cell rows; 188 unique exact cells; 153 1° clusters	Six-class taxon identity	21 SoilGrids-derived features	Exact-cell grouped 5-fold CV; 1° sensitivity; restricted permutations within 1° clusters	Taxon-associated edaphic context, not compound prediction
Climate-niche characterization	Taxon × exact 0.25° cell	324 taxon-cell rows; 226 unique exact cells; 179 1° clusters	Six-class taxon identity	62 NASA POWER-derived features	Exact-cell grouped 5-fold CV; 1° sensitivity; restricted permutations within 1° clusters	Taxon-associated climatic context, not compound prediction
Soil-only compound control	Compound-expanded taxon row	48 (6 taxa × 8 shared annotations); 10 supported, 38 background	Binary open-data support label	21 soil features; constant within taxon	Prespecified random forest; LOTO and 8-fold LOCCO	Tests and rejects direct soil-to-compound inference
Climate-only compound control	Compound-expanded taxon row	48 (6 taxa × 8 shared annotations); 10 supported, 38 background	Binary open-data support label	62 climate features; constant within taxon	Prespecified balanced logistic regression; LOTO and 8-fold LOCCO	Tests and rejects direct climate-to-compound inference

Note. LOTO, leave-one-taxon-out; LOCO, leave-one-compound-out; LOCCO, leave-one-compound-class-out. Unlabeled-background rows indicate no supported association under the current open-data criteria and are not confirmed biochemical absences. All predictive preprocessing was fitted within the training folds.

**Table 2:** Primary grouped-validation, permutation-test and environmental inferential results.

Analysis	Unit/Validation	Prespecified Method	Folds	Primary Result	Secondary Metrics/Tests	Interpretation
Specific-molecule evidence	36 taxon–molecule pairs/LOTO	Balanced random forest; five-variable matched core	6	BA 0.621 [0.500–0.800] (fold interval)	Acc: 0.694; precision: 0.250; recall: 0.500; F1: 0.317; MCC: 0.191; ROC-AUC: 0.425; AP: 0.361; Perm.: *p* = 0.154.	Performance was not distinguishable from the label-permutation null; use scores only for exploratory ranking
Logistic comparator	36 taxon–molecule pairs/LOTO	Balanced logistic regression; same five variables	6	BA 0.646 [0.500–0.794] (fold interval)	Acc: 0.667; precision: 0.247; recall: 0.667; F1: 0.356; MCC: 0.263; ROC-AUC: 0.733; AP: 0.624; Perm.: *p* —.	Post hoc comparator; slightly higher mean BA than the prespecified random forest, but not the primary test
Matched-pathway-only benchmark	36 taxon–molecule pairs/LOTO	Balanced random forest; four matched-pathway variables	6	BA 0.662 [0.500–0.800] (fold interval)	Acc: 0.694; precision: 0.261; recall: 0.667; F1 0.373; MCC: 0.285; ROC-AUC: 0.662; AP: 0.444; Perm.: *p* —.	Molecular weight was not responsible for the signal in the molecule-only analysis
Cross-molecule transfer	36 taxon–molecule pairs/LOCO (=LOCCO here)	Balanced random forest; five-variable matched core	6	BA 0.746 [0.516–0.975] (fold interval)	Acc: 0.778; precision: 0.528; recall: 0.667; F1: 0.528; MCC: 0.463; ROC-AUC: 0.794; AP: 0.686; Perm.: *p* —.	Wide fold interval; each molecule has a unique class in the molecule-only set
Mixed-record LOCCO eligibility audit	60 inventory rows/10 compound classes	Fold-eligibility audit only	8 valid of 10	48 of 60 rows evaluated	Flavonoid- and phenolic-compound folds excluded because each held-out set contained one label class.	Do not describe this audit as ten valid performance folds
Soil taxon-niche classification	248 taxon-cell rows/exact-cell grouped CV	Random forest	5	BA 0.479 [0.422–0.537] (t interval)	Acc: 0.512; macro precision: 0.443; macro recall: 0.479; macro F1: 0.437; MCC: 0.417; ROC-AUC: 0.812; restricted PERMANOVA: *p* = 0.067; dispersion: *p* = 0.266; Perm.: *p* —.	Taxon classification exceeded its label null; coarse 1° sensitivity, BA: 0.492 [0.427–0.557]
Climate taxon-niche classification	324 taxon-cell rows/exact-cell grouped CV	Multinomial logistic regression	5	BA 0.547 [0.483–0.612] (t interval)	Acc: 0.515; macro precision: 0.539; macro recall: 0.547; macro F1: 0.510; MCC: 0.433; ROC-AUC: 0.815; restricted PERMANOVA: *p* = 0.075; dispersion: *p* = 0.574; Perm.: *p* = 0.005.	Taxon classification exceeded its label null; coarse 1° sensitivity, BA: 0.551 [0.498–0.604]
Soil-only compound control	48 rows/LOTO	Random forest	6	BA 0.500 [0.500–0.500] (t interval)	F1: 0.037; Perm. P 1.000.	No compound-level support from soil
Soil-only compound control	48 rows/LOCCO	Random forest	8	BA 0.406 [0.206–0.606] (t interval)	F1: 0.188; Perm.: *p* = 0.736.	No compound-level support from soil
Climate-only compound control	48 rows/LOTO	Balanced logistic regression	6	BA 0.500 [0.500–0.500] (t interval)	F1: 0.232; Perm.: *p* = 1.000.	No compound-level support from climate
Climate-only compound control	48 rows/LOCCO	Balanced logistic regression	8	BA 0.406 [0.206–0.606] (t interval)	F1: 0.188; Perm.: *p* = 0.692.	No compound-level support from climate

Note. BA, balanced accuracy; AP, average precision; MCC, Matthews correlation coefficient; LOTO, leave-one-taxon-out; LOCO, leave-one-compound-out; LOCCO, leave-one-compound-class-out. Genetic intervals are empirical 95% fold-performance intervals, not confidence intervals, for a population mean. Environmental intervals are t-based 95% intervals across valid grouped folds. The six-class chance reference for taxon-niche models is 0.167; the binary chance reference is 0.500. Restricted PERMANOVA and dispersion *p*-values were obtained by permuting labels within 1° spatial clusters.

**Table 3:** Comparison of the present framework with representative computational approaches relevant to plant specialized metabolism and medicinal-plant research.

Approach	Analytical Unit	Required Data	Primary Output	Principal Scope Limitation	Relationship to the Present Framework	Refs.
Specialized-metabolism gene prediction	Gene	Genome and multi-omics features	Candidate specialized-metabolism genes	Requires sufficiently informative genomic or multi-omics resources; does not directly rank taxon–compound pairs	Provides gene-level evidence that can inform the pathway-support layer	[7,8]
Plant biosynthetic-gene-cluster discovery	Genomic region/gene cluster	Annotated plant genomes; optional expression data	Candidate biosynthetic gene clusters	Depends on genome completeness and detectable clustering; output is a genomic region, not compound occurrence	Complements the pathway layer when adequate genomes are available	[9]
Molecular pathway classification	Molecule	Molecular graph or SMILES representation	Predicted secondary-metabolic pathway class	Classifies molecular structures but does not identify the producing taxon	Addresses a compound-centered task distinct from cross-taxon evidence prioritization	[10]
Bio-retrosynthetic pathway navigation	Target molecule and reaction network	Target structure and reaction data	Candidate precursors and biosynthetic routes	Evaluates route plausibility without establishing taxon-specific evidence or metabolite abundance	Can generate pathway hypotheses for prioritized compounds	[11]
Natural-product knowledge management	Referenced structure–organism association	Literature and database records	Harmonized reported associations	Subject to research and reporting coverage; an unreported association is not a biochemical absence	Supplies curated association evidence while motivating positive–unlabeled treatment	[12]
Habitat or cultivation-suitability modeling	Location or spatial grid cell	Occurrence, soil, climate and terrain data	Habitat or cultivation suitability	Does not establish compound identity, pathway support or metabolite concentration	Corresponds to an ecological task kept separate from compound evidence	[16]
Ethnobotanical prioritization	Taxon and use report	Field-use reports and ethnobotanical indices	Cultural or use-based priority	Does not independently establish chemical composition, efficacy or biosynthetic capacity	Offers complementary use-based context	[17]
Ecological–phytochemical quality modeling	Measured population or site	Measured constituents plus environmental predictors	Spatial prediction of medicinal quality	Requires harmonized quantitative chemical outcomes and matched sampling metadata	Represents a future extension once comparable metabolomic data are available	[18]
Present leakage-aware framework	Taxon–compound pair; taxon × spatial block	Open genetic, pathway, occurrence, soil, climate and compound evidence	Evidence-weighted priorities, ecological niche context and inference boundaries	Does not confirm metabolite presence, absence or concentration; performance is task- and data-coverage-dependent	Integrates complementary evidence while separating analytical units, validation schemes and supported inferences	This study

Note. The approaches address non-equivalent analytical units and outcomes; therefore, direct numerical comparison of reported accuracies would be misleading. SMILES, simplified molecular-input line-entry system; Refs., reference numbers in the revised manuscript.

## Data Availability

The analysis code is available in the Supplementary Materials and at the repository https://github.com/samarinalidia-design/medicinal-plant-open-data-framework, accessed on 26 July 2026.

## Supplementary Materials

The following supporting information can be downloaded at: https://www.mdpi.com/article/10.3390/ijms27156801/s1.
