# Supplementary material for: Integrating Biosynthetic, Genomic and Ecological Open Data for Medicinal Plant Research: A Leakage-Aware Evidence-Prioritization Framework
Source: Int J Mol Sci. 2026 Jul 29;27(15):6801. doi: 10.3390/ijms27156801 (PMC13467606; doi:10.3390/ijms27156801)
Supplement: Supplementary file 1 [file ijms-27-06801-s001.zip › med plant data revised notebooks/Supplementary_tables_revised_data_aligned.pdf]

Table S1. GBIF filtering and environmental coverage

Table S1A. Liberal-filter attrition by taxon

Source: soilgrids_notebook1_outputs/gbif/gbif_filter_attrition_by_taxon.tsv; liberal filter, uncertainty ≤10,000 m or missing.

Taxon	Entering	Missing coord.	Out of range	Zero coord.	Serious GBIF issue	>10 km uncertainty	Missing uncertainty retained	Retained
Artemisia annua	1000	0	0	0	0	17	271	983
Glycyrrhiza glabra	1000	0	0	0	3	15	455	982
Hypericum	1000	0	0	0	0	43	359	957
Rhodiola	1000	0	0	0	0	26	209	974
Sedum	1000	0	0	0	0	31	223	969
Taxus	1000	0	0	0	0	12	85	988

Table S1B. Linked occurrences and final spatial units

Counts correspond to the frozen primary environmental inputs used by the modelling notebooks.

Taxon	Soil-linked occurrences	Soil taxon × exact-cell rows	Climate-linked occurrences	Climate taxon × exact-cell rows
Artemisia annua	213	33	415	50
Glycyrrhiza glabra	352	45	395	43
Hypericum	272	62	299	71
Rhodiola	260	39	316	42
Sedum	149	36	349	66
Taxus	317	33	500	52
Total	1563	248	2274	324

Table S2. Record-type and feature-leakage audits

Table S2A. Record-type target and molecular-weight audit

Source: notebook3_v3_reviewer_corrected_outputs/derived/record_type_target_molecular_weight_audit.tsv.

Record type	Rows	Compounds	Supported	MW missing	Supported fraction
broad_compound_class	24	4	19	24	0.792
specific_molecule	36	6	8	0	0.222

Table S2B. Current molecule-only feature-leakage audit

Source: notebook3_v3_reviewer_corrected_outputs/derived/feature_leakage_audit_v3.tsv (36 specific-molecule rows; 96 audited columns).

Column	Status	Reason	Non-missing	Missing	Numeric unique	Feature block
compound_class	excluded_insufficient_numeric_data	fewer than three numeric observations	36	0	0	—
open_data_ml_readiness	excluded_insufficient_numeric_data	fewer than three numeric observations	36	0	0	—
PWY_ALKALOID__n_broad_family_hits	excluded_low_specificity_descriptive_evidence	generic broad enzyme-family evidence; retained only for descriptive summaries	36	0	6	—
PWY_HYPERICIN__n_broad_family_hits	excluded_low_specificity_descriptive_evidence	generic broad enzyme-family evidence; retained only for descriptive summaries	36	0	6	—
PWY_PHENYLPROPANOID_FLAVONOID__n_broad_family_hits	excluded_low_specificity_descriptive_evidence	generic broad enzyme-family evidence; retained only for descriptive summaries	36	0	6	—
PWY_SALIDROSIDE__n_broad_family_hits	excluded_low_specificity_descriptive_evidence	generic broad enzyme-family evidence; retained only for descriptive summaries	36	0	5	—
PWY_TERPENOID__n_broad_family_hits	excluded_low_specificity_descriptive_evidence	generic broad enzyme-family evidence; retained only for descriptive summaries	36	0	6	—
analysis_record_type	excluded_metadata_or_identifier	identifier, categorical metadata, provenance, or grouping variable	36	0	0	—
compound_dictionary_status	excluded_metadata_or_identifier	identifier, categorical metadata, provenance, or grouping variable	36	0	0	—
compound_id	excluded_metadata_or_identifier	identifier, categorical metadata, provenance, or grouping variable	36	0	0	—
compound_name	excluded_metadata_or_identifier	identifier, categorical metadata, provenance, or grouping variable	36	0	0	—
compound_record_type	excluded_metadata_or_identifier	identifier, categorical metadata, provenance, or grouping variable	36	0	0	—
family	excluded_metadata_or_identifier	identifier, categorical metadata, provenance, or grouping variable	36	0	0	—
inchikey	excluded_metadata_or_identifier	identifier, categorical metadata, provenance, or grouping variable	36	0	0	—
is_broad_compound_class	excluded_metadata_or_identifier	identifier, categorical metadata, provenance, or grouping variable	36	0	1	—
is_specific_molecule	excluded_metadata_or_identifier	identifier, categorical metadata, provenance, or grouping variable	36	0	1	—
mapping_source	excluded_metadata_or_identifier	identifier, categorical metadata, provenance, or grouping variable	36	0	0	—
matched_pathway_id	excluded_metadata_or_identifier	identifier, categorical metadata, provenance, or grouping variable	36	0	0	—
ml_role	excluded_metadata_or_identifier	identifier, categorical metadata, provenance, or grouping variable	36	0	0	—
molecular_formula	excluded_metadata_or_identifier	identifier, categorical metadata, provenance, or grouping variable	36	0	0	—
molecular_weight_missing_flag	excluded_metadata_or_identifier	identifier, categorical metadata, provenance, or grouping variable	36	0	1	—
notebook3_primary_row_id	excluded_metadata_or_identifier	identifier, categorical metadata, provenance, or grouping variable	36	0	0	—
priority	excluded_metadata_or_identifier	identifier, categorical metadata, provenance, or grouping variable	36	0	0	—
pubchem_cid	excluded_metadata_or_identifier	identifier, categorical metadata, provenance, or grouping variable	36	0	6	—
rank	excluded_metadata_or_identifier	identifier, categorical metadata, provenance, or grouping variable	36	0	0	—
record_type	excluded_metadata_or_identifier	identifier, categorical metadata, provenance, or grouping variable	36	0	0	—
scientific_name	excluded_metadata_or_identifier	identifier, categorical metadata, provenance, or grouping variable	36	0	0	—
taxon_id	excluded_metadata_or_identifier	identifier, categorical metadata, provenance, or grouping variable	36	0	0	—
target_evidence_score	excluded_target	response variable	36	0	4	—
target_label_for_open_data_classifier	excluded_target	response variable	36	0	2	—
has_accumulation_or_pathway_terms	excluded_target_construction_leakage	directly or indirectly used to construct the response	36	0	2	—
has_exact_compound_in_title	excluded_target_construction_leakage	directly or indirectly used to construct the response	36	0	2	—
has_literature_support	excluded_target_construction_leakage	directly or indirectly used to construct the response	36	0	2	—
is_curated_positive_pair	excluded_target_construction_leakage	directly or indirectly used to construct the response	36	0	2	—
n_recent_pubmed_records_2015plus	excluded_target_construction_leakage	directly or indirectly used to construct the response	36	0	7	—
n_supporting_pubmed_records	excluded_target_construction_leakage	directly or indirectly used to construct the response	36	0	6	—
pseudo_negative_flag	excluded_target_construction_leakage	directly or indirectly used to construct the response	36	0	2	—

Column	Status	Reason	Non-missing	Missing	Numeric unique	Feature block
target_confidence_initial	excluded_target_construction_leakage	directly or indirectly used to construct the response	36	0	0	—
target_evidence_class	excluded_target_construction_leakage	directly or indirectly used to construct the response	36	0	0	—
target_type_recommendation	excluded_target_construction_leakage	directly or indirectly used to construct the response	36	0	0	—
target_warning	excluded_target_construction_leakage	directly or indirectly used to construct the response	36	0	0	—
PWY_HYPERICIN__n_specific_candidate_hits	excluded_zero_variance	constant numeric variable	36	0	1	—
genetic_data_availability_score	excluded_zero_variance	constant numeric variable	36	0	1	—
PWY_ALKALOID__max_specificity_score	included_safe_predictor	numeric, variable, prespecified as non-leaky and non-identifier	36	0	4	pathway_biosynthetic
PWY_ALKALOID__n_candidate_protein_hits	included_safe_predictor	numeric, variable, prespecified as non-leaky and non-identifier	36	0	6	pathway_biosynthetic
PWY_ALKALOID__n_specific_candidate_hits	included_safe_predictor	numeric, variable, prespecified as non-leaky and non-identifier	36	0	4	pathway_biosynthetic
PWY_ALKALOID__pathway_potential_score	included_safe_predictor	numeric, variable, prespecified as non-leaky and non-identifier	36	0	6	pathway_biosynthetic
PWY_HYPERICIN__max_specificity_score	included_safe_predictor	numeric, variable, prespecified as non-leaky and non-identifier	36	0	2	pathway_biosynthetic
PWY_HYPERICIN__n_candidate_protein_hits	included_safe_predictor	numeric, variable, prespecified as non-leaky and non-identifier	36	0	6	pathway_biosynthetic
PWY_HYPERICIN__pathway_potential_score	included_safe_predictor	numeric, variable, prespecified as non-leaky and non-identifier	36	0	3	pathway_biosynthetic
PWY_PHENYLPROPANOID_FLAVONOID__max_specificity_score	included_safe_predictor	numeric, variable, prespecified as non-leaky and non-identifier	36	0	2	pathway_biosynthetic
PWY_PHENYLPROPANOID_FLAVONOID__n_candidate_protein_hits	included_safe_predictor	numeric, variable, prespecified as non-leaky and non-identifier	36	0	5	pathway_biosynthetic
PWY_PHENYLPROPANOID_FLAVONOID__n_specific_candidate_hits	included_safe_predictor	numeric, variable, prespecified as non-leaky and non-identifier	36	0	6	pathway_biosynthetic
PWY_PHENYLPROPANOID_FLAVONOID__pathway_potential_score	included_safe_predictor	numeric, variable, prespecified as non-leaky and non-identifier	36	0	5	pathway_biosynthetic
PWY_SALIDROSIDE__max_specificity_score	included_safe_predictor	numeric, variable, prespecified as non-leaky and non-identifier	36	0	3	pathway_biosynthetic
PWY_SALIDROSIDE__n_candidate_protein_hits	included_safe_predictor	numeric, variable, prespecified as non-leaky and non-identifier	36	0	5	pathway_biosynthetic
PWY_SALIDROSIDE__n_specific_candidate_hits	included_safe_predictor	numeric, variable, prespecified as non-leaky and non-identifier	36	0	2	pathway_biosynthetic
PWY_SALIDROSIDE__pathway_potential_score	included_safe_predictor	numeric, variable, prespecified as non-leaky and non-identifier	36	0	4	pathway_biosynthetic
PWY_TERPENOID__max_specificity_score	included_safe_predictor	numeric, variable, prespecified as non-leaky and non-identifier	36	0	4	pathway_biosynthetic
PWY_TERPENOID__n_candidate_protein_hits	included_safe_predictor	numeric, variable, prespecified as non-leaky and non-identifier	36	0	6	pathway_biosynthetic
PWY_TERPENOID__n_specific_candidate_hits	included_safe_predictor	numeric, variable, prespecified as non-leaky and non-identifier	36	0	5	pathway_biosynthetic
PWY_TERPENOID__pathway_potential_score	included_safe_predictor	numeric, variable, prespecified as non-leaky and non-identifier	36	0	5	pathway_biosynthetic
abs_latitude	included_safe_predictor	numeric, variable, prespecified as non-leaky and non-identifier	36	0	6	environment_spatial
coordinate_uncertainty_log1p	included_safe_predictor	numeric, variable, prespecified as non-leaky and non-identifier	36	0	5	coverage_control
coordinate_uncertainty_m	included_safe_predictor	numeric, variable, prespecified as non-leaky and non-identifier	36	0	5	coverage_control
cos_latitude	included_safe_predictor	numeric, variable, prespecified as non-leaky and non-identifier	36	0	6	environment_spatial
cos_longitude	included_safe_predictor	numeric, variable, prespecified as non-leaky and non-identifier	36	0	6	environment_spatial
decimal_latitude	included_safe_predictor	numeric, variable, prespecified as non-leaky and non-identifier	36	0	6	environment_spatial
decimal_longitude	included_safe_predictor	numeric, variable, prespecified as non-leaky and non-identifier	36	0	6	environment_spatial
lat_max	included_safe_predictor	numeric, variable, prespecified as non-leaky and non-identifier	36	0	6	occurrence_coverage
lat_min	included_safe_predictor	numeric, variable, prespecified as non-leaky and non-identifier	36	0	6	occurrence_coverage
lat_range	included_safe_predictor	numeric, variable, prespecified as non-leaky and non-identifier	36	0	6	environment_spatial
latitude_squared	included_safe_predictor	numeric, variable, prespecified as non-leaky and non-identifier	36	0	6	environment_spatial
lon_max	included_safe_predictor	numeric, variable, prespecified as non-leaky and non-identifier	36	0	6	occurrence_coverage
lon_min	included_safe_predictor	numeric, variable, prespecified as non-leaky and non-identifier	36	0	6	occurrence_coverage
lon_range	included_safe_predictor	numeric, variable, prespecified as non-leaky and non-identifier	36	0	6	environment_spatial
longitude_squared	included_safe_predictor	numeric, variable, prespecified as non-leaky and non-identifier	36	0	6	environment_spatial
matched_pathway_candidate_protein_hits	included_safe_predictor	numeric, variable, prespecified as non-leaky and non-identifier	36	0	16	other_retained_numeric
matched_pathway_max_specificity_score	included_safe_predictor	numeric, variable, prespecified as non-leaky and non-identifier	36	0	4	matched_pathway
matched_pathway_potential_score	included_safe_predictor	numeric, variable, prespecified as non-leaky and non-identifier	36	0	9	matched_pathway
matched_pathway_specific_candidate_hits	included_safe_predictor	numeric, variable, prespecified as non-leaky and non-identifier	36	0	6	matched_pathway
matched_pathway_specific_support_present	included_safe_predictor	numeric, variable, prespecified as non-leaky and non-identifier	36	0	2	matched_pathway
molecular_weight	included_safe_predictor	numeric, variable, prespecified as non-leaky and non-identifier	36	0	6	chemical_descriptor
n_chloroplast_plastid_records	included_safe_predictor	numeric, variable, prespecified as non-leaky and non-identifier	36	0	6	genetic_availability
n_countries	included_safe_predictor	numeric, variable, prespecified as non-leaky and non-identifier	36	0	5	occurrence_coverage
n_ena_rnaseq_runs	included_safe_predictor	numeric, variable, prespecified as non-leaky and non-identifier	36	0	6	genetic_availability
n_occurrence_rows	included_safe_predictor	numeric, variable, prespecified as non-leaky and non-identifier	36	0	6	occurrence_coverage
n_pathways_with_specific_candidates	included_safe_predictor	numeric, variable, prespecified as non-leaky and non-identifier	36	0	4	genetic_availability
n_public_barcode_records	included_safe_predictor	numeric, variable, prespecified as non-leaky and non-identifier	36	0	6	genetic_availability
n_public_genome_assemblies	included_safe_predictor	numeric, variable, prespecified as non-leaky and non-identifier	36	0	6	genetic_availability
n_spatial_blocks	included_safe_predictor	numeric, variable, prespecified as non-leaky and non-identifier	36	0	6	coverage_control
n_unique_gbif_records	included_safe_predictor	numeric, variable, prespecified as non-leaky and non-identifier	36	0	6	occurrence_coverage

Column	Status	Reason	Non-missing	Missing	Numeric unique	Feature block
pathway_support_score	included_safe_predictor	numeric, variable, prespecified as non-leaky and non-identifier	36	0	5	pathway_biosynthetic
sin_latitude	included_safe_predictor	numeric, variable, prespecified as non-leaky and non-identifier	36	0	6	environment_spatial
sin_longitude	included_safe_predictor	numeric, variable, prespecified as non-leaky and non-identifier	36	0	6	environment_spatial
sum_pathway_potential_score	included_safe_predictor	numeric, variable, prespecified as non-leaky and non-identifier	36	0	6	pathway_biosynthetic

Table S3. Completeness of final environmental predictors

Table S3A. SoilGrids predictors

Source: soilgrids_notebook3S_v2_soil_niche_outputs/derived/taxon_spatial_block_soil_primary_table.tsv (248 taxon × exact-cell rows).

Feature	Rows	Non-missing	Missing	Missing (%)	Unique values
soil_cec__0_5cm__mean	248	248	0	0.000	141
soil_cec__15_30cm__mean	248	248	0	0.000	130
soil_cec__5_15cm__mean	248	248	0	0.000	140
soil_clay__0_5cm__mean	248	248	0	0.000	158
soil_clay__15_30cm__mean	248	248	0	0.000	151
soil_clay__5_15cm__mean	248	248	0	0.000	156
soil_nitrogen__0_5cm__mean	248	248	0	0.000	177
soil_nitrogen__15_30cm__mean	248	248	0	0.000	158
soil_nitrogen__5_15cm__mean	248	248	0	0.000	160
soil_phh2o__0_5cm__mean	248	248	0	0.000	42
soil_phh2o__15_30cm__mean	248	248	0	0.000	45
soil_phh2o__5_15cm__mean	248	248	0	0.000	44
soil_sand__0_5cm__mean	248	248	0	0.000	163
soil_sand__15_30cm__mean	248	248	0	0.000	167
soil_sand__5_15cm__mean	248	248	0	0.000	163
soil_silt__0_5cm__mean	248	248	0	0.000	162
soil_silt__15_30cm__mean	248	248	0	0.000	142
soil_silt__5_15cm__mean	248	248	0	0.000	154
soil_soc__0_5cm__mean	248	248	0	0.000	177
soil_soc__15_30cm__mean	248	248	0	0.000	162
soil_soc__5_15cm__mean	248	248	0	0.000	174

Table S3B. NASA POWER predictors

Source: climate_notebook3C_niche_outputs/derived/taxon_spatial_block_climate_table.tsv (324 taxon × exact-cell rows).

Feature	Rows	Non-missing	Missing	Missing (%)	Unique values
clim_tmean_annual	324	324	0	0.000	198
clim_tmean_seasonality_sd	324	324	0	0.000	201
clim_tmean_range	324	324	0	0.000	200
clim_tmax_warmest_month	324	324	0	0.000	198
clim_tmin_coldest_month	324	324	0	0.000	198
clim_temp_extreme_range	324	324	0	0.000	198
clim_mean_diurnal_range	324	324	0	0.000	201
clim_precip_annual_total_approx_mm	324	324	0	0.000	206
clim_precip_wettest_month_total_approx_mm	324	324	0	0.000	184
clim_precip_driest_month_total_approx_mm	324	324	0	0.000	168
clim_precip_total_seasonality_cv	324	324	0	0.000	203
clim_aridity_index_annual_precip_total_over_tmean_plus10	324	324	0	0.000	202
clim_precip_wettest_quarter_total_approx_mm	324	324	0	0.000	201
clim_precip_driest_quarter_total_approx_mm	324	324	0	0.000	197
clim_tmean_warmest_quarter	324	324	0	0.000	193
clim_tmean_coldest_quarter	324	324	0	0.000	200
clim_tmean_wettest_quarter	324	324	0	0.000	198
clim_tmean_driest_quarter	324	324	0	0.000	197
clim_precip_warmest_quarter_total_approx_mm	324	324	0	0.000	203
clim_precip_coldest_quarter_total_approx_mm	324	324	0	0.000	202
clim_rh2m_annual	324	324	0	0.000	201
clim_rh2m_seasonality_sd	324	324	0	0.000	203
clim_solar_annual_mean	324	324	0	0.000	181
clim_solar_seasonality_sd	324	324	0	0.000	189
clim_wind_annual_mean	324	324	0	0.000	198
clim_wind_seasonality_sd	324	324	0	0.000	201
clim_T2M_JAN	324	324	0	0.000	192
clim_PRECTOTCORR_RATE_JAN	324	324	0	0.000	169
clim_RH2M_JAN	324	324	0	0.000	196
clim_T2M_FEB	324	324	0	0.000	188
clim_PRECTOTCORR_RATE_FEB	324	324	0	0.000	164
clim_RH2M_FEB	324	324	0	0.000	203
clim_T2M_MAR	324	324	0	0.000	199
clim_PRECTOTCORR_RATE_MAR	324	324	0	0.000	165
clim_RH2M_MAR	324	324	0	0.000	198
clim_T2M_APR	324	324	0	0.000	191
clim_PRECTOTCORR_RATE_APR	324	324	0	0.000	159
clim_RH2M_APR	324	324	0	0.000	191
clim_T2M_MAY	324	324	0	0.000	196
clim_PRECTOTCORR_RATE_MAY	324	324	0	0.000	166
clim_RH2M_MAY	324	324	0	0.000	197
clim_T2M_JUN	324	324	0	0.000	194
clim_PRECTOTCORR_RATE_JUN	324	324	0	0.000	175
clim_RH2M_JUN	324	324	0	0.000	194
clim_T2M_JUL	324	324	0	0.000	194
clim_PRECTOTCORR_RATE_JUL	324	324	0	0.000	174
clim_RH2M_JUL	324	324	0	0.000	198
clim_T2M_AUG	324	324	0	0.000	189
clim_PRECTOTCORR_RATE_AUG	324	324	0	0.000	159
clim_RH2M_AUG	324	324	0	0.000	196
clim_T2M_SEP	324	324	0	0.000	193
clim_PRECTOTCORR_RATE_SEP	324	324	0	0.000	176
clim_RH2M_SEP	324	324	0	0.000	192
clim_T2M_OCT	324	324	0	0.000	198
clim_PRECTOTCORR_RATE_OCT	324	324	0	0.000	165
clim_RH2M_OCT	324	324	0	0.000	194
clim_T2M_NOV	324	324	0	0.000	199
clim_PRECTOTCORR_RATE_NOV	324	324	0	0.000	173
clim_RH2M_NOV	324	324	0	0.000	203

Feature	Rows	Non-missing	Missing	Missing (%)	Unique values
clim_T2M_DEC	324	324	0	0.000	194
clim_PRECTOTCORR_RATE_DEC	324	324	0	0.000	171
clim_RH2M_DEC	324	324	0	0.000	201

Table S4. Grouped genetic-model metrics and fold eligibility

Table S4A. Selected complete grouped-validation metrics

Source: table2_grouped_classification_results_complete_v3.tsv. BA intervals are empirical 2.5th–97.5th percentiles across valid held-out-group folds. Confusion entries are TN/FP/FN/TP.

Analysis	Scheme	Feature block	Model	Folds	Accuracy	BA [fold interval]	Precision	Recall	F1	MCC	ROC-AUC	AP	TN/FP/FN/TP
Primary RF	LOTO	compound_matched_core	random_forest_balanced	6	0.694	0.621 [0.500–0.800]	0.250	0.500	0.317	0.191	0.425	0.361	21/7/4/4
Post hoc logistic	LOTO	compound_matched_core	logistic_l2_balanced	6	0.667	0.646 [0.500–0.794]	0.247	0.667	0.356	0.263	0.733	0.624	18/10/2/6
Gradient comparator	LOTO	compound_matched_core	gradient_boosting	6	0.667	0.475 [0.309–0.828]	0.111	0.167	0.133	-0.054	0.492	0.339	22/6/6/2
Matched-pathway only	LOTO	matched_pathway	random_forest_balanced	6	0.694	0.662 [0.500–0.800]	0.261	0.667	0.373	0.285	0.662	0.444	19/9/2/6
All retained numeric	LOTO	combined_safe	random_forest_balanced	6	0.694	0.579 [0.391–0.800]	0.111	0.333	0.167	0.096	0.575	0.375	23/5/6/2
Molecular weight only	LOTO	chemical_descriptor	random_forest_balanced	6	0.556	0.358 [0.300–0.400]	0.000	0.000	0.000	-0.277	0.142	0.239	20/8/8/0
Coverage control	LOTO	coverage_control	random_forest_balanced	6	0.778	0.500 [0.500–0.500]	0.000	0.000	0.000	0.000	0.500	0.222	28/0/8/0
Cross-molecule RF	LOCO	compound_matched_core	random_forest_balanced	6	0.778	0.746 [0.516–0.975]	0.528	0.667	0.528	0.463	0.794	0.686	23/5/3/5

Table S4B. Mixed-record LOCCO fold-eligibility audit

Source: mixed_record_locco_fold_eligibility_audit.tsv. The audit applies to the complete 60-row inventory and is not the primary performance analysis.

held_out_group	n_rows	n_supported	n_background	n_label_classes	fold_status
alkaloids	6	4	2	2	valid
diterpenoid taxane	6	2	4	2	valid
flavonoids	6	6	0	1	excluded_single_class_test_fold
naphthodianthrone	6	1	5	2	valid
phenolic alcohol	6	1	5	2	valid
phenolic compounds	6	6	0	1	excluded_single_class_test_fold
phenolic glycoside	6	2	4	2	valid
sesquiterpene lactone	6	1	5	2	valid
terpenoids	6	3	3	2	valid
triterpenoid saponin	6	1	5	2	valid

Table S5. Positive–unlabelled and label-permutation sensitivity

Table S5A. Positive–unlabelled background sensitivity

Source: positive_unlabelled_background_sensitivity_summary_v4.tsv. Reduced-background results are sensitivity diagnostics; some held-out taxa contained one class and were invalid for BA.

Strategy	Background ratio	Repeats	Supported	Background median	Valid folds median/min	Mean BA	BA 2.5–97.5%	Mean F1	Mean ROC-AUC	Mean AP
compound_class_stratified	0.25	30	8	2.0	2.0 / 2	0.750	0.500–1.000	0.800	1.000	1.000
compound_class_stratified	0.50	30	8	4.0	4.0 / 4	0.854	0.750–1.000	0.769	1.000	1.000
compound_class_stratified	1.00	30	8	8.0	5.0 / 3	0.709	0.540–0.842	0.540	0.624	0.726
full_unlabelled_background	full	1	8	28.0	6.0 / 6	0.621	0.621–0.621	0.317	0.425	0.361
global_random	0.25	25	8	2.0	2.0 / 2	0.530	0.250–0.750	0.676	0.620	0.823
global_random	0.50	30	8	4.0	3.0 / 2	0.628	0.448–0.833	0.580	0.718	0.840
global_random	1.00	30	8	8.0	5.0 / 3	0.641	0.447–0.779	0.493	0.604	0.718
taxon_stratified	1.00	30	8	8.0	6.0 / 6	0.609	0.455–0.750	0.410	0.594	0.765
two_way_stratified	1.00	30	8	8.0	2.0 / 2	0.650	0.650–0.650	0.250	0.633	0.458

Table S5B. Primary label-permutation control

Source: label_permutation_null_control_summary_v2.tsv; one-sided plus-one empirical P value.

selected_scheme	selected_feature_block	selected_model	observed_balanced_accuracy	null_mean_balanced_accuracy	null_sd_balanced_accuracy	empirical_p_value	n_permutations
leave_one_taxon_out	compound_matched_core	random_forest_balanced	0.620833	0.493874	0.115046	0.154229	200

Table S6. Environmental niche sensitivity and restricted multivariate tests

Table S6A-1. Prespecified environmental taxon-classifier metrics

Sources: taxon_soil_classification_complete_summary.tsv and taxon_climate_classification_complete_summary.tsv. Intervals are Student-t intervals across five grouped folds; permutation P is shown for primary exact-cell grouping.

Layer	Validation	Model	Folds	Accuracy	BA [t interval]	Macro precision	Macro recall	Macro F1	MCC	Macro ROC-AUC	Perm. P
Soil	coarse_1_degree_spatial_cluster_group_kfold_sensitivity	random_forest	5	0.528	0.492 [0.427–0.557]	0.465	0.492	0.443	0.429	0.790	—
Soil	exact_0.25_degree_cell_group_kfold	random_forest	5	0.512	0.479 [0.422–0.537]	0.443	0.479	0.437	0.417	0.812	0.005
Climate	coarse_1_degree_spatial_cluster_group_kfold_sensitivity	logistic_multinomial	5	0.503	0.551 [0.498–0.604]	0.521	0.551	0.509	0.412	0.823	—
Climate	exact_0.25_degree_cell_group_kfold	logistic_multinomial	5	0.515	0.547 [0.483–0.612]	0.539	0.547	0.510	0.433	0.815	0.005

Table S6A-2. Restricted global tests

Taxon-label permutations were restricted within 1° spatial clusters.

Layer	PERMANOVA pseudo-F	PERMANOVA R^2	PERMANOVA P	Permutations	Dispersion F	Dispersion P	Permutations
Soil	16.721	0.257	0.067	999	10.567	0.266	999
Climate	19.371	0.233	0.075	999	6.736	0.574	999

Table S6A-3. Balanced block-subsampling sensitivity

The grouped classifier in this sensitivity analysis is a common auxiliary multinomial logistic model for both layers; it is not the prespecified primary soil random forest.

Layer	Repeats	Rows/taxon	Total rows	Median R^2 [2.5–97.5%]	PERMANOVA P<0.05 fraction	Median dispersion F	Dispersion P<0.05 fraction	Auxiliary logistic median BA [2.5–97.5%]
Soil	100	33	198	0.261 [0.235–0.287]	0.080	8.297	0.040	0.434 [0.379–0.487]
Climate	100	42	252	0.267 [0.257–0.281]	0.060	5.386	0.000	0.539 [0.497–0.576]

Table S6B. Restricted pairwise PERMANOVA with BH adjustment

All pairwise tests used label permutations restricted within 1° clusters; P values were adjusted within each environmental layer by Benjamini–Hochberg.

Layer	Group A	Group B	Pseudo-F	R^2	Raw P	BH P	n	1° strata	Permutations
Soil	Artemisia annua	Glycyrrhiza glabra	18.239	0.194	1.000	1.000	78	58	499
Soil	Artemisia annua	Hypericum	9.202	0.090	0.406	0.960	95	74	499
Soil	Artemisia annua	Rhodiola	18.896	0.213	0.358	0.960	72	64	499
Soil	Artemisia annua	Sedum	5.861	0.080	0.374	0.960	69	54	499
Soil	Artemisia annua	Taxus	7.897	0.110	0.512	0.960	66	50	499
Soil	Glycyrrhiza glabra	Hypericum	38.639	0.269	0.240	0.960	107	77	499
Soil	Glycyrrhiza glabra	Rhodiola	58.843	0.418	1.000	1.000	84	68	499
Soil	Glycyrrhiza glabra	Sedum	33.556	0.298	0.680	1.000	81	59	499
Soil	Glycyrrhiza glabra	Taxus	46.742	0.381	1.000	1.000	78	56	499
Soil	Hypericum	Rhodiola	14.447	0.127	1.000	1.000	101	84	499
Soil	Hypericum	Sedum	0.706	0.007	0.750	1.000	98	62	499
Soil	Hypericum	Taxus	3.519	0.036	0.596	0.993	95	63	499
Soil	Rhodiola	Sedum	14.414	0.165	0.314	0.960	75	65	499
Soil	Rhodiola	Taxus	5.238	0.070	0.342	0.960	72	59	499
Soil	Sedum	Taxus	3.774	0.053	0.492	0.960	69	45	499
Climate	Artemisia annua	Glycyrrhiza glabra	19.483	0.176	1.000	1.000	93	68	499
Climate	Artemisia annua	Hypericum	5.784	0.046	0.146	0.660	121	90	499
Climate	Artemisia annua	Rhodiola	23.021	0.204	0.506	0.698	92	82	499
Climate	Artemisia annua	Sedum	3.982	0.034	0.314	0.698	116	80	499
Climate	Artemisia annua	Taxus	10.809	0.098	0.176	0.660	102	70	499
Climate	Glycyrrhiza glabra	Hypericum	28.443	0.203	0.170	0.660	114	83	499
Climate	Glycyrrhiza glabra	Rhodiola	61.714	0.426	1.000	1.000	85	73	499
Climate	Glycyrrhiza glabra	Sedum	29.526	0.216	0.758	0.948	109	75	499
Climate	Glycyrrhiza glabra	Taxus	55.182	0.372	1.000	1.000	95	65	499
Climate	Hypericum	Rhodiola	26.810	0.195	0.468	0.698	113	94	499
Climate	Hypericum	Sedum	2.378	0.017	0.168	0.660	137	82	499
Climate	Hypericum	Taxus	8.338	0.064	0.452	0.698	123	75	499
Climate	Rhodiola	Sedum	24.127	0.185	0.512	0.698	108	88	499
Climate	Rhodiola	Taxus	14.296	0.134	0.222	0.666	94	73	499
Climate	Sedum	Taxus	4.517	0.037	0.484	0.698	118	64	499

Table S7. Prespecified environmental compound negative controls

Each control contains 48 rows (six taxa × eight shared annotations; 10 supported, 38 unlabelled background). Intervals are Student-t intervals across valid grouped folds.

Layer	Validation	Model	Folds	Accuracy	BA [t interval]	Precision	Recall	F1	MCC	ROC-AUC	AP	Perm. P
Soil	leave_one_compound_class_out	random_forest	8	0.500	0.406 [0.206–0.606]	0.167	0.250	0.188	-0.128	0.225	0.333	0.736
Soil	leave_one_taxon_out	random_forest	6	0.667	0.500 [0.500–0.500]	0.021	0.167	0.037	0.000	0.500	0.208	1.000
Climate	leave_one_compound_class_out	logistic_l2_balanced	8	0.500	0.406 [0.206–0.606]	0.167	0.250	0.188	-0.128	0.312	0.375	0.692
Climate	leave_one_taxon_out	logistic_l2_balanced	6	0.417	0.500 [0.500–0.500]	0.146	0.667	0.232	0.000	0.500	0.208	1.000

Table S8. Molecule-only LOTO out-of-fold ranking audit

Source: table4_taxon_compound_priorities_oof_loto_v3.tsv. Probabilities are taxon-held-out exploratory evidence-prioritization scores, not probabilities of metabolite presence.

Taxon	Molecule	Compound class	Evidence score	Open-data label	LOTO OOF probability	Predicted class	Validation	Model	Feature block
Artemisia annua	paclitaxel	diterpenoid taxane	1	0	0.771362	1	leave_one_taxon_out	random_forest_balanced	compound_matched_core
Artemisia annua	glycyrrhizin	triterpenoid saponin	1	0	0.727879	1	leave_one_taxon_out	random_forest_balanced	compound_matched_core
Glycyrrhiza glabra	artemisinin	sesquiterpene lactone	0	0	0.709111	1	leave_one_taxon_out	random_forest_balanced	compound_matched_core
Taxus	artemisinin	sesquiterpene lactone	1	0	0.694734	1	leave_one_taxon_out	random_forest_balanced	compound_matched_core
Taxus	glycyrrhizin	triterpenoid saponin	0	0	0.611661	1	leave_one_taxon_out	random_forest_balanced	compound_matched_core
Artemisia annua	artemisinin	sesquiterpene lactone	3	1	0.593953	1	leave_one_taxon_out	random_forest_balanced	compound_matched_core
Taxus	paclitaxel	diterpenoid taxane	3	1	0.579603	1	leave_one_taxon_out	random_forest_balanced	compound_matched_core
Rhodiola	salidroside	phenolic glycoside	3	1	0.554279	1	leave_one_taxon_out	random_forest_balanced	compound_matched_core
Rhodiola	paclitaxel	diterpenoid taxane	1	0	0.546229	1	leave_one_taxon_out	random_forest_balanced	compound_matched_core
Glycyrrhiza glabra	hypericin	naphthodianthrone	1	0	0.536337	1	leave_one_taxon_out	random_forest_balanced	compound_matched_core
Glycyrrhiza glabra	paclitaxel	diterpenoid taxane	2	1	0.506572	1	leave_one_taxon_out	random_forest_balanced	compound_matched_core
Artemisia annua	hypericin	naphthodianthrone	1	0	0.496516	0	leave_one_taxon_out	random_forest_balanced	compound_matched_core
Rhodiola	glycyrrhizin	triterpenoid saponin	0	0	0.487918	0	leave_one_taxon_out	random_forest_balanced	compound_matched_core
Rhodiola	tyrosol	phenolic alcohol	3	1	0.455751	0	leave_one_taxon_out	random_forest_balanced	compound_matched_core
Glycyrrhiza glabra	glycyrrhizin	triterpenoid saponin	3	1	0.444250	0	leave_one_taxon_out	random_forest_balanced	compound_matched_core
Rhodiola	artemisinin	sesquiterpene lactone	0	0	0.436822	0	leave_one_taxon_out	random_forest_balanced	compound_matched_core
Hypericum	salidroside	phenolic glycoside	1	0	0.433213	0	leave_one_taxon_out	random_forest_balanced	compound_matched_core
Hypericum	tyrosol	phenolic alcohol	1	0	0.307337	0	leave_one_taxon_out	random_forest_balanced	compound_matched_core
Taxus	salidroside	phenolic glycoside	0	0	0.185447	0	leave_one_taxon_out	random_forest_balanced	compound_matched_core
Glycyrrhiza glabra	salidroside	phenolic glycoside	0	0	0.127922	0	leave_one_taxon_out	random_forest_balanced	compound_matched_core
Artemisia annua	salidroside	phenolic glycoside	0	0	0.126253	0	leave_one_taxon_out	random_forest_balanced	compound_matched_core
Taxus	hypericin	naphthodianthrone	0	0	0.064340	0	leave_one_taxon_out	random_forest_balanced	compound_matched_core
Hypericum	artemisinin	sesquiterpene lactone	1	0	0.060549	0	leave_one_taxon_out	random_forest_balanced	compound_matched_core
Taxus	tyrosol	phenolic alcohol	0	0	0.052211	0	leave_one_taxon_out	random_forest_balanced	compound_matched_core
Rhodiola	hypericin	naphthodianthrone	0	0	0.041548	0	leave_one_taxon_out	random_forest_balanced	compound_matched_core
Hypericum	paclitaxel	diterpenoid taxane	1	0	0.039032	0	leave_one_taxon_out	random_forest_balanced	compound_matched_core
Glycyrrhiza glabra	tyrosol	phenolic alcohol	0	0	0.038281	0	leave_one_taxon_out	random_forest_balanced	compound_matched_core
Sedum	hypericin	naphthodianthrone	0	0	0.029265	0	leave_one_taxon_out	random_forest_balanced	compound_matched_core
Hypericum	glycyrrhizin	triterpenoid saponin	0	0	0.026947	0	leave_one_taxon_out	random_forest_balanced	compound_matched_core
Artemisia annua	tyrosol	phenolic alcohol	0	0	0.026461	0	leave_one_taxon_out	random_forest_balanced	compound_matched_core
Hypericum	hypericin	naphthodianthrone	3	1	0.023421	0	leave_one_taxon_out	random_forest_balanced	compound_matched_core
Sedum	paclitaxel	diterpenoid taxane	0	0	0.020765	0	leave_one_taxon_out	random_forest_balanced	compound_matched_core

Taxon	Molecule	Compound class	Evidence score	Open-data label	LOTO OOF probability	Predicted class	Validation	Model	Feature block
Sedum	glycyrrhizin	triterpenoid saponin	0	0	0.015363	0	leave_one_taxon_out	random_forest_balanced	compound_matched_core
Sedum	tyrosol	phenolic alcohol	0	0	0.001669	0	leave_one_taxon_out	random_forest_balanced	compound_matched_core
Sedum	artemisinin	sesquiterpene lactone	0	0	0.000876	0	leave_one_taxon_out	random_forest_balanced	compound_matched_core
Sedum	salidroside	phenolic glycoside	2	1	0.000876	0	leave_one_taxon_out	random_forest_balanced	compound_matched_core
