# Supplementary material for: Integrating Biosynthetic, Genomic and Ecological Open Data for Medicinal Plant Research: A Leakage-Aware Evidence-Prioritization Framework
Source: Int J Mol Sci. 2026 Jul 29;27(15):6801. doi: 10.3390/ijms27156801 (PMC13467606; doi:10.3390/ijms27156801)
Supplement: Supplementary file 1 [file ijms-27-06801-s001.zip › med plant data revised notebooks/climate_notebook3_outputs.zip!/figures/figure4C_compound_evidence_annotation.pdf]

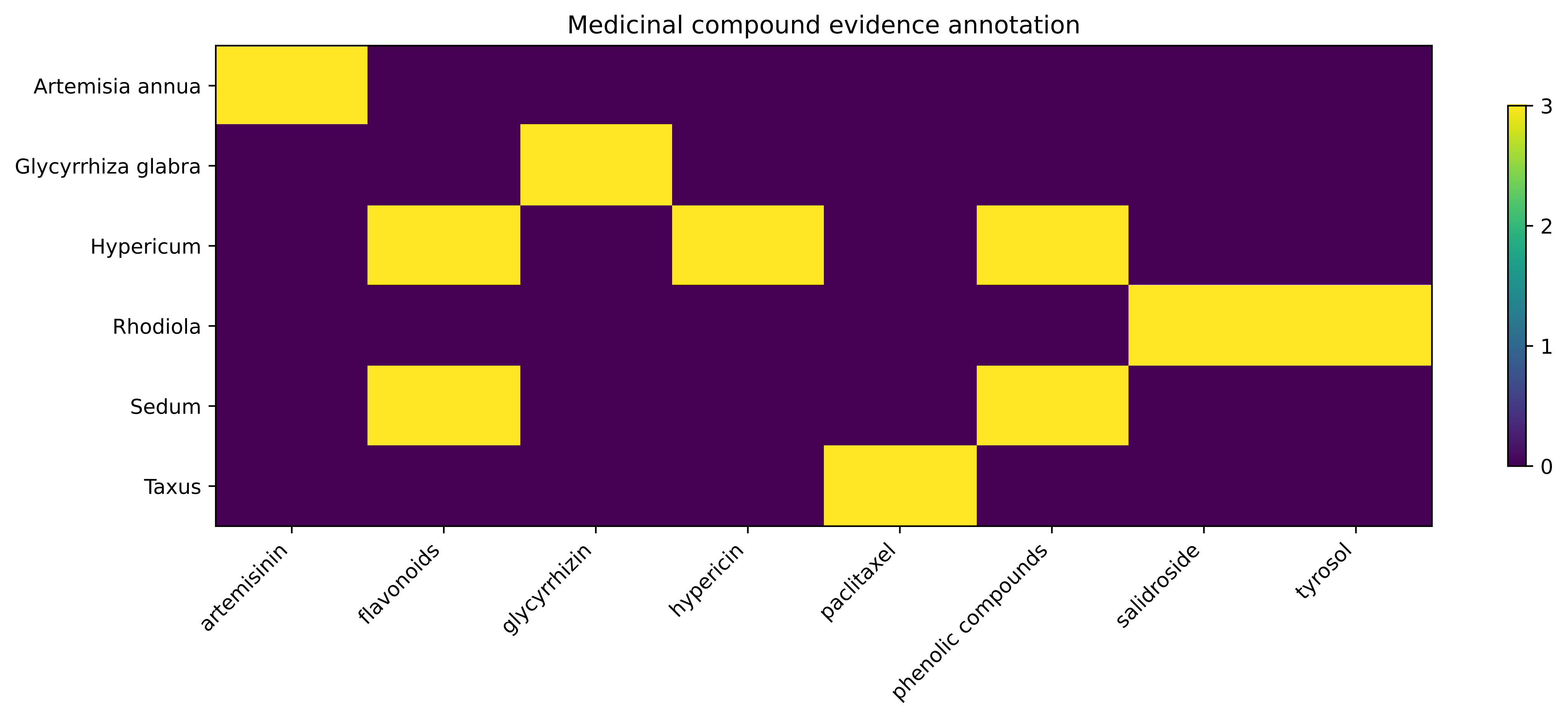

Medicinal compound evidence annotation
Artemisia annua
Glycyrrhiza glabra
Hypericum
Rhodiola
Sedum
Taxus
artemisinin
flavonoids
glycyrrhizin
hypericin
paclitaxel
phenolic compounds
salidroside
tyrosol
0
1
2
3
