# Supplementary material for: Integrating Biosynthetic, Genomic and Ecological Open Data for Medicinal Plant Research: A Leakage-Aware Evidence-Prioritization Framework
Source: Int J Mol Sci. 2026 Jul 29;27(15):6801. doi: 10.3390/ijms27156801 (PMC13467606; doi:10.3390/ijms27156801)
Supplement: Supplementary file 1 [file ijms-27-06801-s001.zip › med plant data revised notebooks/climate_notebook3_outputs.zip!/figures/figureS_climate_balanced_block_sensitivity.pdf]

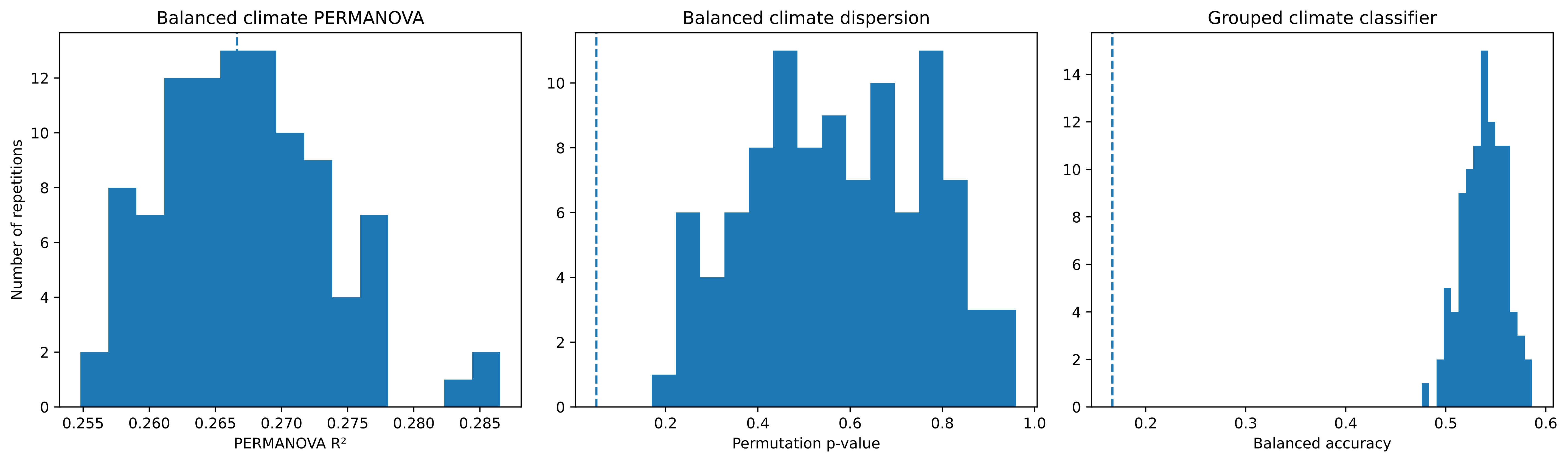

Balanced climate PERMANOVA
Number of repetitions
PERMANOVA R²
0.255
0.260
0.265
0.270
0.275
0.280
0.285
Balanced climate dispersion
Permutation p-value
0.2
0.4
0.6
0.8
1.0
Grouped climate classifier
Balanced accuracy
0.2
0.3
0.4
0.5
0.6
