# Supplementary material for: Integrating Biosynthetic, Genomic and Ecological Open Data for Medicinal Plant Research: A Leakage-Aware Evidence-Prioritization Framework
Source: Int J Mol Sci. 2026 Jul 29;27(15):6801. doi: 10.3390/ijms27156801 (PMC13467606; doi:10.3390/ijms27156801)
Supplement: Supplementary file 1 [file ijms-27-06801-s001.zip › med plant data revised notebooks/climate_notebook3_outputs.zip!/figures/figure6B_climate_compound_negative_control_permutation_tests.pdf]

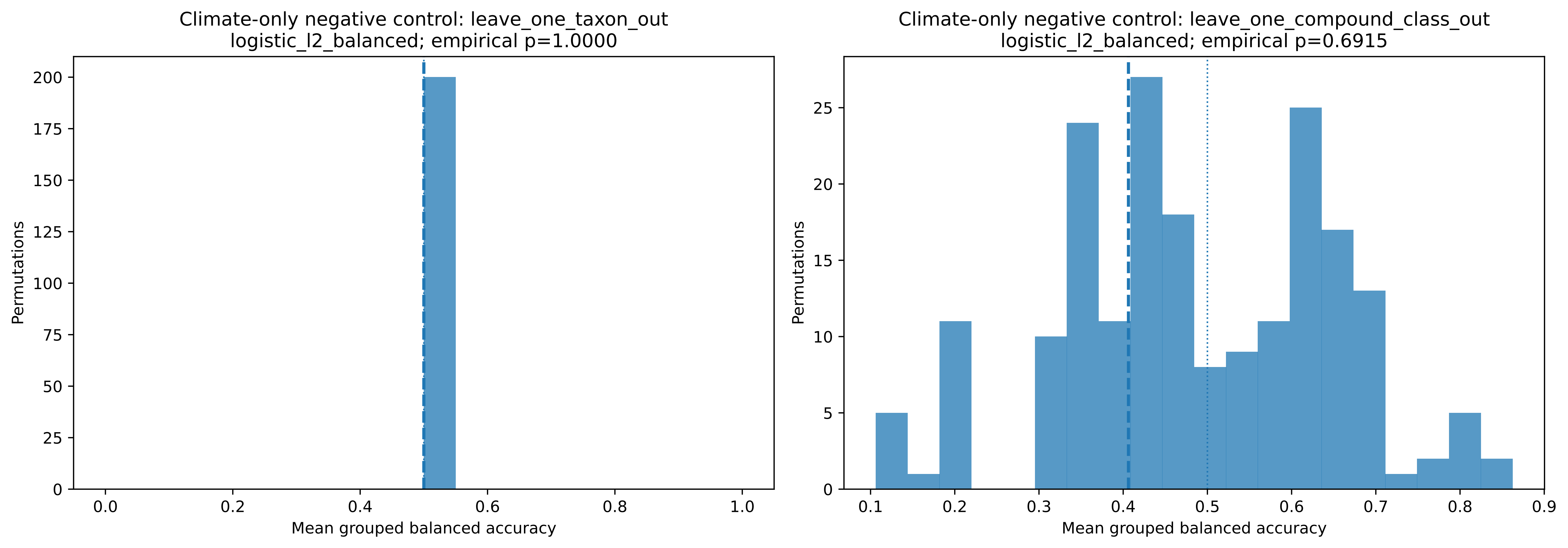
Climate-only negative control: leave_one_taxon_out
logistic_l2_balanced; empirical p=1.0000
Permutations
Mean grouped balanced accuracy
0.0
0.2
0.4
0.6
0.8
1.0
0
25
50
75
100
125
150
175
200
Climate-only negative control: leave_one_compound_class_out
logistic_l2_balanced; empirical p=0.6915
Permutations
Mean grouped balanced accuracy
0.1
0.2
0.3
0.4
0.5
0.6
0.7
0.8
0.9
0
5
10
15
20
25
