# Supplementary material for: Integrating Biosynthetic, Genomic and Ecological Open Data for Medicinal Plant Research: A Leakage-Aware Evidence-Prioritization Framework
Source: Int J Mol Sci. 2026 Jul 29;27(15):6801. doi: 10.3390/ijms27156801 (PMC13467606; doi:10.3390/ijms27156801)
Supplement: Supplementary file 1 [file ijms-27-06801-s001.zip › med plant data revised notebooks/climate_notebook3_outputs.zip!/figures/figure3C_climate_niche_differentiation_negative_control.pdf]

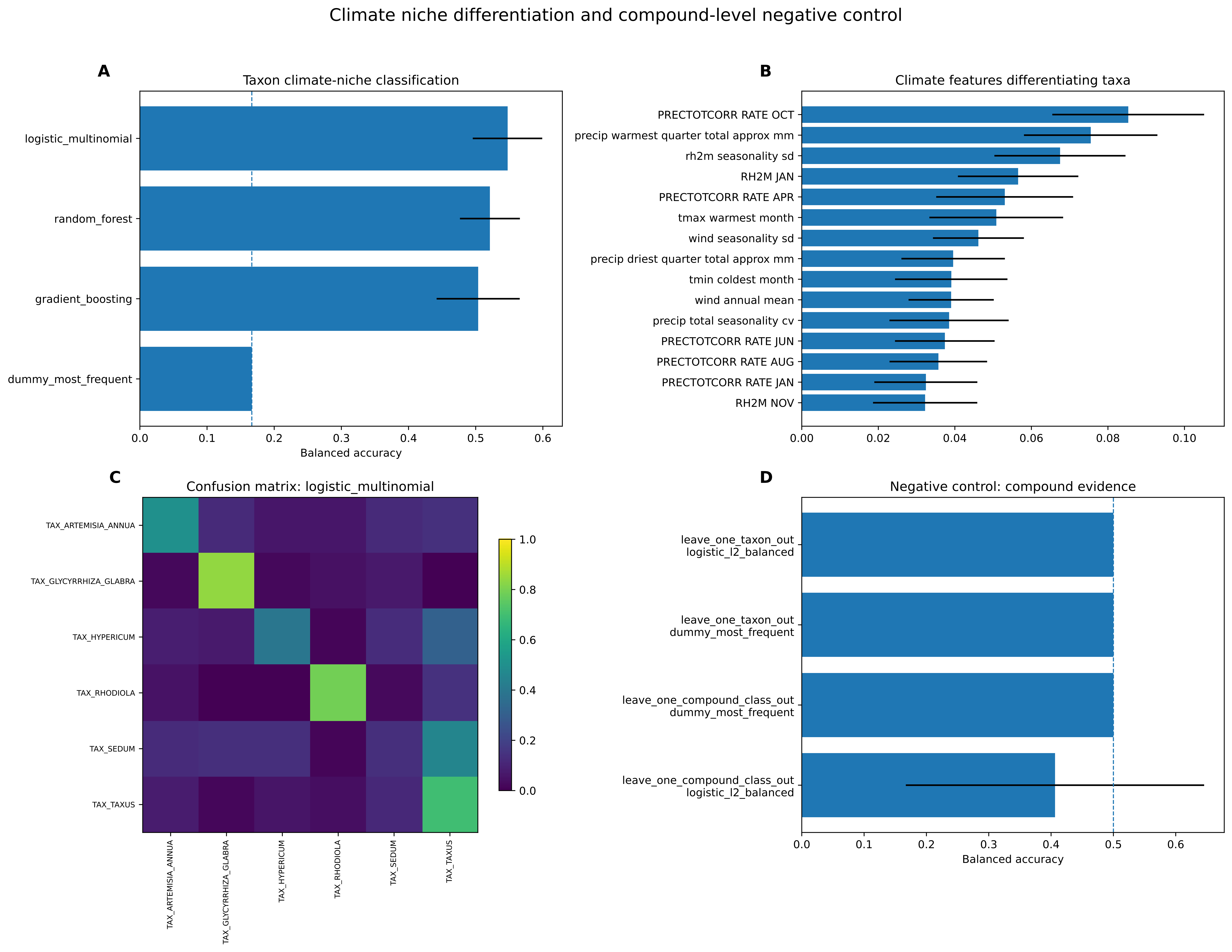

Climate niche differentiation and compound-level negative control
A
Taxon climate-niche classification
logistic_multinomial
random_forest
gradient_boosting
dummy_most_frequent
0.0
0.1
0.2
0.3
0.4
0.5
0.6
Balanced accuracy
B
Climate features differentiating taxa
PRECTOTCORR RATE OCT
precip warmest quarter total approx mm
rh2m seasonality sd
RH2M JAN
PRECTOTCORR RATE APR
tmax warmest month
wind seasonality sd
precip driest quarter total approx mm
tmin coldest month
wind annual mean
precip total seasonality cv
PRECTOTCORR RATE JUN
PRECTOTCORR RATE AUG
PRECTOTCORR RATE JAN
RH2M NOV
0.00
0.02
0.04
0.06
0.08
0.10
C
Confusion matrix: logistic_multinomial
TAX_ARTEMISIA_ANNUA
TAX_GLYCYRRHIZA_GLABRA
TAX_HYPERICUM
TAX_RHODIOLA
TAX_SEDUM
TAX_TAXUS
1.0
0.8
0.6
0.4
0.2
0.0
D
Negative control: compound evidence
leave_one_taxon_out
logistic_l2_balanced
leave_one_taxon_out
dummy_most_frequent
leave_one_compound_class_out
dummy_most_frequent
leave_one_compound_class_out
logistic_l2_balanced
0.0
0.1
0.2
0.3
0.4
0.5
0.6
Balanced accuracy
