# Supplementary material for: Integrating Biosynthetic, Genomic and Ecological Open Data for Medicinal Plant Research: A Leakage-Aware Evidence-Prioritization Framework
Source: Int J Mol Sci. 2026 Jul 29;27(15):6801. doi: 10.3390/ijms27156801 (PMC13467606; doi:10.3390/ijms27156801)
Supplement: Supplementary file 1 [file ijms-27-06801-s001.zip › med plant data revised notebooks/climate_notebook3_outputs.zip!/figures/figure5A_climate_pca_confidence_ellipses_permanova.pdf]

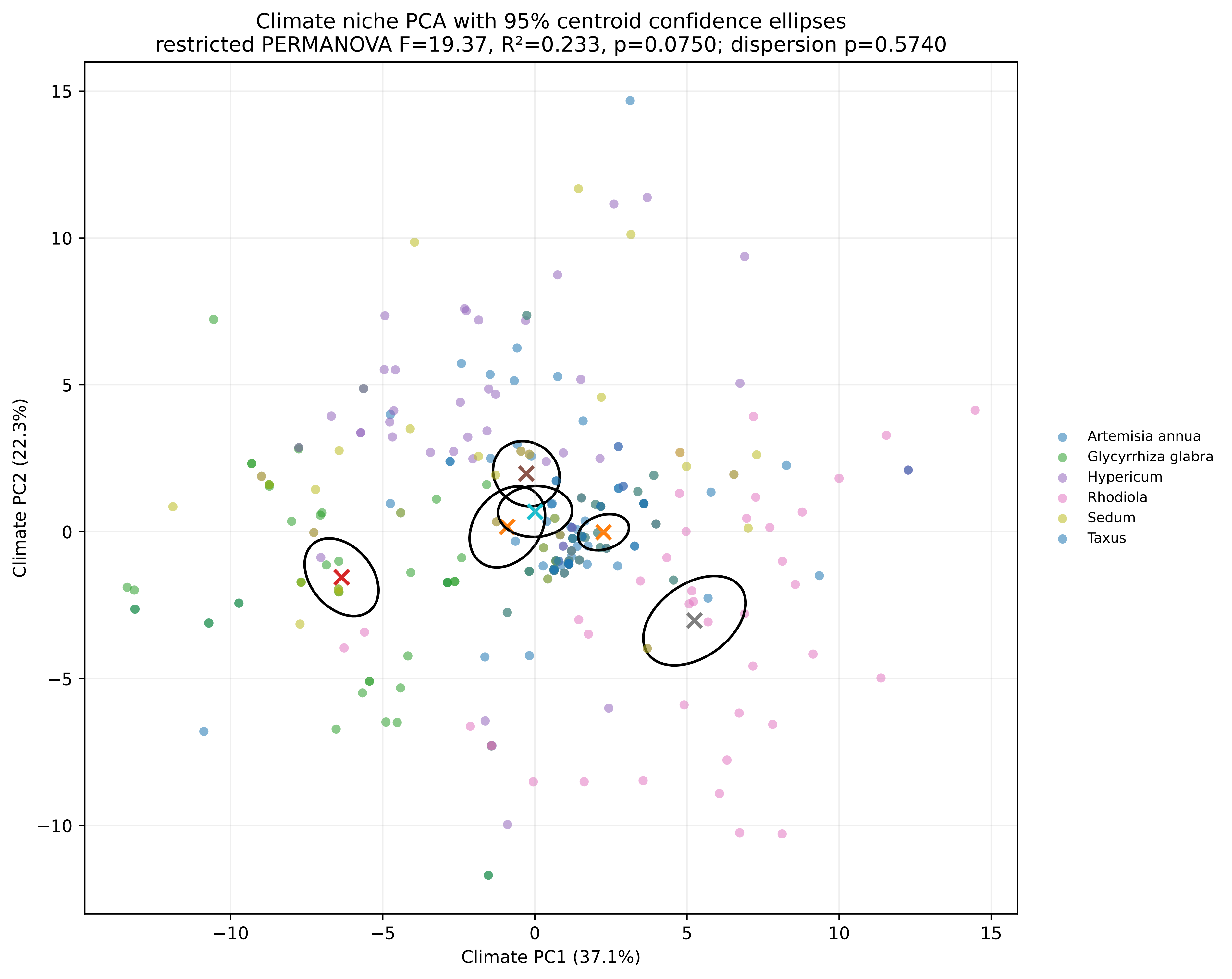
Climate niche PCA with 95% centroid confidence ellipses
restricted PERMANOVA F=19.37, R²=0.233, p=0.0750; dispersion p=0.5740
Climate PC1 (37.1%)
Climate PC2 (22.3%)
−10
−5
0
5
10
15
Artemisia annua
Glycyrrhiza glabra
Hypericum
Rhodiola
Sedum
Taxus
