# Supplementary material for: Integrating Biosynthetic, Genomic and Ecological Open Data for Medicinal Plant Research: A Leakage-Aware Evidence-Prioritization Framework
Source: Int J Mol Sci. 2026 Jul 29;27(15):6801. doi: 10.3390/ijms27156801 (PMC13467606; doi:10.3390/ijms27156801)
Supplement: Supplementary file 1 [file ijms-27-06801-s001.zip › med plant data revised notebooks/climate_notebook3_outputs.zip!/figures/figure1C_climate_niche_design_qc.pdf]

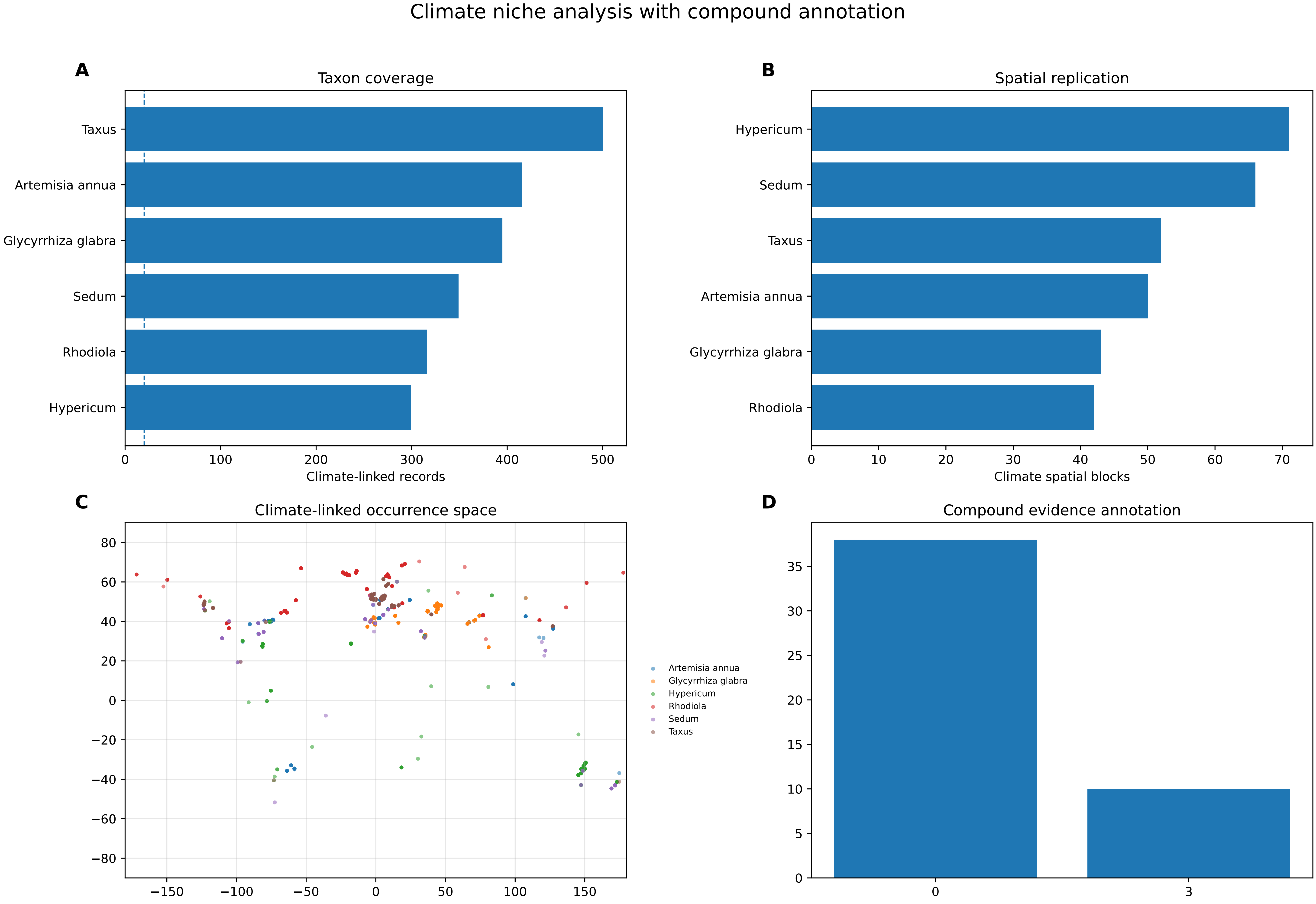

Climate niche analysis with compound annotation
A
Taxon coverage
Taxus
Artemisia annua
Glycyrrhiza glabra
Sedum
Rhodiola
Hypericum
0
100
200
300
400
500
Climate-linked records
B
Spatial replication
Hypericum
Sedum
Taxus
Artemisia annua
Glycyrrhiza glabra
Rhodiola
0
10
20
30
40
50
60
70
Climate spatial blocks
C
Climate-linked occurrence space
80
60
40
20
0
−20
−40
−60
−80
−150
−100
−50
0
50
100
150
Artemisia annua
Glycyrrhiza glabra
Hypericum
Rhodiola
Sedum
Taxus
D
Compound evidence annotation
35
30
25
20
15
10
5
0
0
3
