# Supplementary material for: Integrating Biosynthetic, Genomic and Ecological Open Data for Medicinal Plant Research: A Leakage-Aware Evidence-Prioritization Framework
Source: Int J Mol Sci. 2026 Jul 29;27(15):6801. doi: 10.3390/ijms27156801 (PMC13467606; doi:10.3390/ijms27156801)
Supplement: Supplementary file 1 [file ijms-27-06801-s001.zip › med plant data revised notebooks/genetic_notebook2_outputs.zip!/figures/figure8_pca_biplot_integrated_open_data_features.pdf]

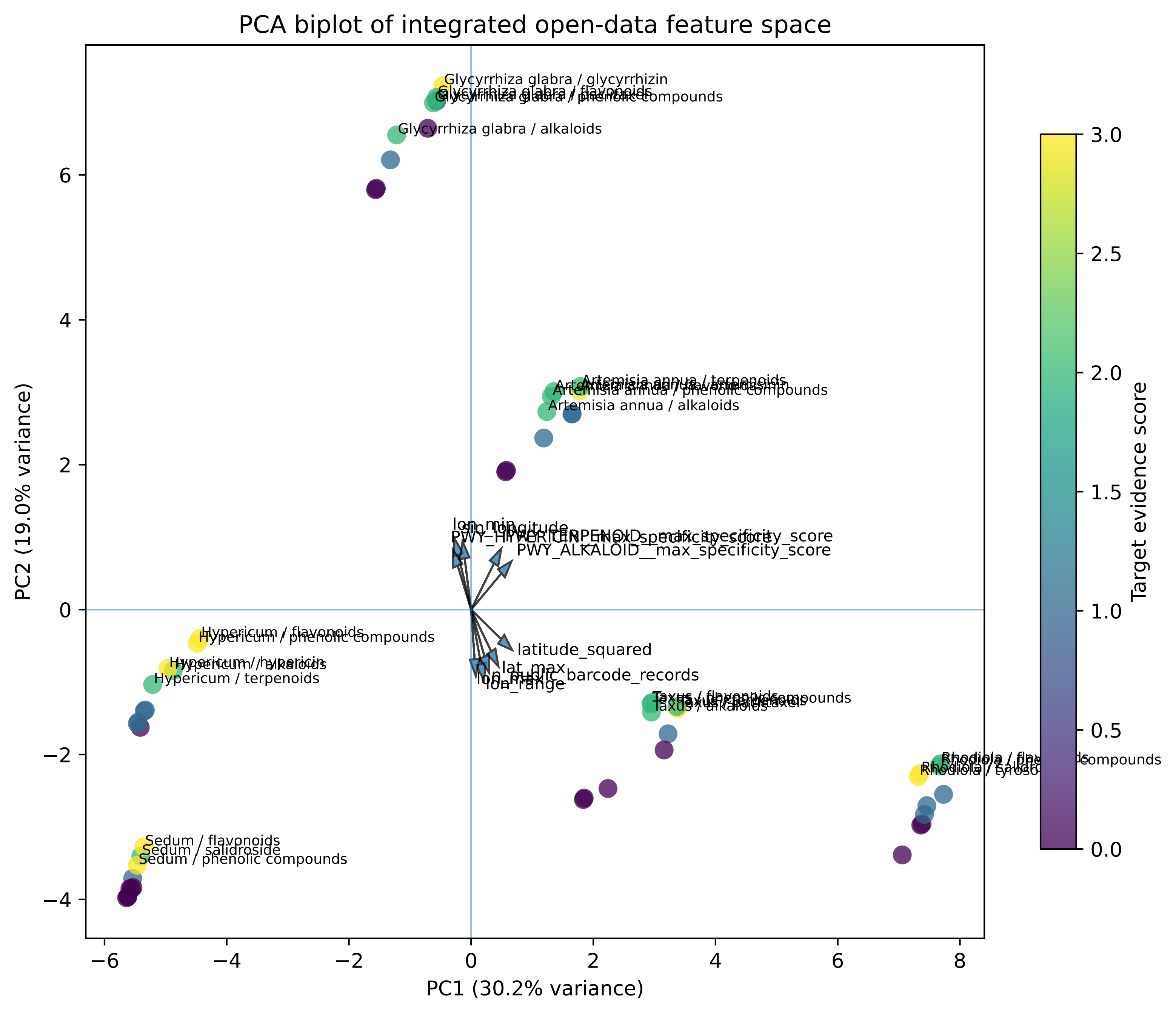

PCA biplot of integrated open-data feature space
Glycyrrhiza glabra / glycyrrhizin
Glycyrrhiza glabra / flavonoids
Glycyrrhiza glabra / phenolic compounds
Glycyrrhiza glabra / alkaloids
Artemisia annua / terpenoids
Artemisia annua / phenolic compounds
Artemisia annua / alkaloids
lon_min
sin_longitude
PWY_TERPENOID__max_specificity_score
PWY_ALKALOID__max_specificity_score
latitude_squared
lat_max
n_public_barcode_records
lon_range
Hypericum / flavonoids
Hypericum / phenolic compounds
Hypericum / hypericin
Hypericum / alkaloids
Hypericum / terpenoids
Taxus / alkaloids
Rhodiola / tyroso
Sedum / flavonoids
Sedum / salidroside
Sedum / phenolic compounds
PC1 (30.2% variance)
PC2 (19.0% variance)
Target evidence score
