# Supplementary material for: Integrating Biosynthetic, Genomic and Ecological Open Data for Medicinal Plant Research: A Leakage-Aware Evidence-Prioritization Framework
Source: Int J Mol Sci. 2026 Jul 29;27(15):6801. doi: 10.3390/ijms27156801 (PMC13467606; doi:10.3390/ijms27156801)
Supplement: Supplementary file 1 [file ijms-27-06801-s001.zip › med plant data revised notebooks/genetic_notebook2_outputs.zip!/figures/figure3_taxon_compound_evidence_heatmap.pdf]

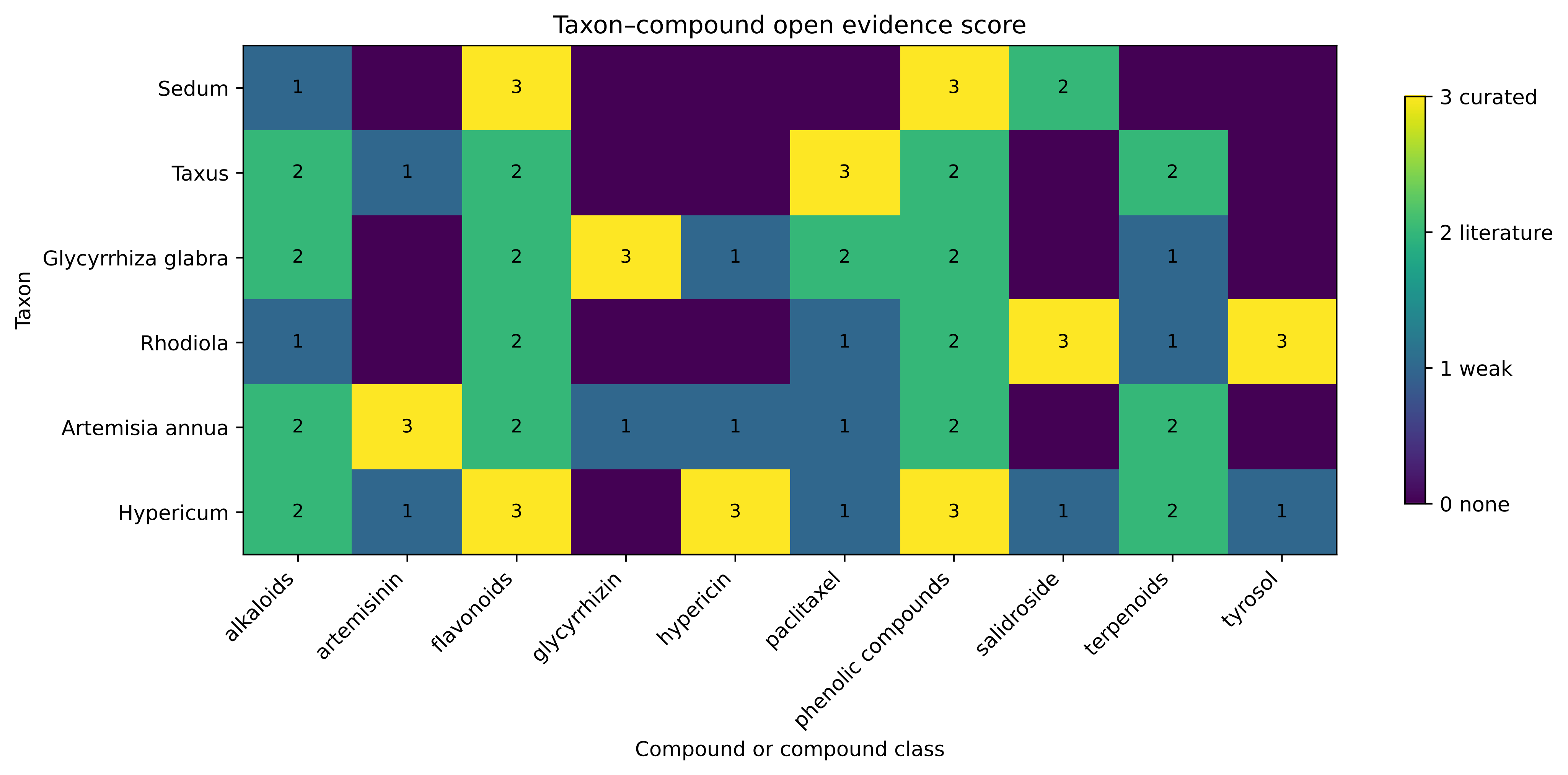
Taxon–compound open evidence score
Taxon
Sedum
Taxus
Glycyrrhiza glabra
Rhodiola
Artemisia annua
Hypericum
alkaloids
artemisinin
flavonoids
glycyrrhizin
hypericin
paclitaxel
phenolic compounds
salidroside
terpenoids
tyrosol
Compound or compound class
3 curated
2 literature
1 weak
0 none
1 3 3 2
2 1 2 3 2 2
2 2 3 1 2 2 1
1 2 1 2 3 1 3
2 3 2 1 1 1 2 2
2 1 3 3 1 3 1 2 1
