# Supplementary material for: Integrating Biosynthetic, Genomic and Ecological Open Data for Medicinal Plant Research: A Leakage-Aware Evidence-Prioritization Framework
Source: Int J Mol Sci. 2026 Jul 29;27(15):6801. doi: 10.3390/ijms27156801 (PMC13467606; doi:10.3390/ijms27156801)
Supplement: Supplementary file 1 [file ijms-27-06801-s001.zip › med plant data revised notebooks/genetic_notebook2_outputs.zip!/figures/figure6_clustered_feature_correlation_spearman.pdf]

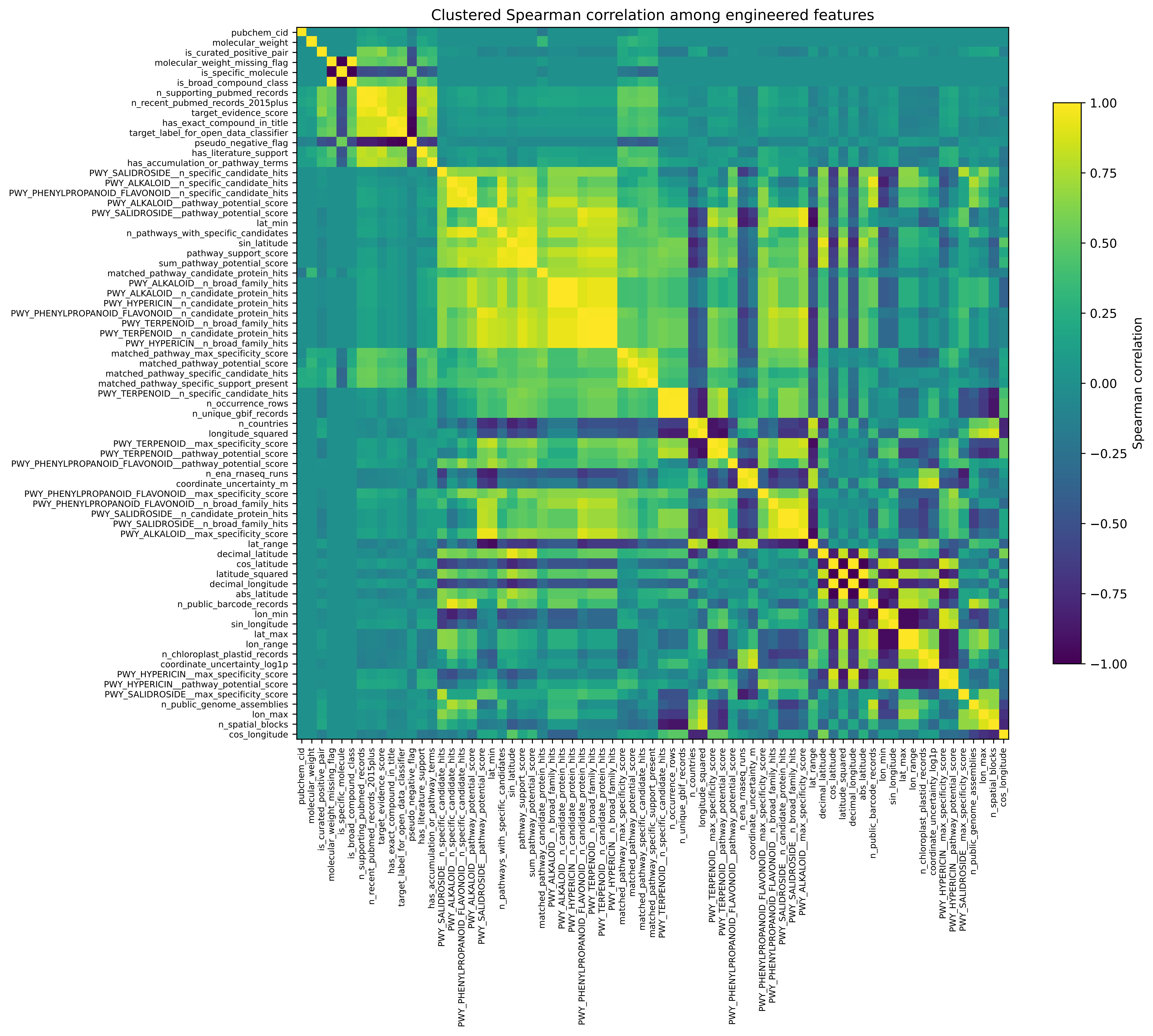
Clustered Spearman correlation among engineered features
pubchem_cid
molecular_weight
is_curated_positive_pair
molecular_weight_missing_flag
is_specific_molecule
is_broad_compound_class
n_supporting_pubmed_records
n_recent_pubmed_records_2015plus
target_evidence_score
has_exact_compound_in_title
target_label_for_open_data_classifier
pseudo_negative_flag
has_literature_support
has_accumulation_or_pathway_terms
PWY_SALIDROSIDE__n_specific_candidate_hits
PWY_ALKALOID__n_specific_candidate_hits
PWY_PHENYLPROPANOID_FLAVONOID__n_specific_candidate_hits
PWY_ALKALOID__pathway_potential_score
PWY_SALIDROSIDE__pathway_potential_score
lat_min
n_pathways_with_specific_candidates
sin_latitude
pathway_support_score
sum_pathway_potential_score
matched_pathway_candidate_protein_hits
PWY_ALKALOID__n_broad_family_hits
PWY_ALKALOID__n_candidate_protein_hits
PWY_HYPERICIN__n_candidate_protein_hits
PWY_PHENYLPROPANOID_FLAVONOID__n_candidate_protein_hits
PWY_TERPENOID__n_broad_family_hits
PWY_TERPENOID__n_candidate_protein_hits
PWY_HYPERICIN__n_broad_family_hits
matched_pathway_max_specificity_score
matched_pathway_potential_score
matched_pathway_specific_candidate_hits
matched_pathway_specific_support_present
PWY_TERPENOID__n_specific_candidate_hits
n_occurrence_rows
n_unique_gbif_records
n_countries
longitude_squared
PWY_TERPENOID__max_specificity_score
PWY_TERPENOID__pathway_potential_score
PWY_PHENYLPROPANOID_FLAVONOID__pathway_potential_score
n_ena_rnaseq_runs
coordinate_uncertainty_m
PWY_PHENYLPROPANOID_FLAVONOID__max_specificity_score
PWY_PHENYLPROPANOID_FLAVONOID__n_broad_family_hits
PWY_SALIDROSIDE__n_candidate_protein_hits
PWY_SALIDROSIDE__n_broad_family_hits
PWY_ALKALOID__max_specificity_score
lat_range
decimal_latitude
cos_latitude
latitude_squared
decimal_longitude
abs_latitude
n_public_barcode_records
lon_min
sin_longitude
lat_max
lon_range
n_chloroplast_plastid_records
coordinate_uncertainty_log1p
PWY_HYPERICIN__max_specificity_score
PWY_HYPERICIN__pathway_potential_score
PWY_SALIDROSIDE__max_specificity_score
n_public_genome_assemblies
lon_max
n_spatial_blocks
cos_longitude
1.00
0.75
0.50
0.25
0.00
−0.25
−0.50
−0.75
−1.00
Spearman correlation
