# Supplementary material for: Integrating Biosynthetic, Genomic and Ecological Open Data for Medicinal Plant Research: A Leakage-Aware Evidence-Prioritization Framework
Source: Int J Mol Sci. 2026 Jul 29;27(15):6801. doi: 10.3390/ijms27156801 (PMC13467606; doi:10.3390/ijms27156801)
Supplement: Supplementary file 1 [file ijms-27-06801-s001.zip › med plant data revised notebooks/genetic_notebook2_outputs.zip!/figures/figure5_evidence_prioritization_quadrant.pdf]

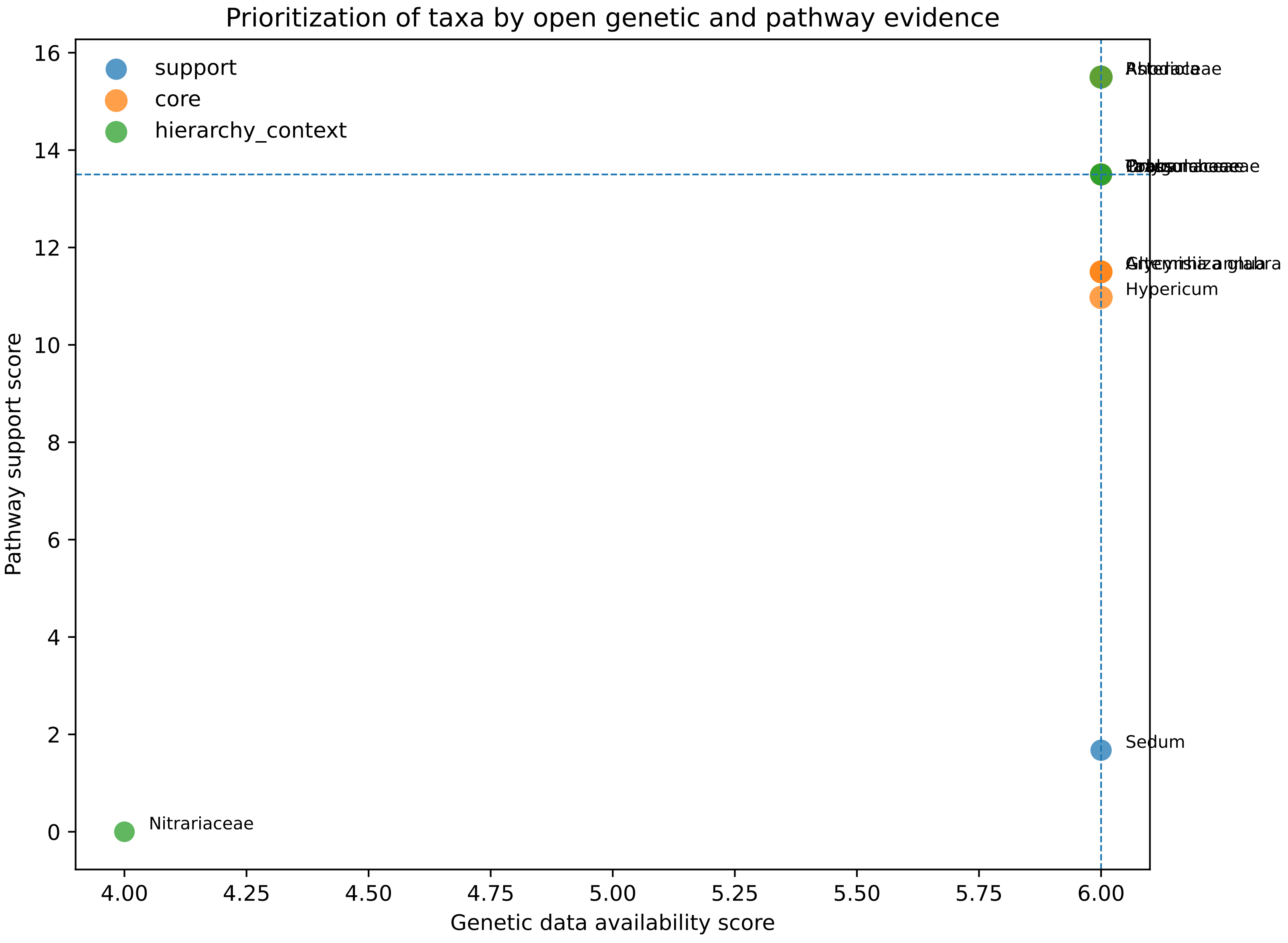
Prioritization of taxa by open genetic and pathway evidence
support
core
hierarchy_context
Asteraceae
Rhodiola
Glycyrrhiza glabra
Artemisia annua
Hypericum
Sedum
Nitrariaceae
Pathway support score
Genetic data availability score
16
14
12
10
8
6
4
2
0
4.00
4.25
4.50
4.75
5.00
5.25
5.50
5.75
6.00
