# Supplementary material for: Integrating Biosynthetic, Genomic and Ecological Open Data for Medicinal Plant Research: A Leakage-Aware Evidence-Prioritization Framework
Source: Int J Mol Sci. 2026 Jul 29;27(15):6801. doi: 10.3390/ijms27156801 (PMC13467606; doi:10.3390/ijms27156801)
Supplement: Supplementary file 1 [file ijms-27-06801-s001.zip › med plant data revised notebooks/genetic_notebook2_outputs.zip!/figures/figure1_multilayer_open_data_coverage_by_taxon.pdf]

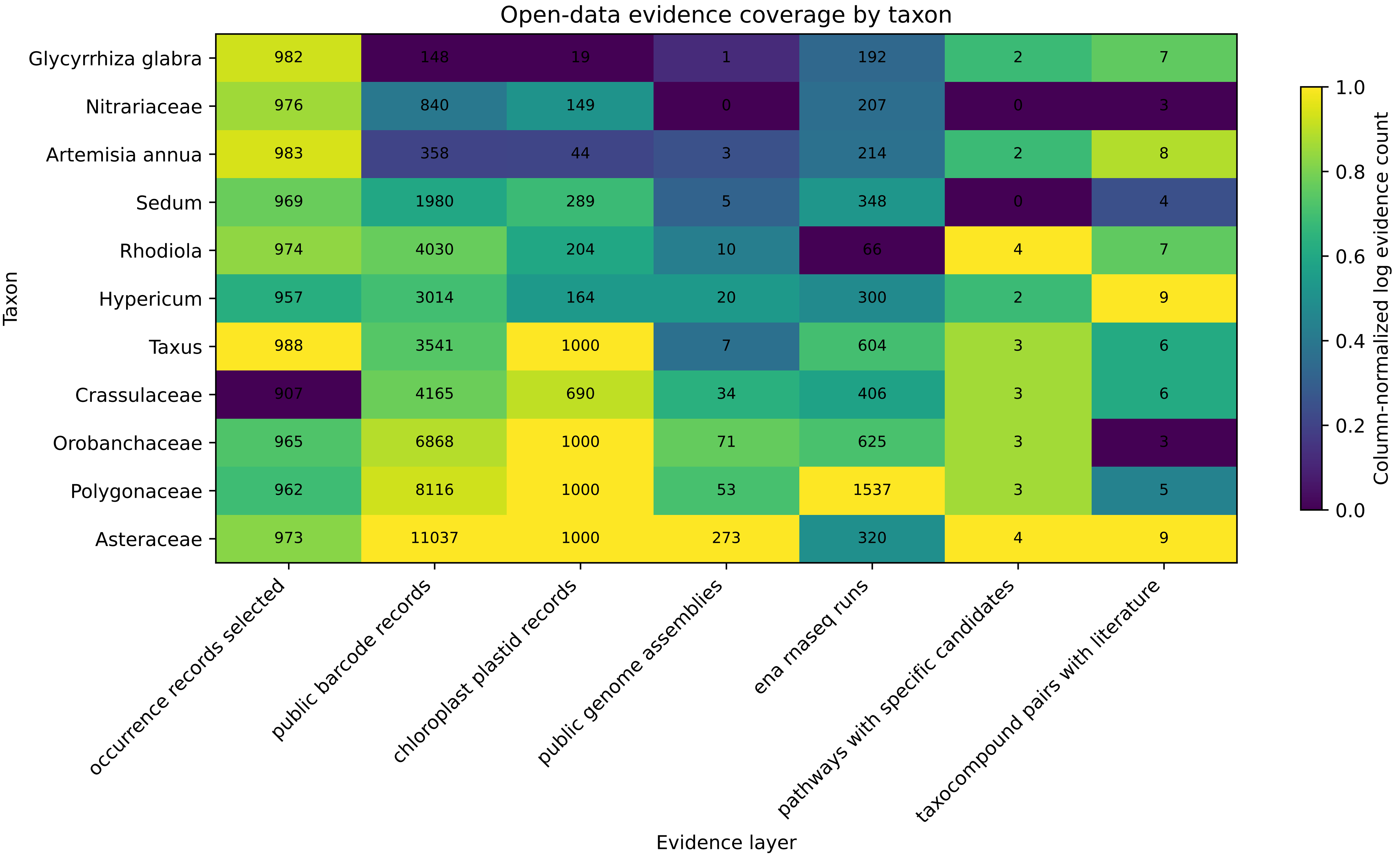

Open-data evidence coverage by taxon
Taxon
Glycyrrhiza glabra
Nitrariaceae
Artemisia annua
Sedum
Rhodiola
Hypericum
Taxus
Crassulaceae
Orobanchaceae
Polygonaceae
Asteraceae
Evidence layer
occurrence records selected
public barcode records
chloroplast plastid records
public genome assemblies
ena rnaseq runs
pathways with specific candidates
taxocompound pairs with literature
Column-normalized log evidence count
982 148 19 1 192 2 7
976 840 149 0 207 0 3
983 358 44 3 214 2 8
969 1980 289 5 348 0 4
974 4030 204 10 66 4 7
957 3014 164 20 300 2 9
988 3541 1000 7 604 3 6
907 4165 690 34 406 3 6
965 6868 1000 71 625 3 3
962 8116 1000 53 1537 3 5
973 11037 1000 273 320 4 9
0.0
0.2
0.4
0.6
0.8
1.0
