# Supplementary material for: Integrating Biosynthetic, Genomic and Ecological Open Data for Medicinal Plant Research: A Leakage-Aware Evidence-Prioritization Framework
Source: Int J Mol Sci. 2026 Jul 29;27(15):6801. doi: 10.3390/ijms27156801 (PMC13467606; doi:10.3390/ijms27156801)
Supplement: Supplementary file 1 [file ijms-27-06801-s001.zip › med plant data revised notebooks/genetic_notebook2_outputs.zip!/figures/figure2_global_occurrence_space.pdf]

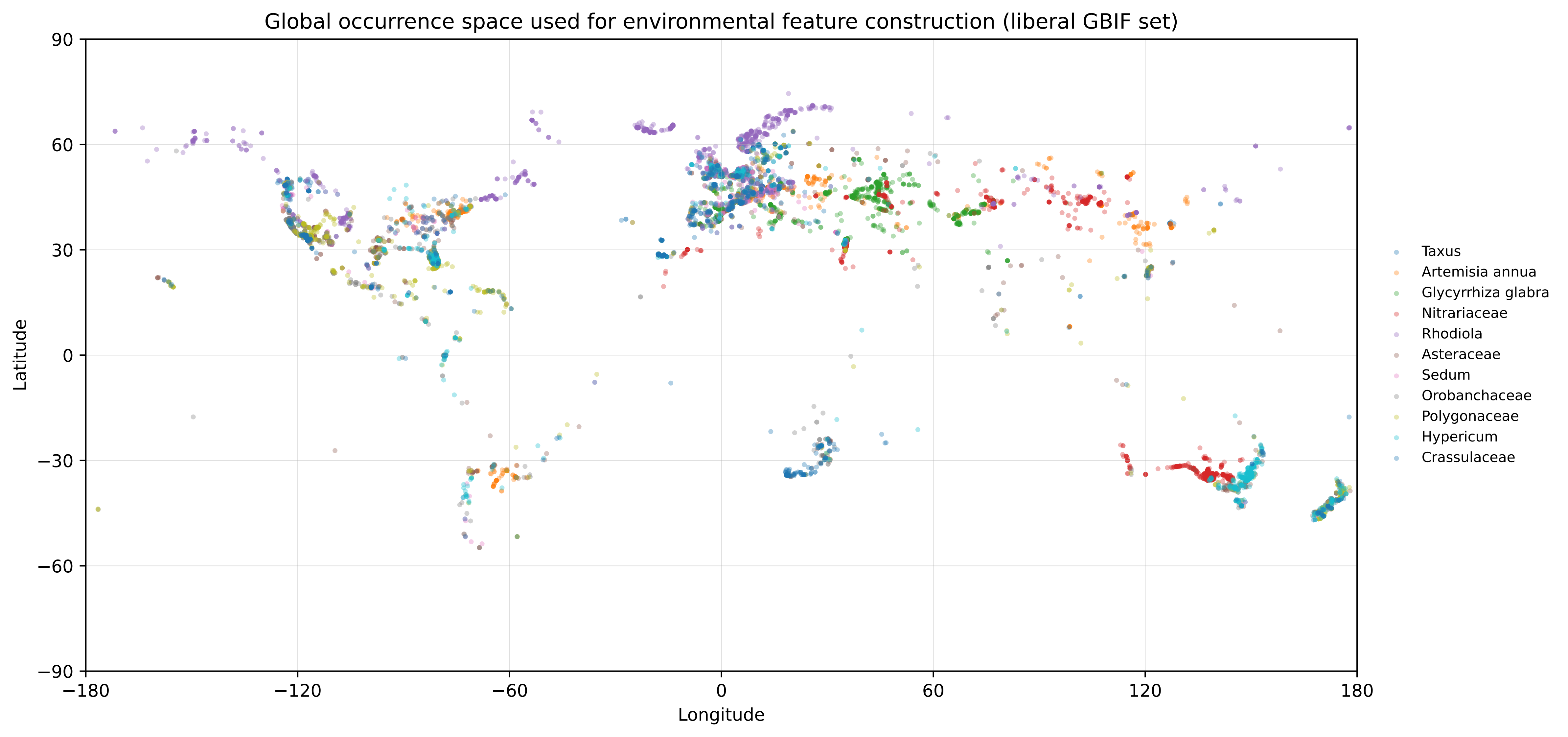

Global occurrence space used for environmental feature construction (liberal GBIF set)
90
60
30
0
−30
−60
−90
Latitude
−180
−120
−60
0
60
120
180
Longitude
Taxus
Artemisia annua
Glycyrrhiza glabra
Nitrariaceae
Rhodiola
Asteraceae
Sedum
Orobanchaceae
Polygonaceae
Hypericum
Crassulaceae
