# Supplementary material for: Integrating Biosynthetic, Genomic and Ecological Open Data for Medicinal Plant Research: A Leakage-Aware Evidence-Prioritization Framework
Source: Int J Mol Sci. 2026 Jul 29;27(15):6801. doi: 10.3390/ijms27156801 (PMC13467606; doi:10.3390/ijms27156801)
Supplement: Supplementary file 1 [file ijms-27-06801-s001.zip › med plant data revised notebooks/genetic_notebook2_outputs.zip!/figures/figure4_pathway_potential_heatmap.pdf]

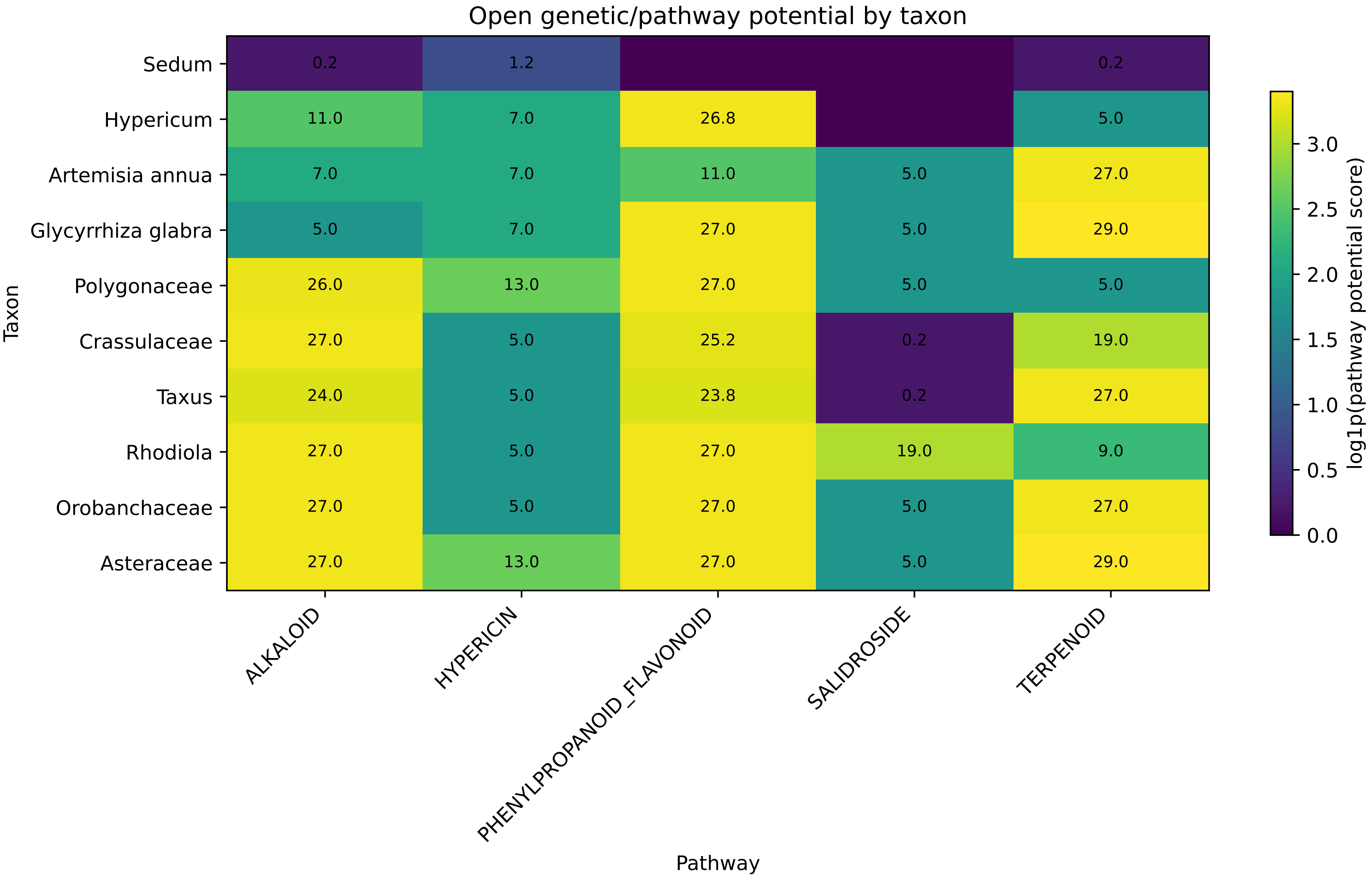

Open genetic/pathway potential by taxon
Taxon
Sedum
Hypericum
Artemisia annua
Glycyrrhiza glabra
Polygonaceae
Crassulaceae
Taxus
Rhodiola
Orobanchaceae
Asteraceae
Pathway
ALKALOID
HYPERICIN
PHENYLPROPANOID_FLAVONOID
SALIDROSIDE
TERPENOID
0.2
1.2
0.2
11.0
7.0
26.8
5.0
7.0
7.0
11.0
5.0
27.0
5.0
7.0
27.0
5.0
29.0
26.0
13.0
27.0
5.0
5.0
27.0
5.0
25.2
0.2
19.0
24.0
5.0
23.8
0.2
27.0
27.0
5.0
27.0
19.0
9.0
27.0
5.0
27.0
5.0
27.0
27.0
13.0
27.0
5.0
29.0
log1p(pathway potential score)
0.0
0.5
1.0
1.5
2.0
2.5
3.0
