# Supplementary material for: Integrating Biosynthetic, Genomic and Ecological Open Data for Medicinal Plant Research: A Leakage-Aware Evidence-Prioritization Framework
Source: Int J Mol Sci. 2026 Jul 29;27(15):6801. doi: 10.3390/ijms27156801 (PMC13467606; doi:10.3390/ijms27156801)
Supplement: Supplementary file 1 [file ijms-27-06801-s001.zip › med plant data revised notebooks/genetic_notebook2_outputs.zip!/figures/figure9_environmental_space_pca_by_occurrence.pdf]

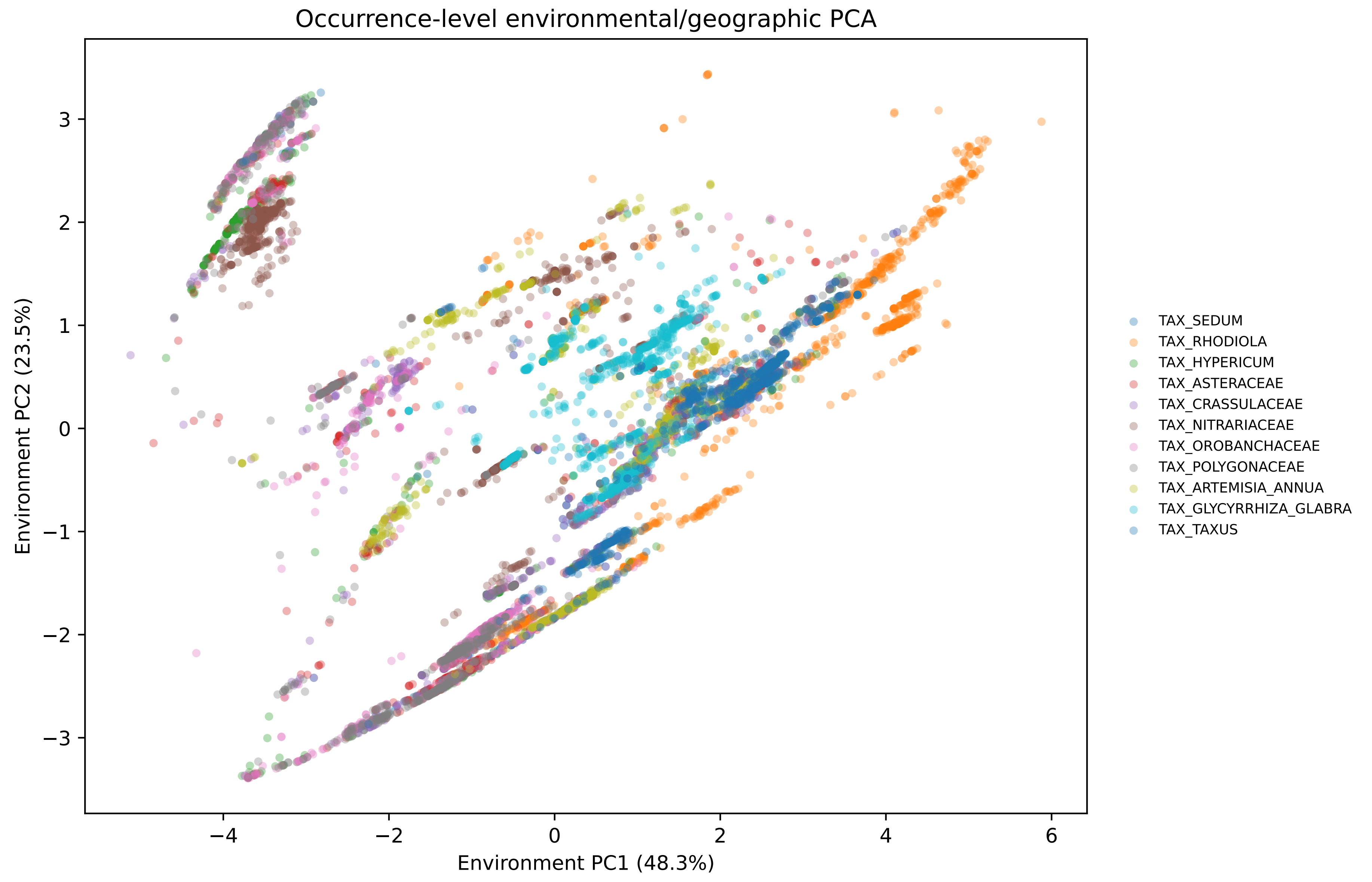
Occurrence-level environmental/geographic PCA
Environment PC1 (48.3%)
Environment PC2 (23.5%)
−4
−2
0
2
4
6
−3
−2
−1
0
1
2
3
TAX_SEDUM
TAX_RHODIOLA
TAX_HYPERICUM
TAX_ASTERACEAE
TAX_CRASSULACEAE
TAX_NITRARIACEAE
TAX_OROBANCHACEAE
TAX_POLYGONACEAE
TAX_ARTEMISIA_ANNUA
TAX_GLYCYRRHIZA_GLABRA
TAX_TAXUS
