# Supplementary material for: Integrating Biosynthetic, Genomic and Ecological Open Data for Medicinal Plant Research: A Leakage-Aware Evidence-Prioritization Framework
Source: Int J Mol Sci. 2026 Jul 29;27(15):6801. doi: 10.3390/ijms27156801 (PMC13467606; doi:10.3390/ijms27156801)
Supplement: Supplementary file 1 [file ijms-27-06801-s001.zip › med plant data revised notebooks/genetic_notebook3_outputs.zip!/figures/figure2E_selected_primary_model_confusion_matrix_v4.pdf]

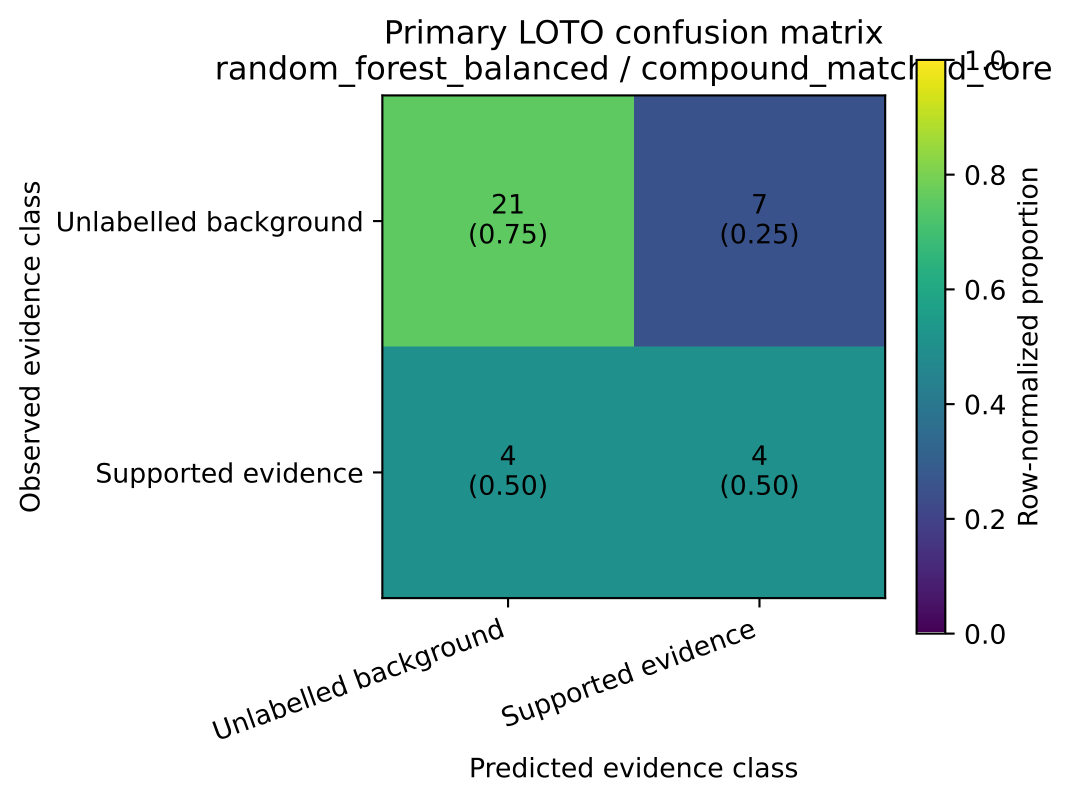

Primary LOTO confusion matrix
random_forest_balanced / compound_matc d_core
Observed evidence class
Unlabelled background
Supported evidence
21
(0.75)
7
(0.25)
4
(0.50)
4
(0.50)
Predicted evidence class
Unlabelled background
Supported evidence
Row-normalized proportion
0.0
0.2
0.4
0.6
0.8
1.0
