# Supplementary material for: Integrating Biosynthetic, Genomic and Ecological Open Data for Medicinal Plant Research: A Leakage-Aware Evidence-Prioritization Framework
Source: Int J Mol Sci. 2026 Jul 29;27(15):6801. doi: 10.3390/ijms27156801 (PMC13467606; doi:10.3390/ijms27156801)
Supplement: Supplementary file 1 [file ijms-27-06801-s001.zip › med plant data revised notebooks/genetic_notebook3_outputs.zip!/figures/figure7_evidence_graph_leakage_safe_prioritization_v2.pdf]

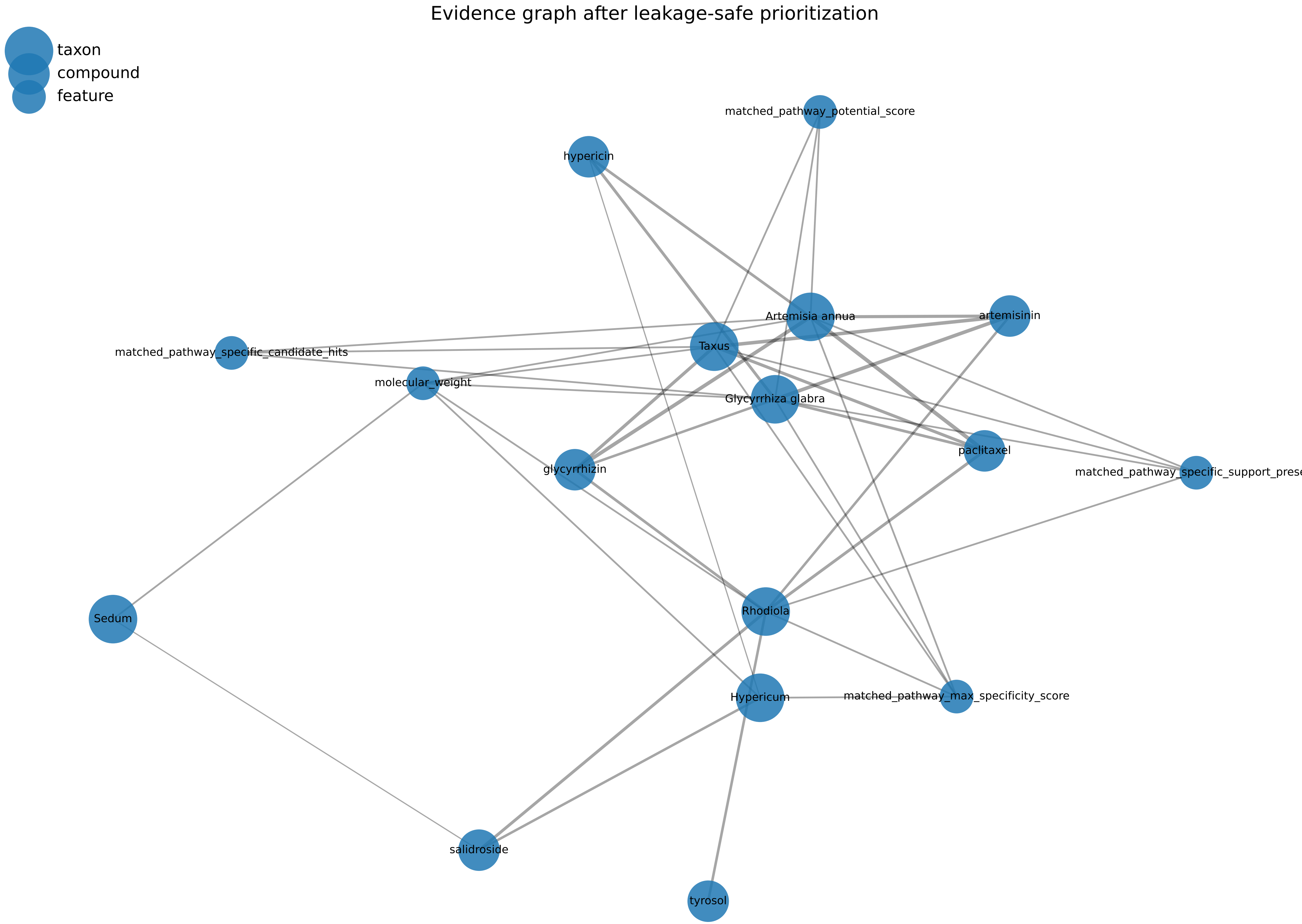
Evidence graph after leakage-safe prioritization
taxon
compound
feature
matched_pathway_potential_score
hypericin
Artemisia annua
artemisinin
Taxus
matched_pathway_specific_candidate_hits
molecular_weight
Glycyrrhiza glabra
paclitaxel
glycyrrhizin
matched_pathway_specific_support_preser
Sedum
Rhodiola
Hypericum
matched_pathway_max_specificity_score
salidroside
tyrosol
