# Supplementary material for: Integrating Biosynthetic, Genomic and Ecological Open Data for Medicinal Plant Research: A Leakage-Aware Evidence-Prioritization Framework
Source: Int J Mol Sci. 2026 Jul 29;27(15):6801. doi: 10.3390/ijms27156801 (PMC13467606; doi:10.3390/ijms27156801)
Supplement: Supplementary file 1 [file ijms-27-06801-s001.zip › med plant data revised notebooks/genetic_notebook3_outputs.zip!/figures/figure2_leakage_safe_performance_and_controls_v2.pdf]

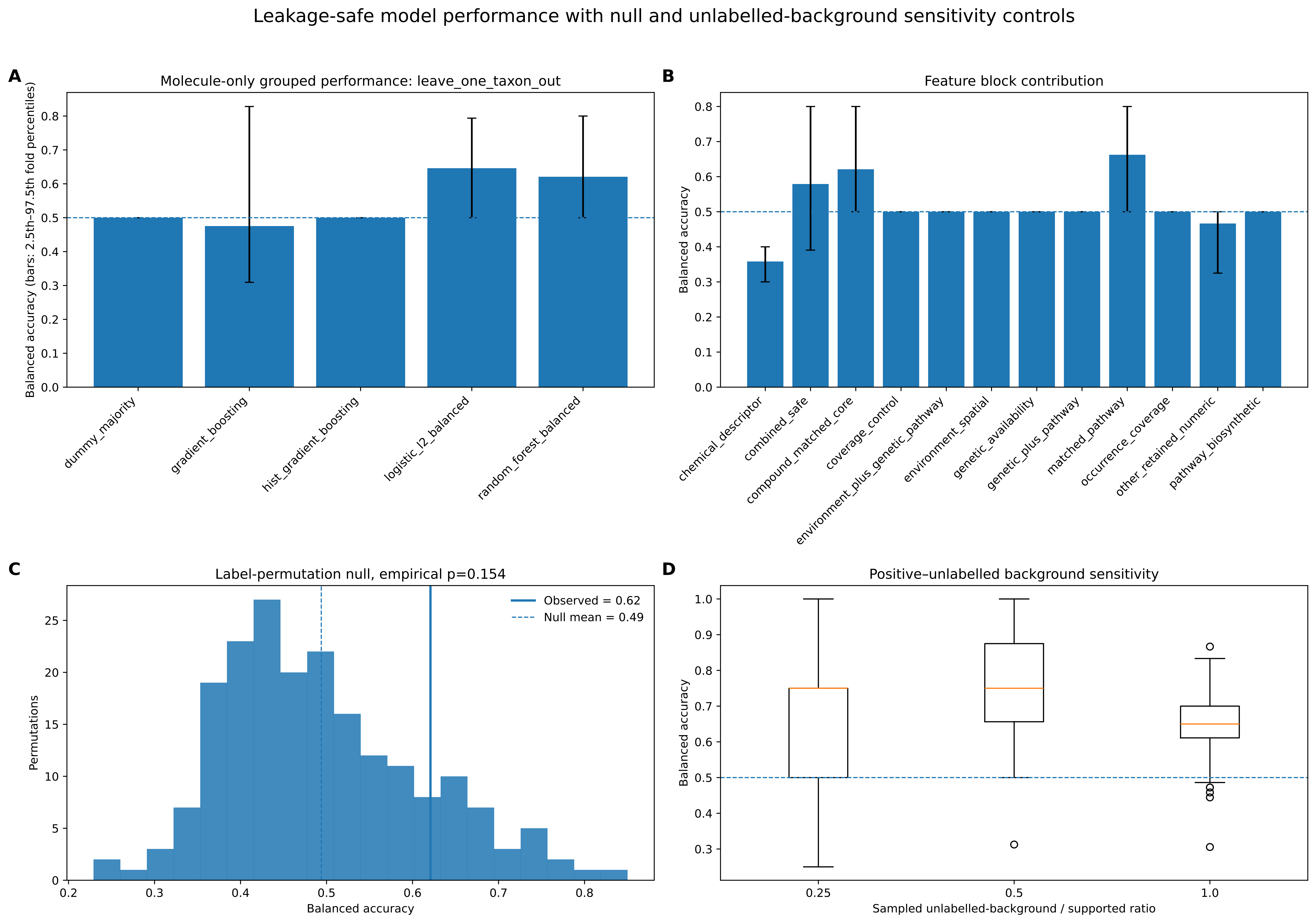

Leakage-safe model performance with null and unlabelled-background sensitivity controls
A
Molecule-only grouped performance: leave_one_taxon_out
Balanced accuracy (bars: 2.5th–97.5th fold percentiles)
dummy_majority
gradient_boosting
hist_gradient_boosting
logistic_l2_balanced
random_forest_balanced
B
Feature block contribution
Balanced accuracy
chemical_descriptor
combined_safe
compound_matched_core
coverage_control
environment_plus_genetic_pathway
environment_spatial
genetic_availability
genetic_plus_pathway
matched_pathway
occurrence_coverage
other_retained_numeric
pathway_biosynthetic
C
Label-permutation null, empirical p=0.154
Observed = 0.62
Null mean = 0.49
Permutations
Balanced accuracy
D
Positive–unlabelled background sensitivity
Balanced accuracy
Sampled unlabelled-background / supported ratio
