# Supplementary material for: Integrating Biosynthetic, Genomic and Ecological Open Data for Medicinal Plant Research: A Leakage-Aware Evidence-Prioritization Framework
Source: Int J Mol Sci. 2026 Jul 29;27(15):6801. doi: 10.3390/ijms27156801 (PMC13467606; doi:10.3390/ijms27156801)
Supplement: Supplementary file 1 [file ijms-27-06801-s001.zip › med plant data revised notebooks/genetic_notebook3_outputs.zip!/figures/figure1_corrected_dataset_design_and_leakage_audit_v2.pdf]

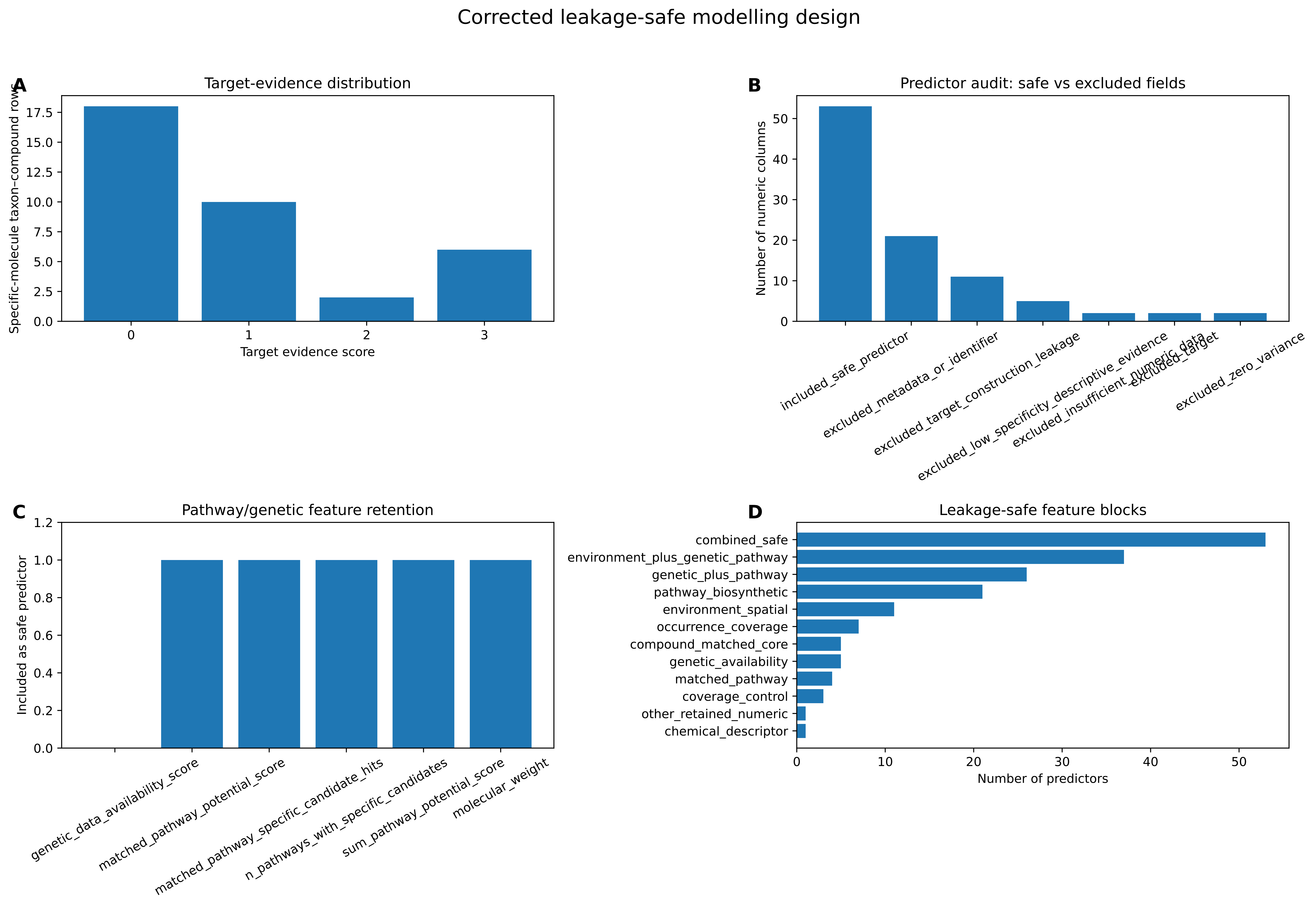
Corrected leakage-safe modelling design
A
Target-evidence distribution
Specific-molecule taxon–compound rows
Target evidence score
0
1
2
3
0.0
2.5
5.0
7.5
10.0
12.5
15.0
17.5
B
Predictor audit: safe vs excluded fields
Number of numeric columns
0
10
20
30
40
50
included_safe_predictor
excluded_metadata_or_identifier
excluded_target_construction_leakage
excluded_low_specificity_descriptive_evidence
excluded_insufficient_numeric_data
excluded_target
excluded_zero_variance
C
Pathway/genetic feature retention
Included as safe predictor
0.0
0.2
0.4
0.6
0.8
1.0
1.2
genetic_data_availability_score
matched_pathway_potential_score
matched_pathway_specific_candidate_hits
n_pathways_with_specific_candidates
sum_pathway_potential_score
molecular_weight
D
Leakage-safe feature blocks
combined_safe
environment_plus_genetic_pathway
genetic_plus_pathway
pathway_biosynthetic
environment_spatial
occurrence_coverage
compound_matched_core
genetic_availability
matched_pathway
coverage_control
other_retained_numeric
chemical_descriptor
0
10
20
30
40
50
Number of predictors
