# Supplementary material for: Integrating Biosynthetic, Genomic and Ecological Open Data for Medicinal Plant Research: A Leakage-Aware Evidence-Prioritization Framework
Source: Int J Mol Sci. 2026 Jul 29;27(15):6801. doi: 10.3390/ijms27156801 (PMC13467606; doi:10.3390/ijms27156801)
Supplement: Supplementary file 1 [file ijms-27-06801-s001.zip › med plant data revised notebooks/soilgrids_notebook1_outputs.zip!/figures/figure2_soilgrids_feature_completeness_profiles_v3.pdf]

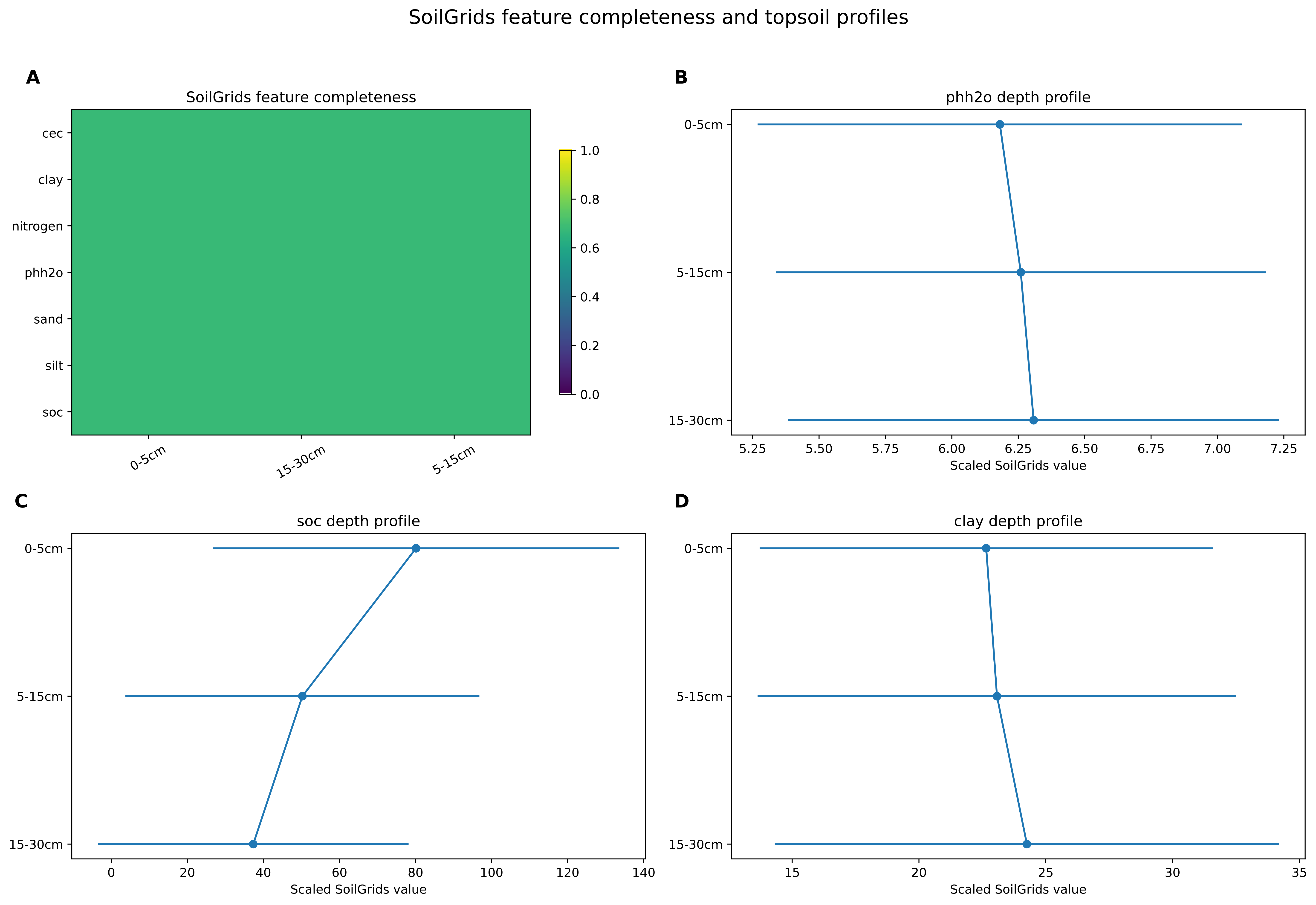

SoilGrids feature completeness and topsoil profiles
A
SoilGrids feature completeness
cec
clay
nitrogen
phh2o
sand
silt
soc
0-5cm
15-30cm
5-15cm
0.0
0.2
0.4
0.6
0.8
1.0
B
phh2o depth profile
0-5cm
5-15cm
15-30cm
5.25
5.50
5.75
6.00
6.25
6.50
6.75
7.00
7.25
Scaled SoilGrids value
C
soc depth profile
0-5cm
5-15cm
15-30cm
0
20
40
60
80
100
120
140
Scaled SoilGrids value
D
clay depth profile
0-5cm
5-15cm
15-30cm
15
20
25
30
35
Scaled SoilGrids value
