# Supplementary material for: Integrating Biosynthetic, Genomic and Ecological Open Data for Medicinal Plant Research: A Leakage-Aware Evidence-Prioritization Framework
Source: Int J Mol Sci. 2026 Jul 29;27(15):6801. doi: 10.3390/ijms27156801 (PMC13467606; doi:10.3390/ijms27156801)
Supplement: Supplementary file 1 [file ijms-27-06801-s001.zip › med plant data revised notebooks/soilgrids_notebook2_outputs.zip!/figures/figure3_soilgrids_correlation_pca_relationships.pdf]

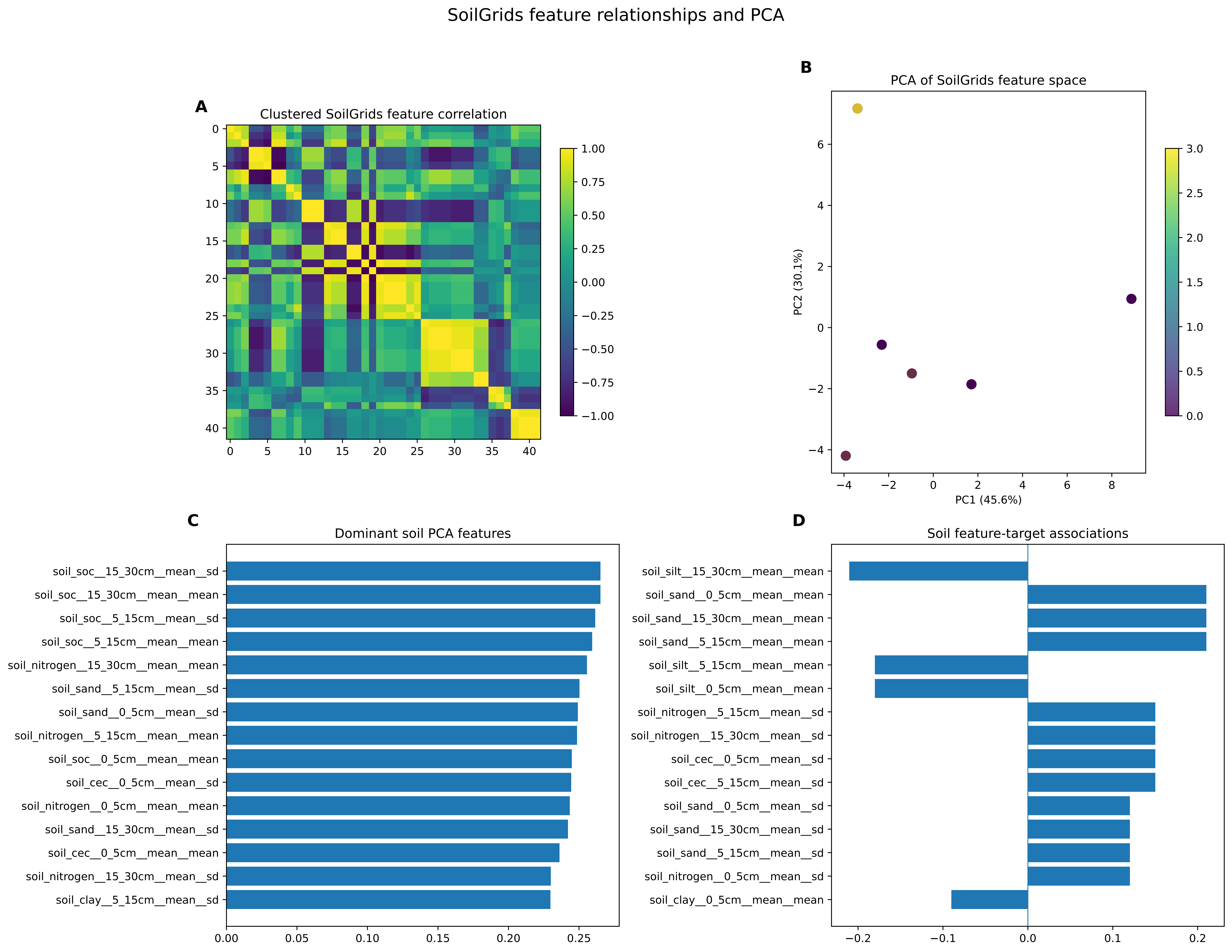

SoilGrids feature relationships and PCA
A
Clustered SoilGrids feature correlation
B
PCA of SoilGrids feature space
PC1 (45.6%)
PC2 (30.1%)
C
Dominant soil PCA features
soil_soc__15_30cm__mean__sd
soil_soc__15_30cm__mean__mean
soil_soc__5_15cm__mean__sd
soil_soc__5_15cm__mean__mean
soil_nitrogen__15_30cm__mean__mean
soil_sand__5_15cm__mean__sd
soil_sand__0_5cm__mean__sd
soil_nitrogen__5_15cm__mean__mean
soil_soc__0_5cm__mean__mean
soil_cec__0_5cm__mean__sd
soil_nitrogen__0_5cm__mean__mean
soil_sand__15_30cm__mean__sd
soil_cec__0_5cm__mean__mean
soil_nitrogen__15_30cm__mean__sd
soil_clay__5_15cm__mean__sd
D
Soil feature-target associations
soil_silt__15_30cm__mean__mean
soil_sand__0_5cm__mean__mean
soil_sand__15_30cm__mean__mean
soil_sand__5_15cm__mean__mean
soil_silt__5_15cm__mean__mean
soil_silt__0_5cm__mean__mean
soil_nitrogen__5_15cm__mean__sd
soil_nitrogen__15_30cm__mean__sd
soil_cec__0_5cm__mean__sd
soil_cec__5_15cm__mean__sd
soil_sand__0_5cm__mean__sd
soil_sand__15_30cm__mean__sd
soil_sand__5_15cm__mean__sd
soil_nitrogen__0_5cm__mean__sd
soil_clay__0_5cm__mean__mean
