# Supplementary material for: Integrating Biosynthetic, Genomic and Ecological Open Data for Medicinal Plant Research: A Leakage-Aware Evidence-Prioritization Framework
Source: Int J Mol Sci. 2026 Jul 29;27(15):6801. doi: 10.3390/ijms27156801 (PMC13467606; doi:10.3390/ijms27156801)
Supplement: Supplementary file 1 [file ijms-27-06801-s001.zip › med plant data revised notebooks/soilgrids_notebook2_outputs.zip!/figures/figure2_soilgrids_depth_profiles.pdf]

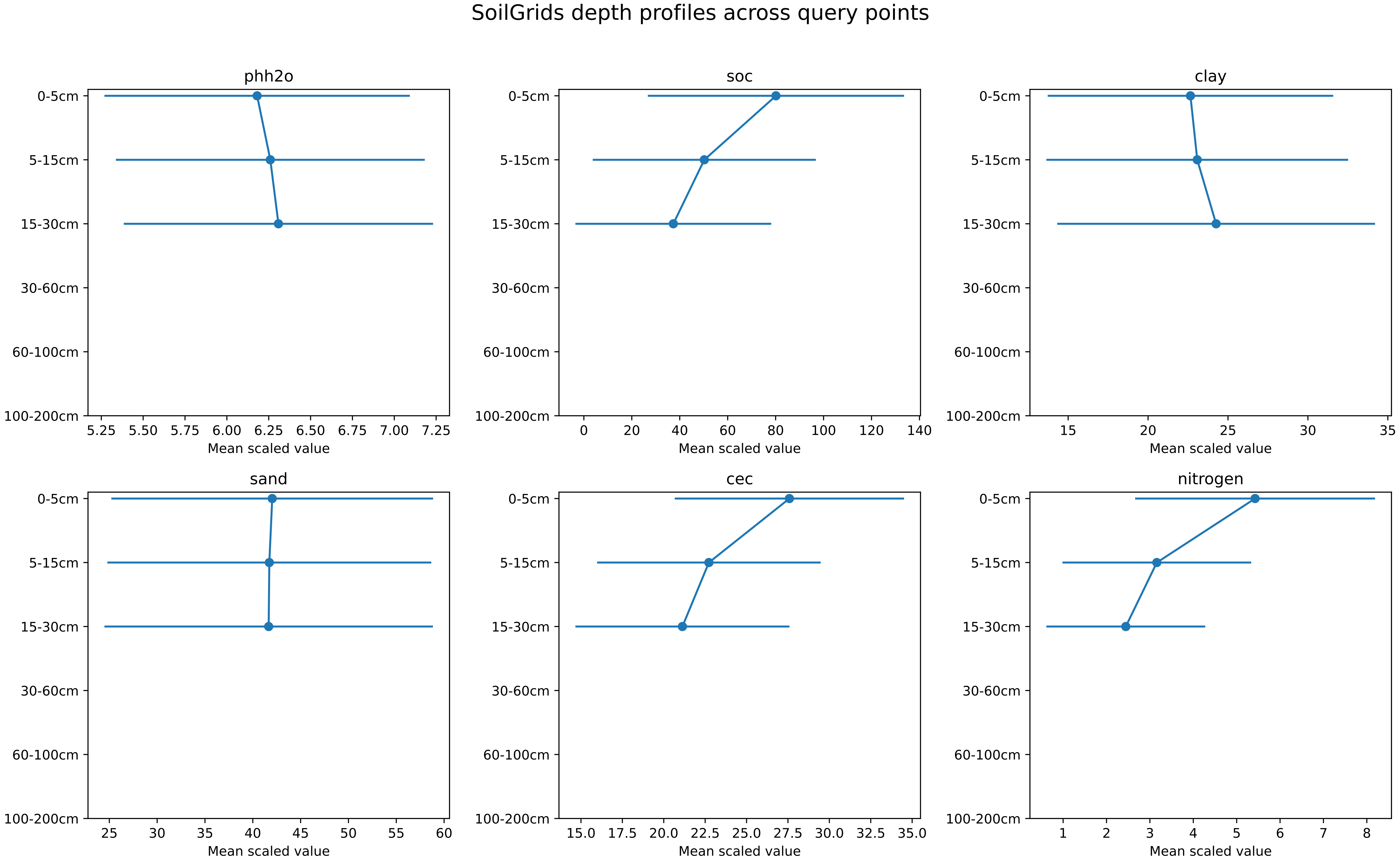

SoilGrids depth profiles across query points
phh2o
soc
clay
sand
cec
nitrogen
0-5cm
5-15cm
15-30cm
30-60cm
60-100cm
100-200cm
5.25 5.50 5.75 6.00 6.25 6.50 6.75 7.00 7.25
0 20 40 60 80 100 120 140
15 20 25 30 35
25 30 35 40 45 50 55 60
15.0 17.5 20.0 22.5 25.0 27.5 30.0 32.5 35.0
1 2 3 4 5 6 7 8
Mean scaled value
