# Supplementary material for: Integrating Biosynthetic, Genomic and Ecological Open Data for Medicinal Plant Research: A Leakage-Aware Evidence-Prioritization Framework
Source: Int J Mol Sci. 2026 Jul 29;27(15):6801. doi: 10.3390/ijms27156801 (PMC13467606; doi:10.3390/ijms27156801)

SoilGrids medicinal-plant dataset design

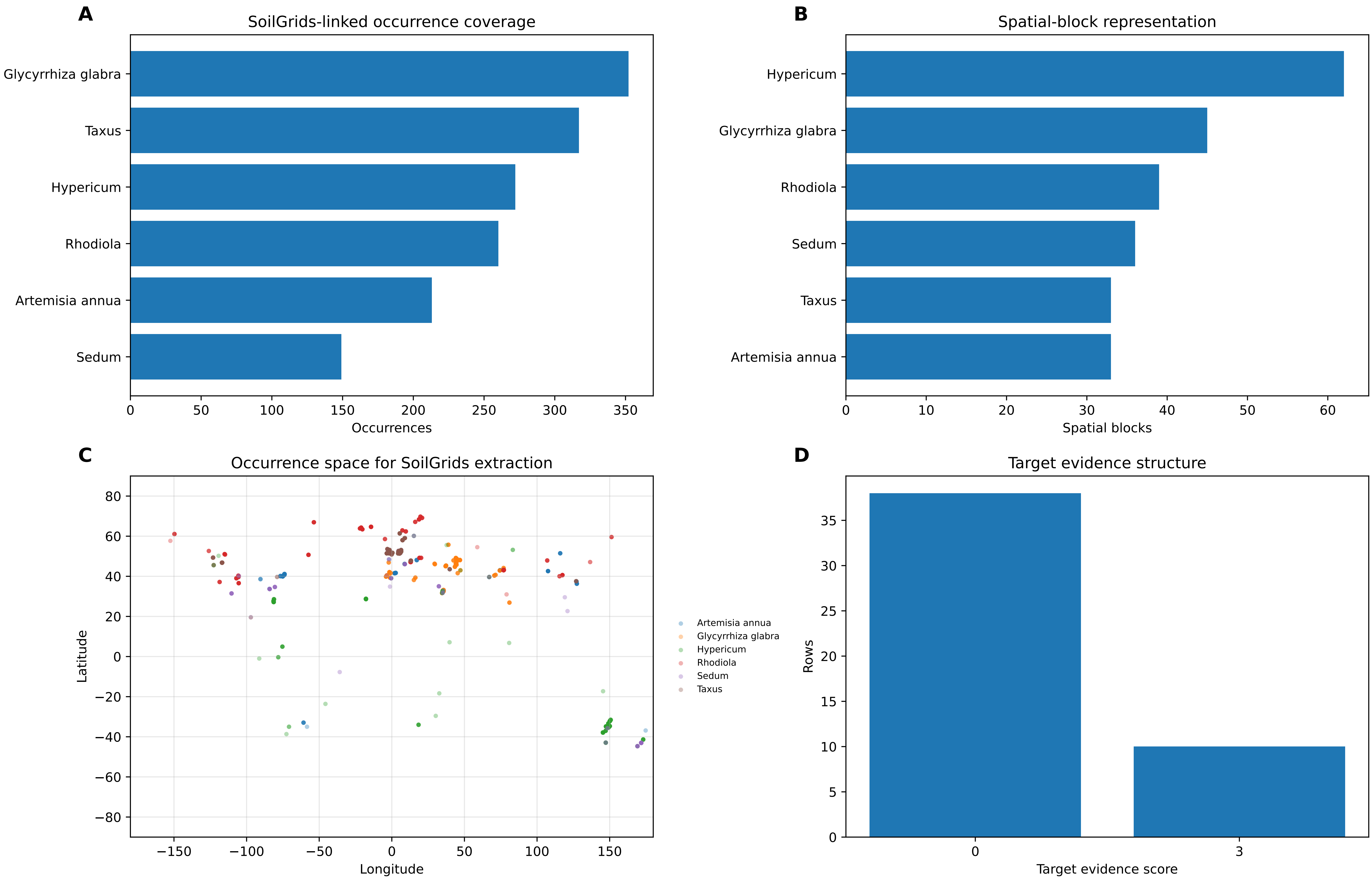

Supplement: Supplementary file 1 [file ijms-27-06801-s001.zip › med plant data revised notebooks/soilgrids_notebook2_outputs.zip!/figures/figure1_soilgrids_dataset_design.pdf]
