# Supplementary material for: Integrating Biosynthetic, Genomic and Ecological Open Data for Medicinal Plant Research: A Leakage-Aware Evidence-Prioritization Framework
Source: Int J Mol Sci. 2026 Jul 29;27(15):6801. doi: 10.3390/ijms27156801 (PMC13467606; doi:10.3390/ijms27156801)
Supplement: Supplementary file 1 [file ijms-27-06801-s001.zip › med plant data revised notebooks/soilgrids_notebook3_output.zip!/figures/figure5A_soil_pca_confidence_ellipses_permanova.pdf]

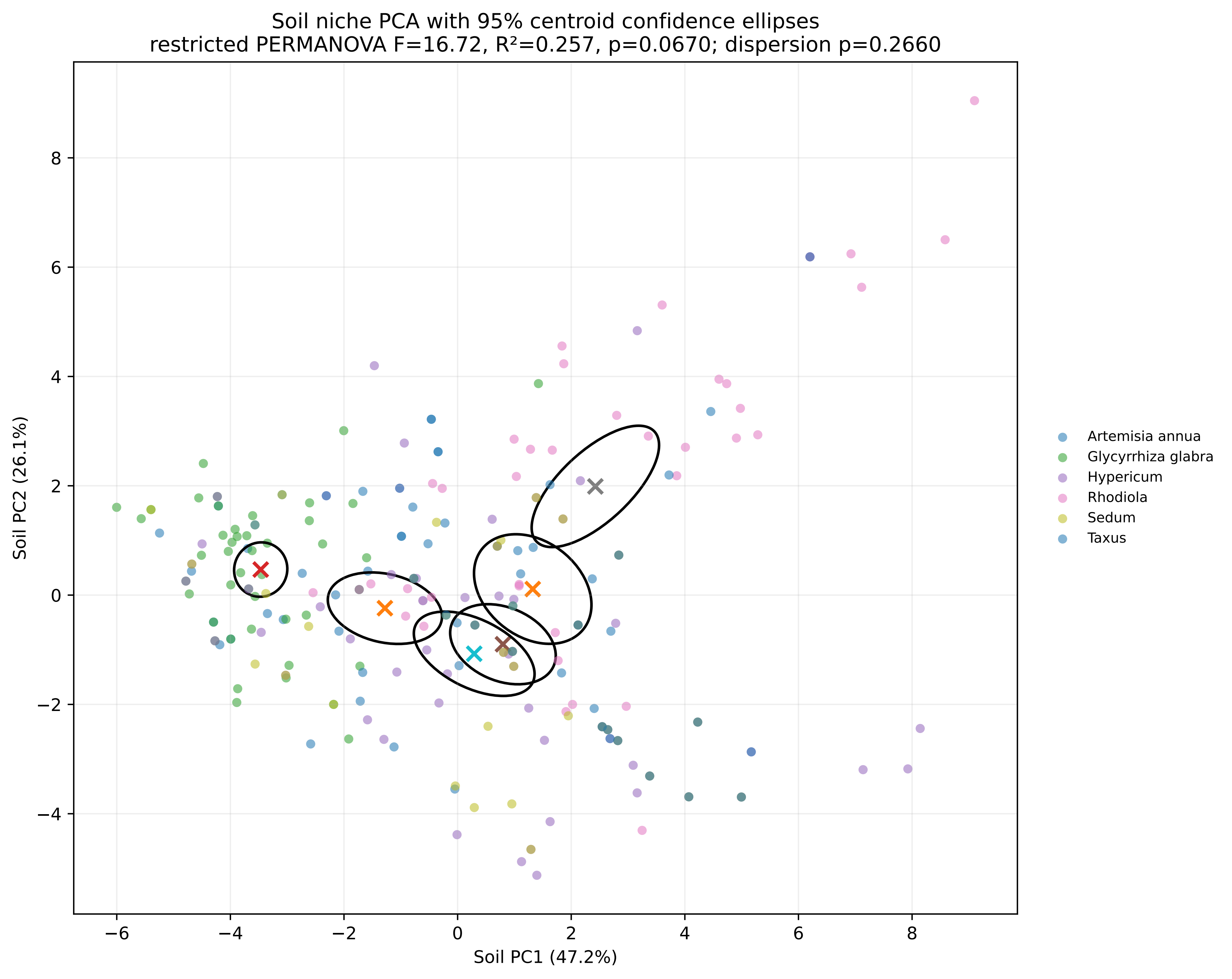
Soil niche PCA with 95% centroid confidence ellipses
restricted PERMANOVA F=16.72, R²=0.257, p=0.0670; dispersion p=0.2660
Soil PC2 (26.1%)
Soil PC1 (47.2%)
8
6
4
2
0
−2
−4
−6
−4
−2
0
2
4
6
8
Artemisia annua
Glycyrrhiza glabra
Hypericum
Rhodiola
Sedum
Taxus
