# Supplementary material for: Integrating Biosynthetic, Genomic and Ecological Open Data for Medicinal Plant Research: A Leakage-Aware Evidence-Prioritization Framework
Source: Int J Mol Sci. 2026 Jul 29;27(15):6801. doi: 10.3390/ijms27156801 (PMC13467606; doi:10.3390/ijms27156801)
Supplement: Supplementary file 1 [file ijms-27-06801-s001.zip › med plant data revised notebooks/soilgrids_notebook3_output.zip!/figures/figure2_soil_niche_pca_feature_structure.pdf]

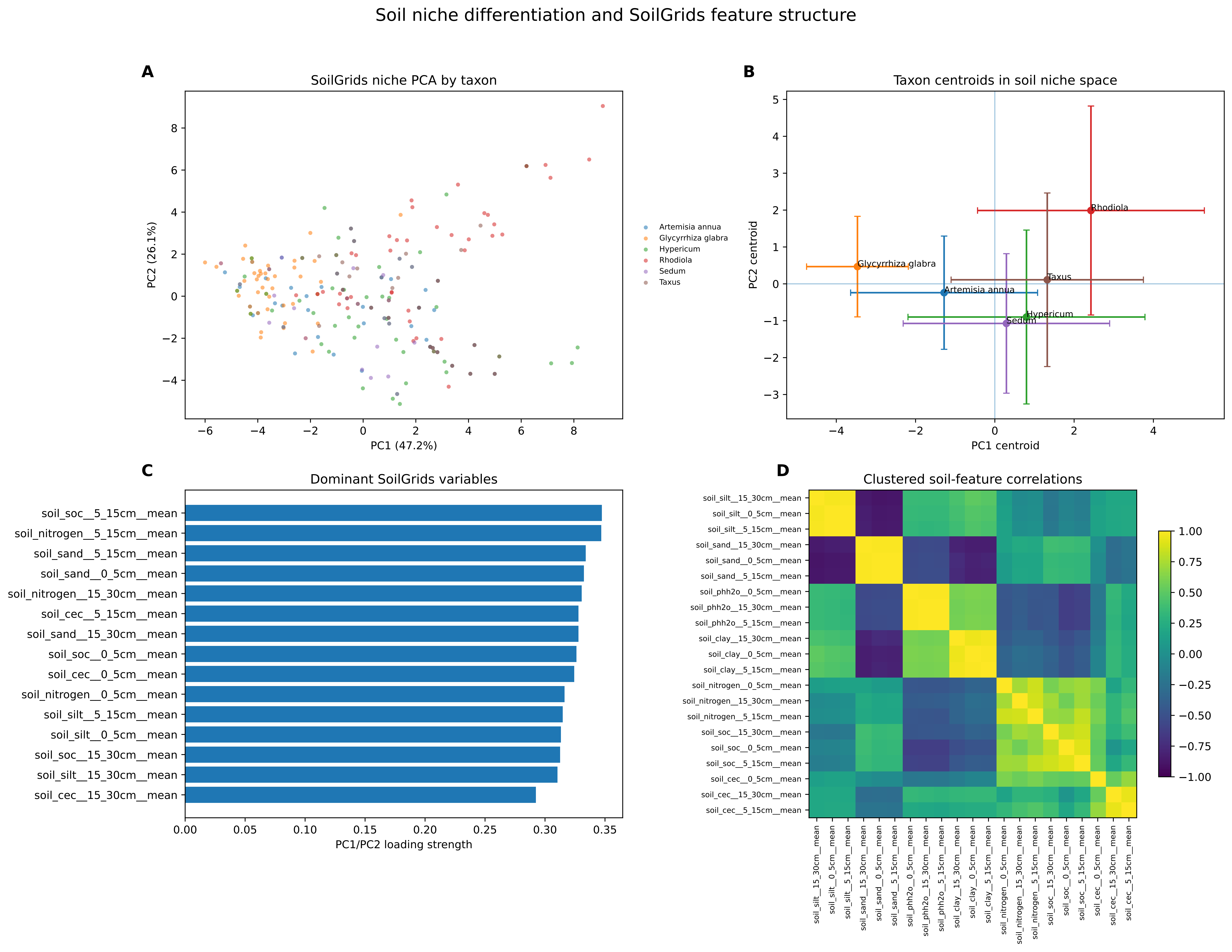

Soil niche differentiation and SoilGrids feature structure
A
SoilGrids niche PCA by taxon
PC1 (47.2%)
PC2 (26.1%)
Artemisia annua
Glycyrrhiza glabra
Hypericum
Rhodiola
Sedum
Taxus
B
Taxon centroids in soil niche space
PC1 centroid
PC2 centroid
C
Dominant SoilGrids variables
soil_soc__5_15cm__mean
soil_nitrogen__5_15cm__mean
soil_sand__5_15cm__mean
soil_sand__0_5cm__mean
soil_nitrogen__15_30cm__mean
soil_cec__5_15cm__mean
soil_sand__15_30cm__mean
soil_soc__0_5cm__mean
soil_cec__0_5cm__mean
soil_nitrogen__0_5cm__mean
soil_silt__5_15cm__mean
soil_silt__0_5cm__mean
soil_soc__15_30cm__mean
soil_silt__15_30cm__mean
soil_cec__15_30cm__mean
PC1/PC2 loading strength
D
Clustered soil-feature correlations
soil_silt__15_30cm__mean
soil_silt__0_5cm__mean
soil_silt__5_15cm__mean
soil_sand__15_30cm__mean
soil_sand__0_5cm__mean
soil_sand__5_15cm__mean
soil_phh2o__0_5cm__mean
soil_phh2o__15_30cm__mean
soil_phh2o__5_15cm__mean
soil_clay__15_30cm__mean
soil_clay__0_5cm__mean
soil_clay__5_15cm__mean
soil_nitrogen__0_5cm__mean
soil_nitrogen__15_30cm__mean
soil_nitrogen__5_15cm__mean
soil_soc__15_30cm__mean
soil_soc__0_5cm__mean
soil_soc__5_15cm__mean
soil_cec__0_5cm__mean
soil_cec__15_30cm__mean
soil_cec__5_15cm__mean
