# Supplementary material for: Integrating Biosynthetic, Genomic and Ecological Open Data for Medicinal Plant Research: A Leakage-Aware Evidence-Prioritization Framework
Source: Int J Mol Sci. 2026 Jul 29;27(15):6801. doi: 10.3390/ijms27156801 (PMC13467606; doi:10.3390/ijms27156801)
Supplement: Supplementary file 1 [file ijms-27-06801-s001.zip › med plant data revised notebooks/soilgrids_notebook3_output.zip!/figures/figureS_balanced_soil_block_sensitivity.pdf]

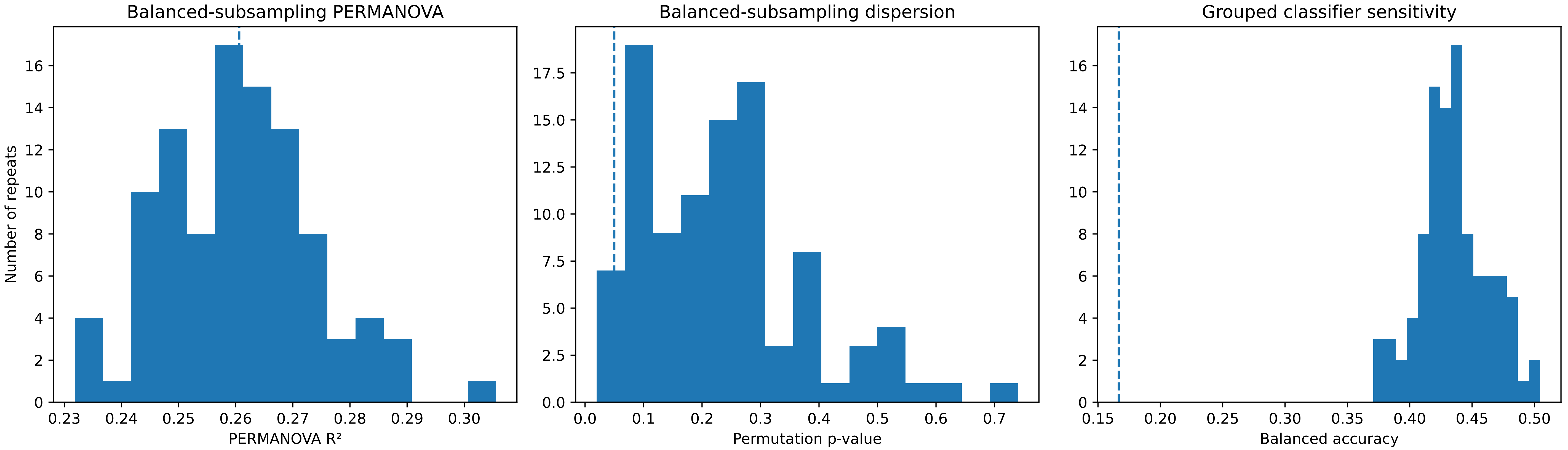
Balanced-subsampling PERMANOVA
Number of repeats
PERMANOVA R²
Balanced-subsampling dispersion
Permutation p-value
Grouped classifier sensitivity
Balanced accuracy
