# Supplementary material for: Integrating Biosynthetic, Genomic and Ecological Open Data for Medicinal Plant Research: A Leakage-Aware Evidence-Prioritization Framework
Source: Int J Mol Sci. 2026 Jul 29;27(15):6801. doi: 10.3390/ijms27156801 (PMC13467606; doi:10.3390/ijms27156801)
Supplement: Supplementary file 1 [file ijms-27-06801-s001.zip › med plant data revised notebooks/soilgrids_notebook3_output.zip!/figures/compound_evidence_annotation_heatmap.pdf]

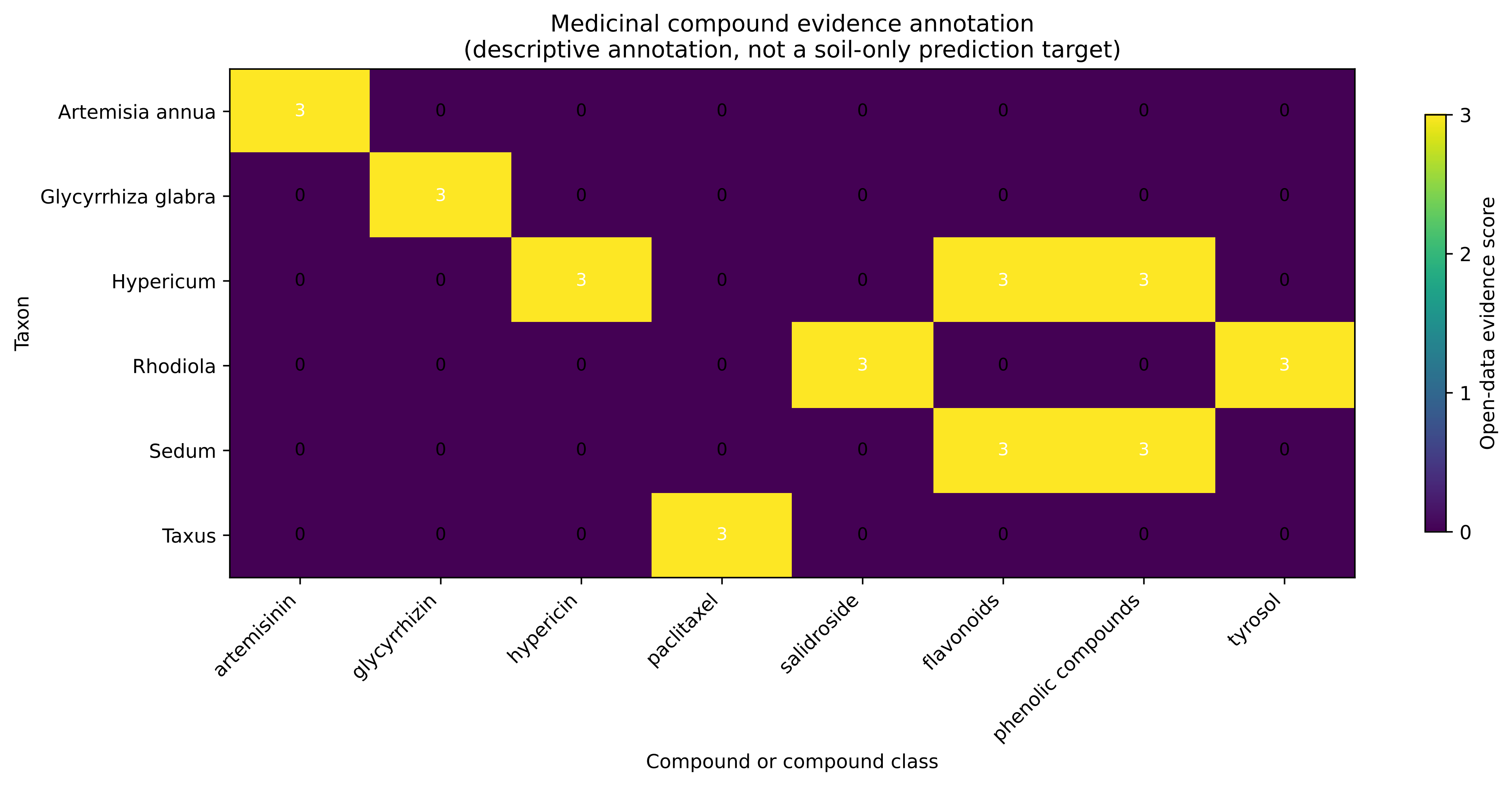

Medicinal compound evidence annotation
(descriptive annotation, not a soil-only prediction target)
Taxon
Artemisia annua
Glycyrrhiza glabra
Hypericum
Rhodiola
Sedum
Taxus
3 0 0 0 0 0 0 0
0 3 0 0 0 0 0 0
0 0 3 0 0 3 3 0
0 0 0 0 3 0 0 3
0 0 0 0 0 3 3 0
0 0 0 3 0 0 0 0
artemisinin
glycyrrhizin
hypericin
paclitaxel
salidroside
flavonoids
phenolic compounds
tyrosol
Compound or compound class
Open-data evidence score
0
1
2
3
