# Supplementary material for: Integrating Biosynthetic, Genomic and Ecological Open Data for Medicinal Plant Research: A Leakage-Aware Evidence-Prioritization Framework
Source: Int J Mol Sci. 2026 Jul 29;27(15):6801. doi: 10.3390/ijms27156801 (PMC13467606; doi:10.3390/ijms27156801)
Supplement: Supplementary file 1 [file ijms-27-06801-s001.zip › med plant data revised notebooks/soilgrids_notebook3_output.zip!/figures/figureS2_soil_niche_profile_by_taxon.pdf]

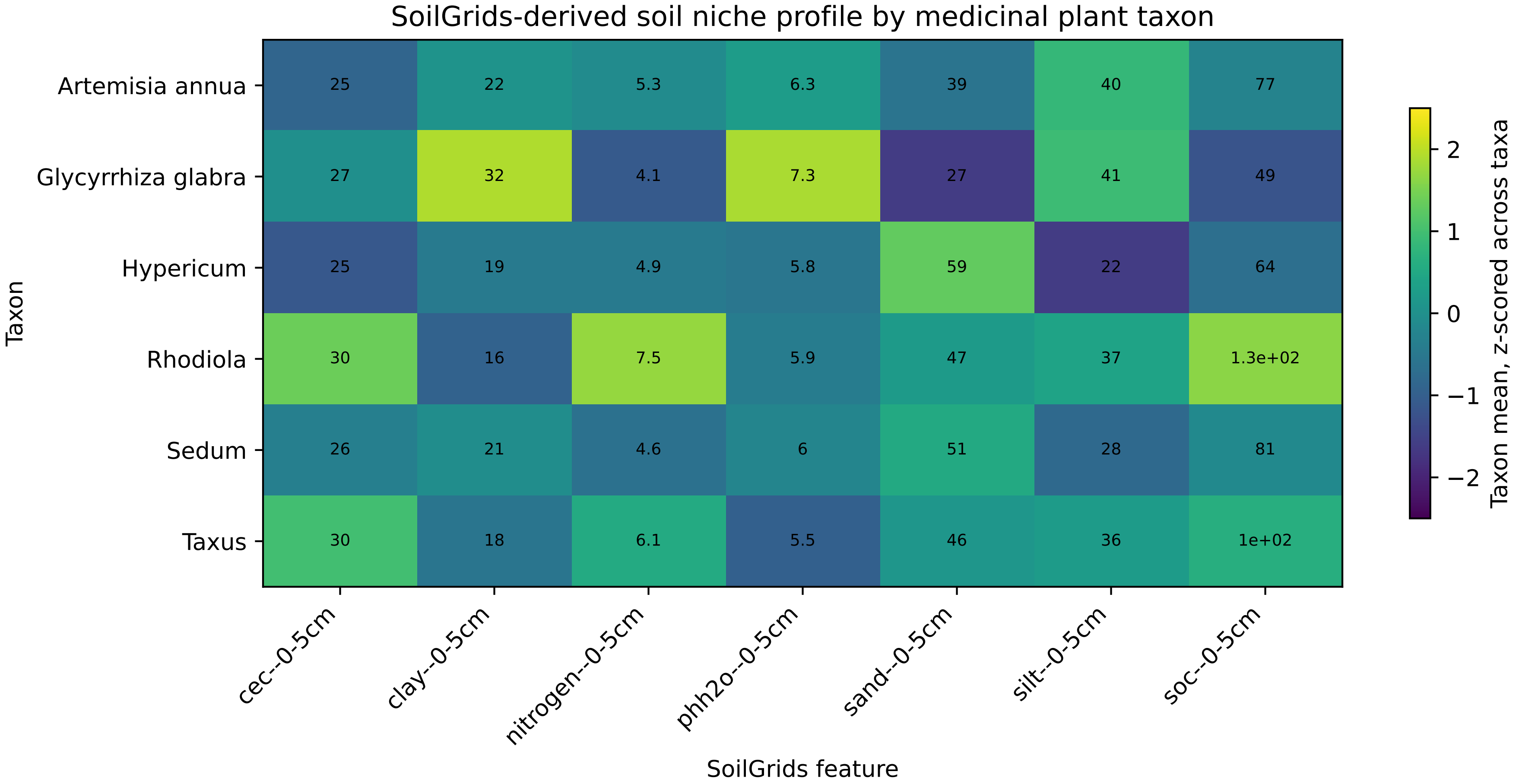

SoilGrids-derived soil niche profile by medicinal plant taxon
Taxon
Artemisia annua
Glycyrrhiza glabra
Hypericum
Rhodiola
Sedum
Taxus
25
22
5.3
6.3
39
40
77
27
32
4.1
7.3
27
41
49
25
19
4.9
5.8
59
22
64
30
16
7.5
5.9
47
37
1.3e+02
26
21
4.6
6
51
28
81
30
18
6.1
5.5
46
36
1e+02
cec--0-5cm
clay--0-5cm
nitrogen--0-5cm
phh2o--0-5cm
sand--0-5cm
silt--0-5cm
soc--0-5cm
SoilGrids feature
Taxon mean, z-scored across taxa
2
1
0
−1
−2
